# Novel Re(I) Complexes as Potential Selective Theranostic Agents in Cancer Cells and *In Vivo* in *Caenorhabditis elegans* Tumoral Strains

**DOI:** 10.1021/acs.jmedchem.3c01869

**Published:** 2024-03-07

**Authors:** Alicia Marco, Pezhman Ashoo, Samanta Hernández-García, Pedro Martínez-Rodríguez, Natalia Cutillas, Annette Vollrath, Dustin Jordan, Christoph Janiak, Fernando Gandía-Herrero, José Ruiz

**Affiliations:** † Departamento de Química Inorgánica, Universidad de Murcia, and Institute for Bio-Health Research of Murcia (IMIB-Arrixaca), E-30100 Murcia, Spain; ‡ Departamento de Bioquímica y Biología Molecular A. Unidad Docente de Biología, Facultad de Veterinaria, Universidad de Murcia, E-30100 Murcia, Spain; § Institut für Anorganische Chemie und Strukturchemie, Heinrich-Heine-Universität Düsseldorf, Universitätsstrasse 1, D-40225 Düsseldorf, Germany

## Abstract

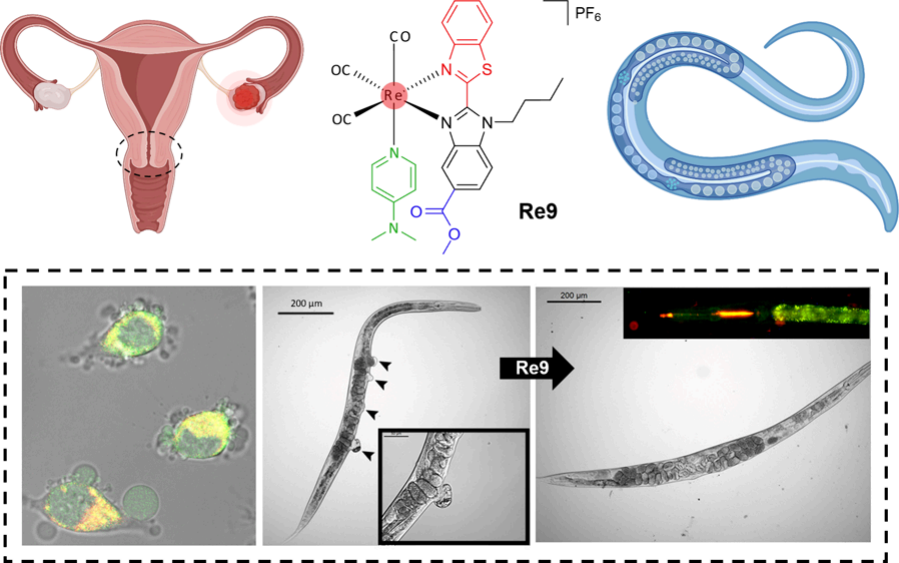

A series of rhenium(I) complexes of the type *fac*-[Re(CO)_3_(N^N)L]^0/+^, **Re1**–**Re9**, was synthesized, where N^N = benzimidazole-derived bidentate ligand with an ester functionality and L = chloride or pyridine-type ligand. The new compounds demonstrated potent activity toward ovarian A2780 cancer cells. The most active complexes, **Re7**–**Re9**, incorporating 4-NMe_2_py, exhibited remarkable activity in 3D HeLa spheroids. The emission in the red region of **Re9**, which contains an electron-deficient benzothiazole moiety, allowed its operability as a bioimaging tool for *in vitro* and *in vivo* visualization. **Re9** effectivity was tested in two different *C. elegans* tumoral strains, JK1466 and MT2124, to broaden the oncogenic pathways studied. The results showed that **Re9** was able to reduce the tumor growth in both strains by increasing the ROS production inside the cells. Moreover, the selectivity of the compound toward cancerous cells was remarkable as it did not affect neither the development nor the progeny of the nematodes.

## Introduction

Cancer stands as the second leading cause of death worldwide, contributing to a significant global disease burden with approximately 10 million annual deaths.^1,2^ Conventional cancer treatments, such as surgery, radiotherapy, and chemotherapy,^3^ have notable limitations, including severe side effects on healthy organs and drug resistance.^4^ Consequently, discovering of alternative and more selective anticancer drugs is a highly desirable goal and remains to be an active field of research, where metallopharmaceuticals are playing a significant role.^5−12^ Smart drugs that provide a combination of diagnostics and therapy, “theranostic agents”, are of recent origin and have also received a surge of research interest.^13,14^ Radioactive ^186^Re and ^188^Re have been extensively used in clinical treatment of cancer,^15,16^ and in the past decade, there has been an enormous interest in the exploitation of the rich photophysical properties of rhenium complexes for diverse imaging modalities and therapeutic biomedical applications.^17−22^ In particular, rhenium carbonyl complexes have been shown to be novel anticancer agents,^23,24^ inhibitors of the SARS-CoV-2 main protease,^25,26^ and more recently therapeutic agents for schemia-reperfusion injury (IRI).^27^ In general, the mechanisms of action of Re(I) anticancer complexes containing the *fac*-[Re(CO)_3_]^+^ core are quite distinct from that of conventional platinum agents,^23,28−34^ that is dependent on covalent bond formation to DNA.^35^ Targeting vulnerable organelles, such as the mitochondria, is one strategy that has been employed to combat resistance to chemotherapeutics,^36^ and that generally is the case for rhenium(I) tricarbonyl complexes.^33,36−43^ However, their *in vivo* antitumor efficacy is still little known.^34,44^ Our previous results on ruthenium(II) and iridium(III) organometallic complexes of the types [Ru(C^N)(N^N)_2_]^+^, [Ir(C^N)_2_(N^N)]^+^, and [Ir(C^N)(N^N^N)]^+^ demonstrated that slight modifications on the benzimidazole-based ligand core rendered high anticancer activities *in vitro*,^45−49^ the N-substituted butyl group serving to adjust the lipophilic properties and enhance cellular uptake and targeting preferentially mitochondria,^45,46^ whereas the ester group attached to the benzimidazole backbone could act as a handle for further functionalization.^50^

**Scheme 1:**
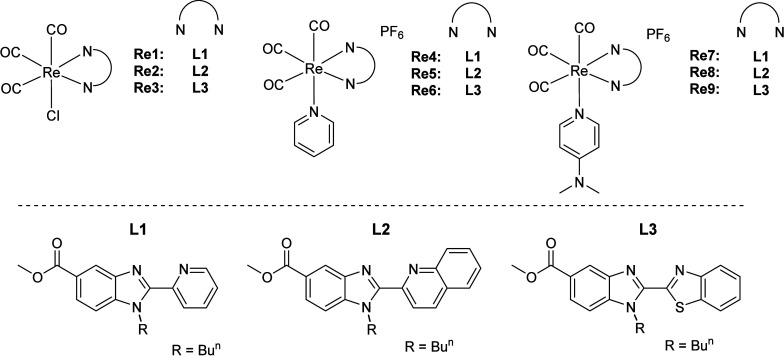
Structures of the New Rhenium(I) Tested Compounds

Herein, we report a new series of nine rhenium(I) complexes of the type *fac*-[Re(CO)_3_(N^N)L]^0/+^ (Scheme 1) to introduce new metal-based compounds for effective and selective inhibition of cancer, where N^N is a modified benzimidazole-based bidentate ligand. The election of ligand **L3** was based on the thought that the electron-deficient nature of the benzothiazole moiety could led to a red-shift of the emission maximum of the Re complexes compared to that of those containing the 2-pyridyl-benzimidazole ligand **L1**.^48^ The most active complexes **Re7**–**Re9** exhibited potent activity in 3D multicellular HeLa tumor spheroids, indicating potential efficacy against solid tumors. Recently, *Caenorhabditis elegans* tumoral strain JK1466 was used to study the biological activity and the mechanism of action *in vivo* of some organic and metal-based drugs.^48,51^ In this work, another strain, MT2124, is included along with the JK1466 strain to increase the types of cancer modeled. The results showed that **Re9** reduced the tumoral cell proliferation in both strains, without hindering the nematodes normal development and life cycle, thus showing high selectivity toward cancerous cells. Further analysis revealed that **Re9**’s mechanism of action is linked to its capacity to elevate intracellular ROS levels in both cancer cells and the *C. elegans* tumoral strains. This unique feature contributes to its potential as an effective anticancer agent. This study highlights the promising properties of these rhenium complexes and warrants further exploration of their molecular mechanisms and potential applications in preclinical and clinical settings for the treatment of various malignancies.

## Results and Discussion

### Synthesis and Characterization of Re(I) Complexes (**Re1**–**Re9**)

The N^N benzimidazole ligands **L1**–**L3** were prepared by condensation reactions between the diamine containing the ester and the butyl group (methyl 3-amino-4-(butylamino)benzoate) and the corresponding aldehyde (2-pyridinecarboxaldehyde, 2-quinolinecarboxaldehyde or benzothiazole-2-carboxaldehyde), as previously reported by our group (Scheme S1).^48,52^ Replacement of Cl by py or 4-NMe_2_py could modulate the anticancer potency of Re(I) complexes, as shown previously in some Ru(II) and Ir(III) half-sandwich complexes.^53,54^

**Scheme 2:**
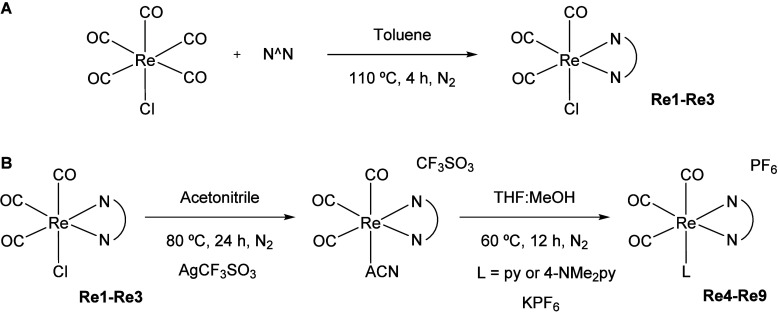
Synthesis of (A) Neutral Complexes **Re1**–**Re3** and (B) Cationic Complexes **Re4**–**Re9**

The synthesis of the different complexes was carried out by adaptation of procedures reported in the literature (Scheme 2).^55,56^ Neutral complexes **Re1**–**Re3** were prepared by the reaction of Re(CO)_5_Cl and the corresponding N^N ligand (Scheme 2A). The cationic complexes **Re4**–**Re9** were synthesized in two steps (Scheme 2B): the reaction of the corresponding Re(CO)_3_(N^N)Cl precursor with the AgCF_3_SO_3_ salt to obtain the nonisolated dechlorinated intermediate, and then its reaction with py or 4-NMe_2_py, followed by the exchange of the counteranion with excess of KPF_6_.^38^

The new yellow to orange rhenium complexes **Re1**–**Re9** are shown in Scheme 1. All complexes were characterized using multinuclear ^1^H and ^13^C{^1^H} NMR spectroscopy (see Figures S1–S18 in the Supporting Information) and IR spectroscopy (Figures S19–S27). The ^1^H NMR spectra of all complexes show the separate aromatics resonances between δ 9.5 and 6.0 ppm, whereas the aliphatic characteristic resonances of the N^N ligands, ester, and butyl groups were found between δ 5.0 and 0.5 ppm. The singlet resonance at δ 2.8 ppm for complexes **Re7**–**Re9** was assigned to the dimethylamine protons. The IR spectra of all complexes (Figures S19–S27) exhibited bands in the region of 2030–1890 cm^–1^ due to symmetric and asymmetric stretching of carbonyl groups, which are indicative of the *fac*-stereochemistry of carbonyl groups around the metal center in complexes of the type *fac*-[Re(CO)_3_(N^N)L]^0/+^.^57^ Final evidence of the correct formation of the compounds has been obtained from the high-resolution mass spectra with the identification of the molecular peaks corresponding to [M+NH_4_]^+^ and [M-Cl]^+^ in the case of **Re1**–**Re3** complexes and with the expected isotopic distribution (Figures S28–S30), whereas the cationic complexes **Re4**–**Re9** displayed the corresponding [M–PF_6_]^+^ peaks (Figures S33–S34). The purity of complexes was checked by elemental analysis of C, H, N, and S. It was also confirmed that the purity of complexes was higher than 95% through RP-HPLC/MS in ACN/H_2_O (Table S1 and Figures S31–S34).

### Crystal Structures by X-ray Diffraction

**Figure 1:**
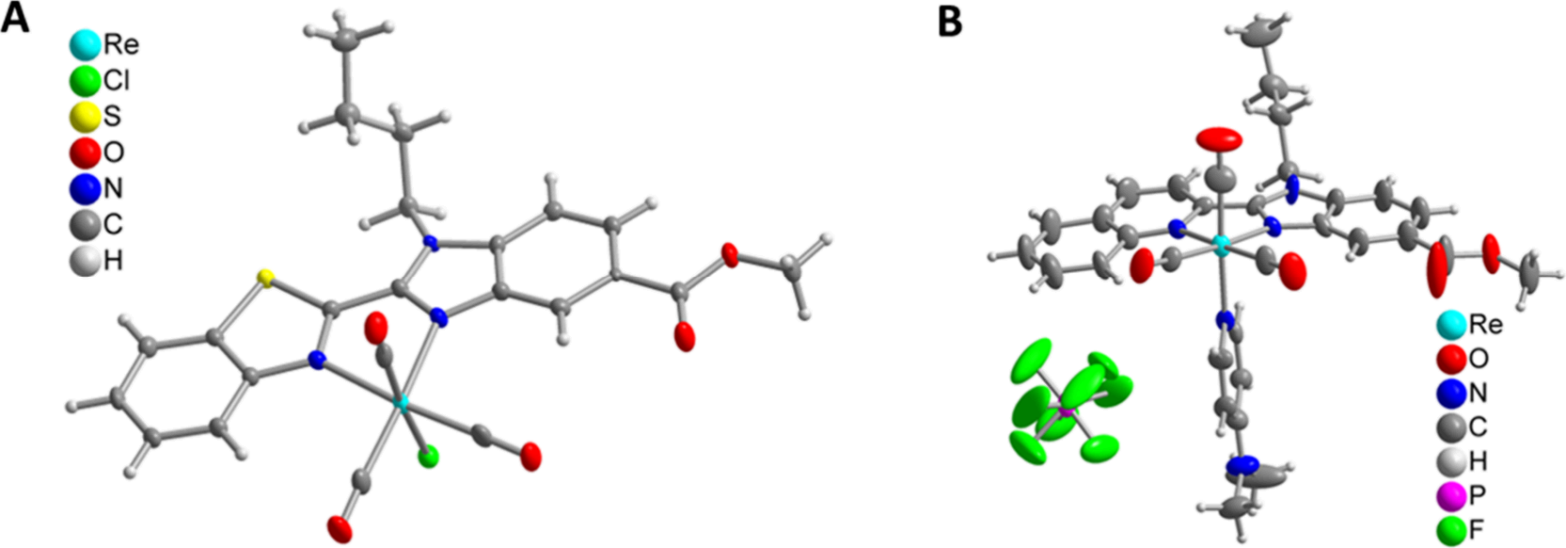
Molecular structures of (A) complex **Re3** and (B) complex **Re8**. Details of π–π interactions, including the symmetry transformations are given in Tables S4 and S6. CCDC reference numbers are 2282513 for **Re3**, 2325369 for **Re3**·CHCl_3_, and 2282514 for **Re8**.

The crystal for the X-ray structure of **Re3** could be fortuitously grown upon slow solvent evaporation from an NMR tube of a solution of **Re3** in CDCl_3_. This structure has no solvent molecules embedded (Figure 1A, Table S2a,b). When the crystals of **Re3** are grown from a CHCl_3_ solution with overlayering of hexane or upon solvent evaporation over a few days, they are obtained as very small needles. Two data sets from two very tiny needle fragments gave the structure of **Re3** as a CHCl_3_ solvate (see Figure S35, Section 6 of the Supporting Information, and Table S2c,d for details). Single crystals for X-ray diffraction analysis of **Re8** were obtained from the slow diffusion of hexane into a saturated solution in acetonitrile. Crystallographic data and selected metrical parameters for **Re3** and **Re8** are given in Tables S2 and S3, respectively. Perspective views of the complexes **Re3** and **Re8** are shown in Figure 1. The rhenium(I) centers adopted a distorted octahedral geometry with the metal ion bound to the benzimidazole-based ligand in a bidentate fashion, with the remaining Re(I) coordination sphere occupied by three carbonyl ligands arranged in a *facial* orientation and an axial chloride ion (for **Re3**) or NMe_2_py molecule (for **Re8**). Rotationally disordered PF_6_ anions charge balances the overall monocationic charge of the complex in the case of **Re8**. There are additional disordered CH_2_Cl_2_ solvent molecules in the crystal structure of **Re8**. The bond lengths and angles, including the bite angles (N–Re–N = 73.5(1)° for **Re3**) were normal (Table S2b).^58^ Both crystal structures are stabilized by inter- and intramolecular interactions (see Tables S4–S7, Schemes S2 and S3, and Figures S36 and S37 in the Supporting Information (SI) for discussion and illustration). The supramolecular packing interactions have been analyzed with PLATON. The π–π interactions between the N^N ligands of **Re3** and **Re8** are shown in Figures S36 and S37, respectively,^59,60^ with the shortest distance between centroids for **Re3** of 3.590 Å.

### Photophysical Characterization of the Compounds

**Figure 2:**
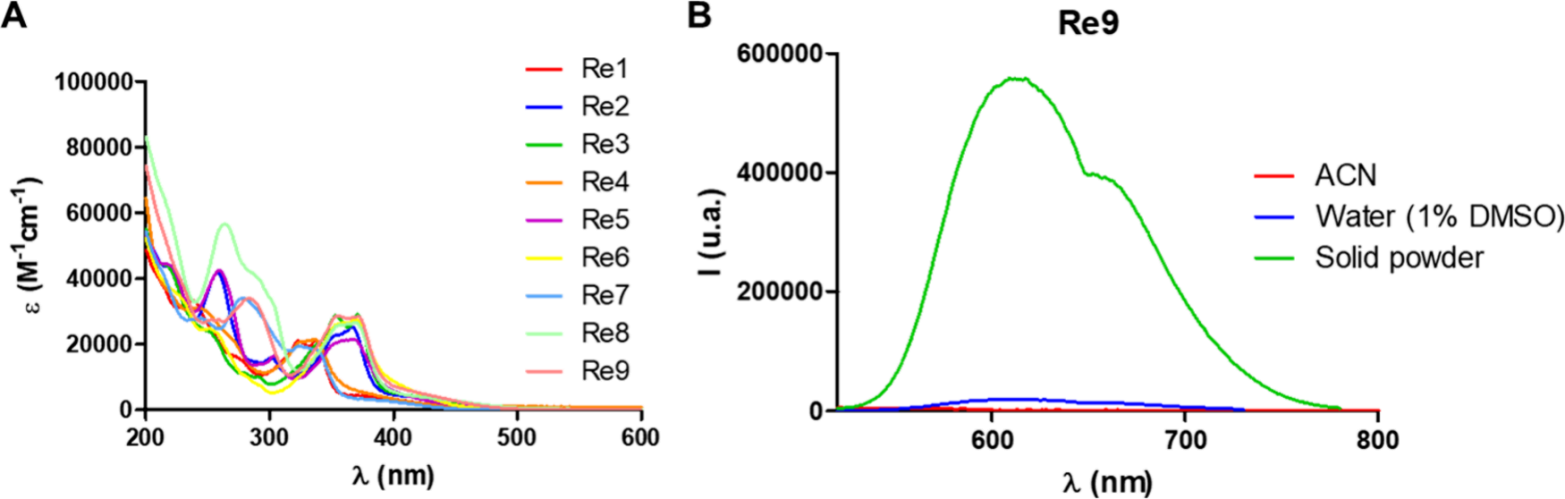
(A) Absorption spectra of complexes **Re1**–**Re9** in aerated acetonitrile (10 μM) at 20 °C. (B) Emission spectra of complex **Re9** in acetonitrile and water (1% DMSO) solution (10 μM) and in the solid state.

The UV/vis absorption and emission spectra of the complexes **Re1**–**Re9** were recorded in acetonitrile (Figure 2A and Figure S39A, respectively) and water (1% DMSO) (Figures S38 and S39B) at room temperature. All complexes showed intense high-energy absorption bands in the range of 260–280 nm corresponding to spin-allowed intraligand π–π* transitions. The lower-energy bands at ca. 360–450 nm correspond to the metal-to-ligand charge-transfer (MLCT) transition (Table S8).^61^ It is also well-known that many Re(I) complexes of the type *fac*-[Re(CO)_3_(diimine)X], where X represents a halogen, present phosphorescent emission due to a metal-to-ligand charge transfer (^3^MLCT) transition involving the orbitals of the accepting diimine ligand,^62^ so the luminescence of complexes **Re1**–**Re9** was studied in acetonitrile, aqueous solution, and solid powder (Figure S39). Upon excitation at the wavelength of their maximum absorption (320–380 nm), all complexes were rather poor emitters both in acetonitrile and water, ranging their maximum wavelength emission from ca. 570 to 625 nm (Figure S39A,B). The emission quantum yields of **Re1**–**Re9** were measured in deaerated acetonitrile, the values being less than 1% in all complexes, except for **Re4**, which showed a 2.3% value. The photophysical data are summarized in Table S8.

Important to note, in the solid state, all the complexes were good emitters as shown in Figure 2B for **Re9** (and Figure S39C for **Re1**–**Re9**), suggesting that the emission might be originated from molecular aggregation in the rigid media through π–π stacking of the chelating ligands (Figure 2B for **Re9**). It was also observed that in solution and solid state, complexes containing the **L1** ligand showed maximum wavelength emission at 550–570 nm, while complexes containing **L2** or **L3** showed a bathochromic shift to 605–630 nm, probably due to the increased π-conjugation of the N^N ligand.^63^

### Stability in Solution

Aquation of monodentate chloride ligand is a common behavior for metallodrugs and is usually considered an activation step,^64^ and some interesting aqua Re(I) carbonyl complexes with high cytotoxicity have been recently reported.^24^ So, the evolution of the chlorido complex **Re1** (1 mM) in methanol-*d*_4_, containing adventitious water, was monitored by 400 MHz ^1^H NMR spectroscopy at 25 °C at different time points (from day 0 to day 3). As shown in Figure S40, duplication of the peaks in the aromatic region (assigned to the chelating ligand **L1**) was observed. Important to note, after the addition of an excess of sodium chloride (100 mM) to the tube, the initial unique set of resonances of the chelating ligand was observed (Figure S40 top), indicating that the hydrolysis was reversible and that **Re1** did not suffer from decomposition or chelating ligand dissociation.

**Figure 3:**
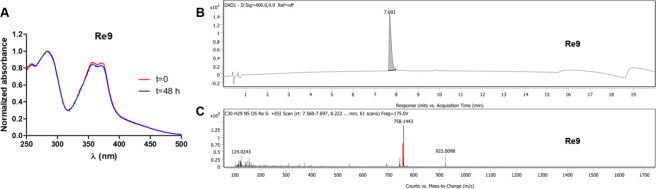
(A) Time evolution of the absorbance spectrum of complex **Re9** (10 μM) in RPMI (5% DMSO). (B) RP-HPLC chromatogram of complex **Re9** (UV detection at 400 nm), using acetonitrile:water in gradient mode as a mobile phase (0.05% acetic acid), and (C) the corresponding mass spectra showing the [M–PF_6_]^+^ peak.

The stability of the complexes **Re1**–**Re9** in DMSO was investigated by UV/vis spectroscopy at different times of incubation at 37 °C. As shown in Figure S41, complex **Re5** showed a displacement in the absorption bands after 48 h in DMSO. The partial substitution of the pyridine ligand when **Re5** was dissolved in DMSO-*d*_6_ was confirmed by ^1^H NMR (Figure S42). For complexes **Re4** and **Re6**–**Re9**, their UV/vis spectra in DMSO remained unaltered, suggesting that they could be stable in this solvent. The stability of **Re6** and **Re9** was further confirmed in DMSO-*d*_6_ by ^1^H NMR; no changes were observed after 48 h (Figure S43 and S44, respectively). Notably, the UV/vis spectra of complexes **Re4** and **Re6**–**Re9** in RPMI (5% DMSO) displayed no changes after incubation for 48 h at 37 °C (Figure 3A for **Re9** and Figure S45 for **Re1**–**Re8**).

In addition, further evidence of the stability of the complexes **Re4** and **Re6**–**Re9** came from the RP-HPLC studies when using acetonitrile:water as a mobile phase in gradient mode. As shown in Figure 3B, only one single peak was observed in the chromatogram of complex **Re9**, (UV detection at 400 nm), the mass spectra of this peak extracted from the chromatogram indicating that compound **Re9** remains intact (Figure 3C) and suggesting that the (OC)_3_**L3**Re–pyNMe_2_ axial bond is not labile in these conditions. Similar results were found also for complexes **Re4** and **Re6**–**Re8** (Figures S33 and S34).

Important to note, while no isosbestic points were observed during the measurement of UV/vis spectra of the chloride metal complexes **Re1–Re3** in DMSO and no time-dependent decrease in absorbance was noticed at least for 48 h (Figure S41), however, the ESI-MS of freshly prepared solutions of **Re1–Re3** in DMSO showed mass peaks assigned to the formation of the corresponding DMSO adduct [Re^I^(**L**)(CO)_3_(DMSO)]^+^ (Figures S46–S48). So, these results suggest that the replacement of the chlorido ligand by DMSO in complexes **Re1–Re3** could be occurring almost instantaneously.

### Antiproliferative and Cytotoxicity Testing in 2D and 3D Cell Culture Models

The antiproliferative activity of Re(I) compounds **Re1**–**Re9** and cisplatin was evaluated in a series of ovarian and cervix cancer cells as well as a nontumorigenic cell line (Table 1). Overall, **Re7**–**Re9**, with 4-NMe_2_py as the main ligand, exhibited significantly higher cytotoxic activity compared to cisplatin against the studied cancer cells. Notably, the IC_50_ values for **Re9** were found to be less than 1 μM in treated A2780 cells, indicating a remarkable 10-fold higher antiproliferative effect of **Re9** compared to CDDP.

**Table 1:** IC_50_ [μM] Values Determined by the MTT Test for Cancer and Normal Cells Treated with **Re1–Re9** Complexes and Cisplatin after 48 h of Treatment^a^

complexes	HeLa	A2780	BGM	SF^b^
**Re1**	17.3 ± 1.1	7.3 ± 0.3	>100	>13.7
**Re2**	5.0 ± 0.2	5.1 ± 0.1	>100	>19.6
**Re3**	4.4 ± 0.3	5.1 ± 0.4	>100	>19.6
**Re4**	10.6 ± 0.6	2.0 ± 0.1	17.1 ± 1.0	8.6
**Re5**	4.2 ± 0.4	0.89 ± 0.07	10.5 ± 0.3	11.8
**Re6**	5.4 ± 0.3	1.2 ± 0.1	23.0 ± 8.6	19.2
**Re7**	9.1 ± 1.0	0.92 ± 0.08	48.6 ± 2.3	52.8
**Re8**	3.1 ± 0.2	0.49 ± 0.07	9.0 ± 0.4	18.4
**Re9**	1.9 ± 0.1	0.30 ± 0.03	11.3 ± 1.0	37.7
cisplatin	23.3 ± 2.0	2.9 ± 0.4	6.8 ± 0.9	2.3

a The results are expressed as mean values ± SD from at least three independent experiments.

b Selectivity factor (SF) defined as IC_50_ (normal BGM cells)/IC_50_ (tumoral A2780 cells).

**Figure 4:**
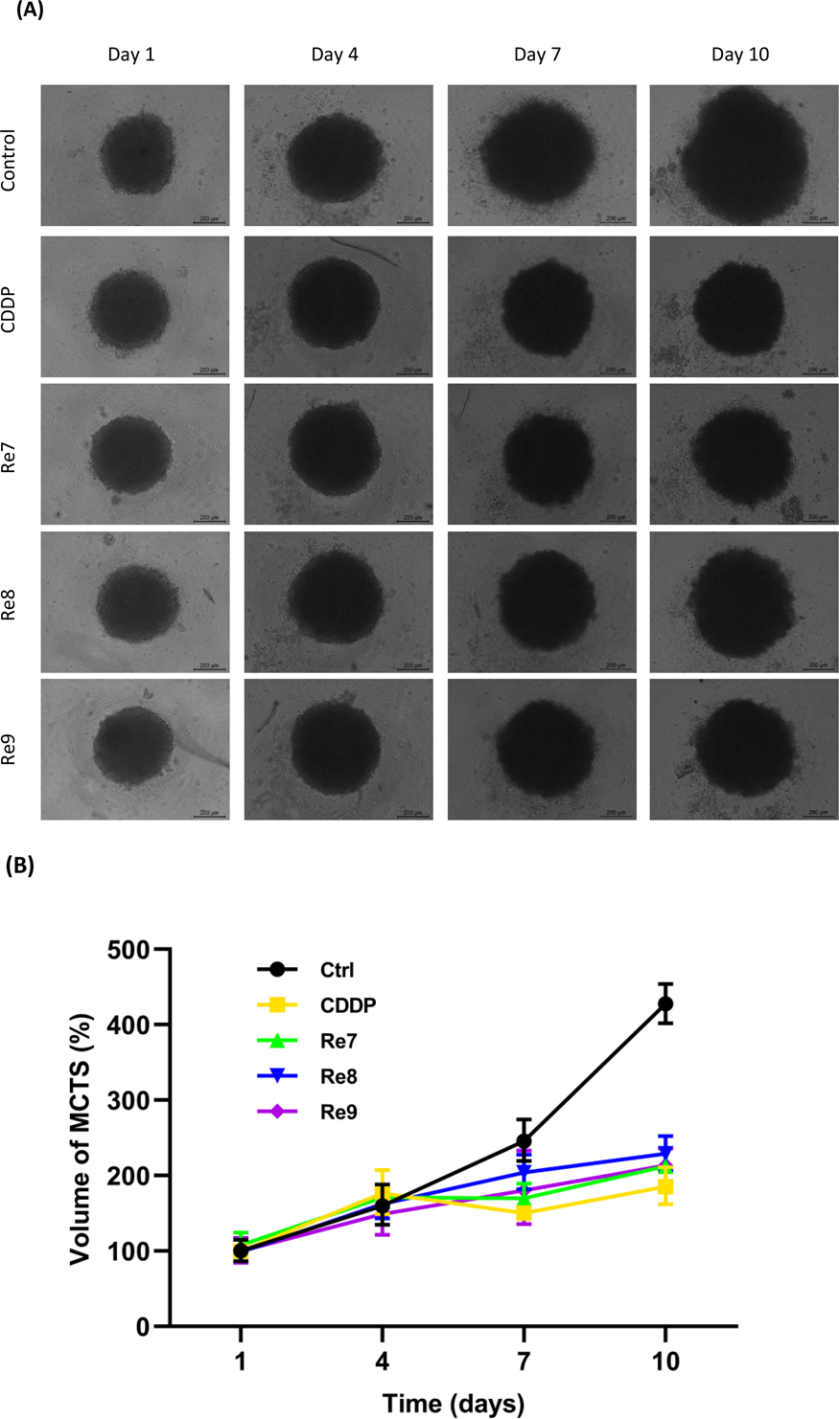
(A) Representative microscopy images of HeLa spheroids treated with **Re7**–**Re9** and cisplatin at their IC_50_ values for 2 h on days 1, 4, and 7. Scale bar: 200 μm. (B) Normalized volume of HeLa multicellular tumorspheres (MCTS) over a span of 10 days. MCTS were treated on days 1, 4, and 7 with **Re7–Re9** and cisplatin at their IC_50_ values for 2 h in each treatment.

Furthermore, we investigated the effect of Re(I) compounds **Re7**–**Re9** and cisplatin on tumor growth in a 3D cell culture model of HeLa MCTS (Figure 4). The formation of tumor spheres was observed on day 1, and the compounds were incubated for 2 h at 37 °C. Following the incubation period, the media was replaced with fresh media. Throughout the experiment, we monitored and measured the volume of the MCTS, revealing a significant decrease in the volume of the treated spheroids compared to control cells on day 10. These results indicate a potent inhibitory effect on tumoral growth by **Re9** and its analogs in the 3D cell culture model. Interestingly, the observed enhanced cytotoxicity of **Re7**–**Re9** in both 2D and 3D cell culture models supports the potential of these compounds as promising candidates for targeted cancer therapy. The potent antiproliferative effect of **Re9**, specifically in A2780 cells, suggests its ability to effectively inhibit cancer cell growth and warrants further investigation to elucidate its underlying mechanism of action. Moreover, our findings in the 3D cell culture model highlight the significance of exploring tumor behavior in more physiologically relevant settings, such as the multicellular tumor spheroids.

### Cellular Uptake with Re(I) Compounds

**Figure 5:**
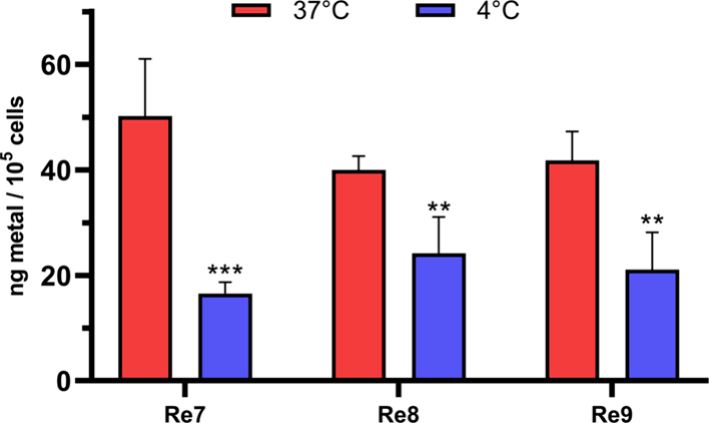
Cellular uptake of Re in A2780 cells after incubation 10 μM of Re(I) compounds **Re7**–**Re9** for 2 h at 37 and 4 °C. Data for intracellular Re concentration represent the mean ± SD from two independent experiments.

Cellular uptake is a critical aspect to investigate the intracellular delivery and potential efficacy of metal-based compounds. In our study, we assessed the content of Re(I) metal within ovarian cancer cells (A2780) upon treatment with **Re7**–**Re9** using inductively coupled plasma mass spectrometry (ICP-MS). The obtained results reveal that **Re7**–**Re9** compounds exhibit notable uptake by A2780 cells (Figure 5), indicating their ability to penetrate the cellular membrane and access the intracellular space. To gain further insights into the mechanism of cellular uptake, we explored the temperature dependence of the Re(I) compound internalization. Notably, we observed a significant reduction in Re accumulation when A2780 cells were incubated at a low temperature of 4 °C, as compared to cellular uptake at 37 °C. This intriguing finding suggests that the uptake of **Re7**–**Re9** occurs through an energy-dependent pathway, possibly involving active transport processes, rather than passive diffusion. Such energy-dependent uptake mechanisms are often associated with specific transporters or receptor-mediated processes, ensuring efficient intracellular delivery and targeting.

### Cellular Localization of Re9 by Confocal Microscopy Imaging

**Figure 6:**
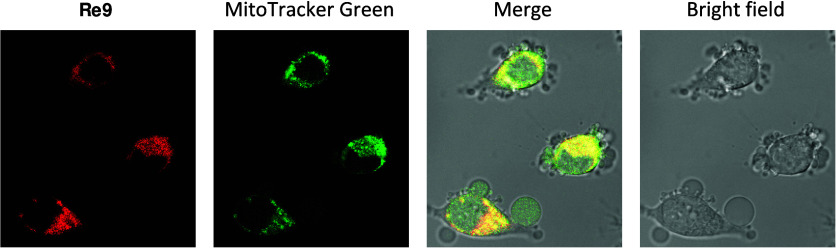
Intracellular colocalization of **Re9** with MTG imaged by confocal laser scanning microscopy. HeLa cells were incubated with 10 μM for 60 min and then stained with MTG (100 nM, 30 min) at 37 °C (**Re9**, λ_ex_= 405 nm and λ_em_ = 620 ± 30 nm; MTG, λ_ex_= 490 nm and λ_em_ = 520 ± 20 nm).

Cellular localization is a critical aspect that governs the pharmacological behavior and therapeutic efficacy of metal complexes. In this study, we sought to unravel the precise subcellular localization of Re(I) compounds by employing confocal microscopy. As shown in Figure 6, the inherent fluorescence signal of **Re9** was clearly observed inside HeLa cells after 1 h at 10 μM. Co-staining experiment was conducted using the mitochondria-specific probe MitoTracker Green (MTG) in HeLa cells. Remarkably, the images obtained from the costaining studies and later analyzed with ImageJ revealed a noteworthy partial overlapping pattern between **Re9** and MTG, suggesting a potential affinity of **Re9** toward the mitochondria. The calculated Pearson’s correlation coefficient for **Re9** and MTG costained HeLa cells was found to be 0.74 ± 0.06. This value indicates a moderate to strong positive correlation, indicating that **Re9** tends to colocalize with mitochondria in living cancer cells. This intriguing observation piqued our interest in exploring the specific interaction of **Re9** with this vital organelle. Notably, as shown in Figure 6, cells exhibited morphological changes similar to pyroptosis (*vide infra*).

### SEM Imaging and Cell Death Induction

**Figure 7:**
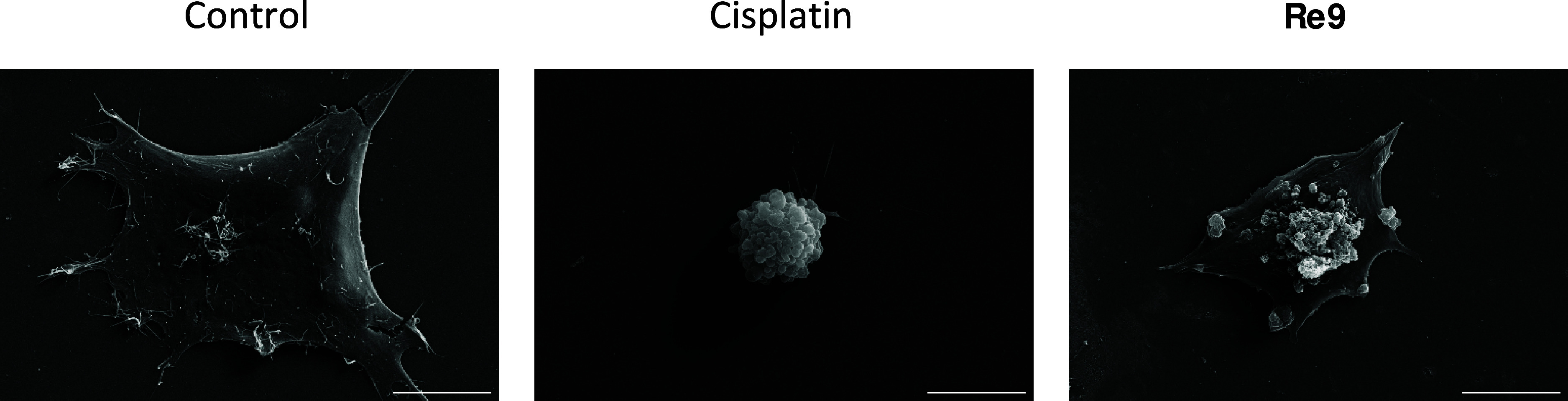
SEM images of A2780 cells. Cisplatin and **Re9** result in apoptosis and pyroptosis, respectively. Scale bar: 10 μM.

Scanning electron microscopy (SEM) analysis uncovered notable alterations in the morphology of cells subjected to **Re9** treatment. These changes were distinctly characterized by a fried egg-like appearance, coupled with flattened cytoplasm, a unique feature commonly associated with pyroptosis, a specific form of programmed cell death. Pyroptosis, contrasting with apoptosis induced by cisplatin, represents a mode of cellular death that involves inflammatory responses and distinct morphological features.^65^ In contrast, cells treated with cisplatin exhibited the formation of large bubbles protruding from the plasma membrane and the entire cell typically displayed swelling, morphological signs commonly associated with apoptosis. The observed difference between the **Re9**-treated cells and both the control cells and those undergoing apoptosis induced by cisplatin is prominently illustrated in Figure 7 and Figure S49.

**Figure 8:**
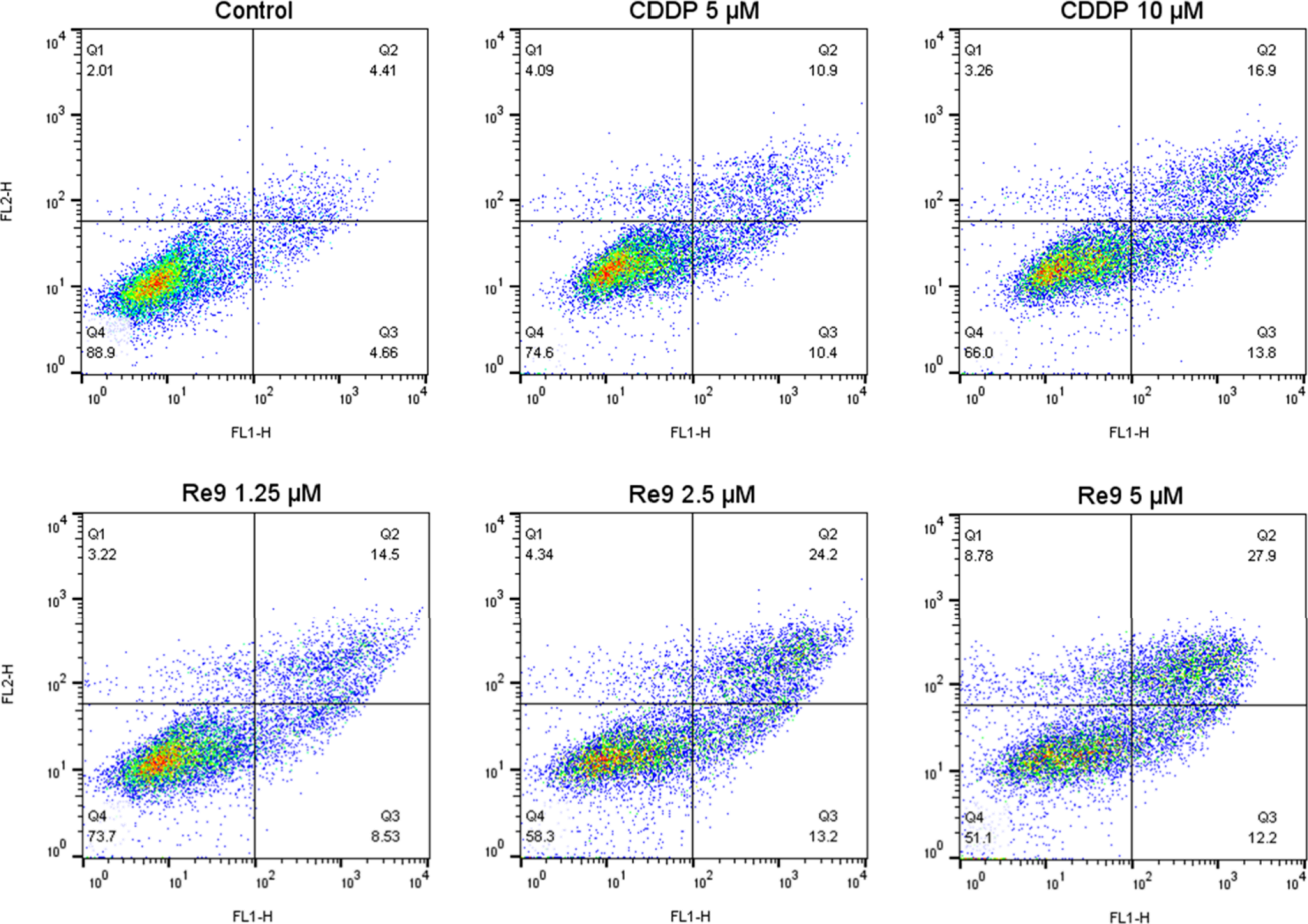
Annexin V-FITC/PI dual staining of A2780 cells treated with compound **Re9** in 1.25, 2.5, and 5 μM after 24 h. Cisplatin has been considered as a positive control. Annexin V–/PI– represents live cells, annexin V+/PI– represents early apoptotic cells, and annexin V+/PI+ denotes late apoptotic or pyroptotic cells.

To more elucidate the main mechanism of cell death induced by **Re9**, the annexin V-FITC/PI dual staining was performed on A2780 cells treated with either **Re9** or cisplatin for 24 h across various concentrations. As shown in Figure 8 and Figure S50, after A2780 cells were incubated with various concentrations of cisplatin and **Re9** for 24 h, the proportion of cells in early apoptosis (Q3) increased from 4.66% in the control group to 13.8% for CDDP (10 μM), 13.2% for **Re9** (2.5 μM), and 12.2% for **Re9** (5 μM). In contrast, the percentage of cells in Q2 changed from 4.41% in the control cells to 16.9, 24.2, and 27.9% for CDDP (10 μM), **Re9** (2.5 μM), and **Re9** (5 μM), respectively. This variation could be attributed to the occurrence of pyroptosis in cells treated with **Re9**, collectively detected with cellular morphological changes and flow cytometry analysis.^66^ An interesting example of pyroptosis induced by PDT treatment with a carbonic anhydrase IX (CAIX)-anchored rhenium(I) conjugate, **CA-Re**, has been recently reported by Mao et al.^67^

**Figure 9:**
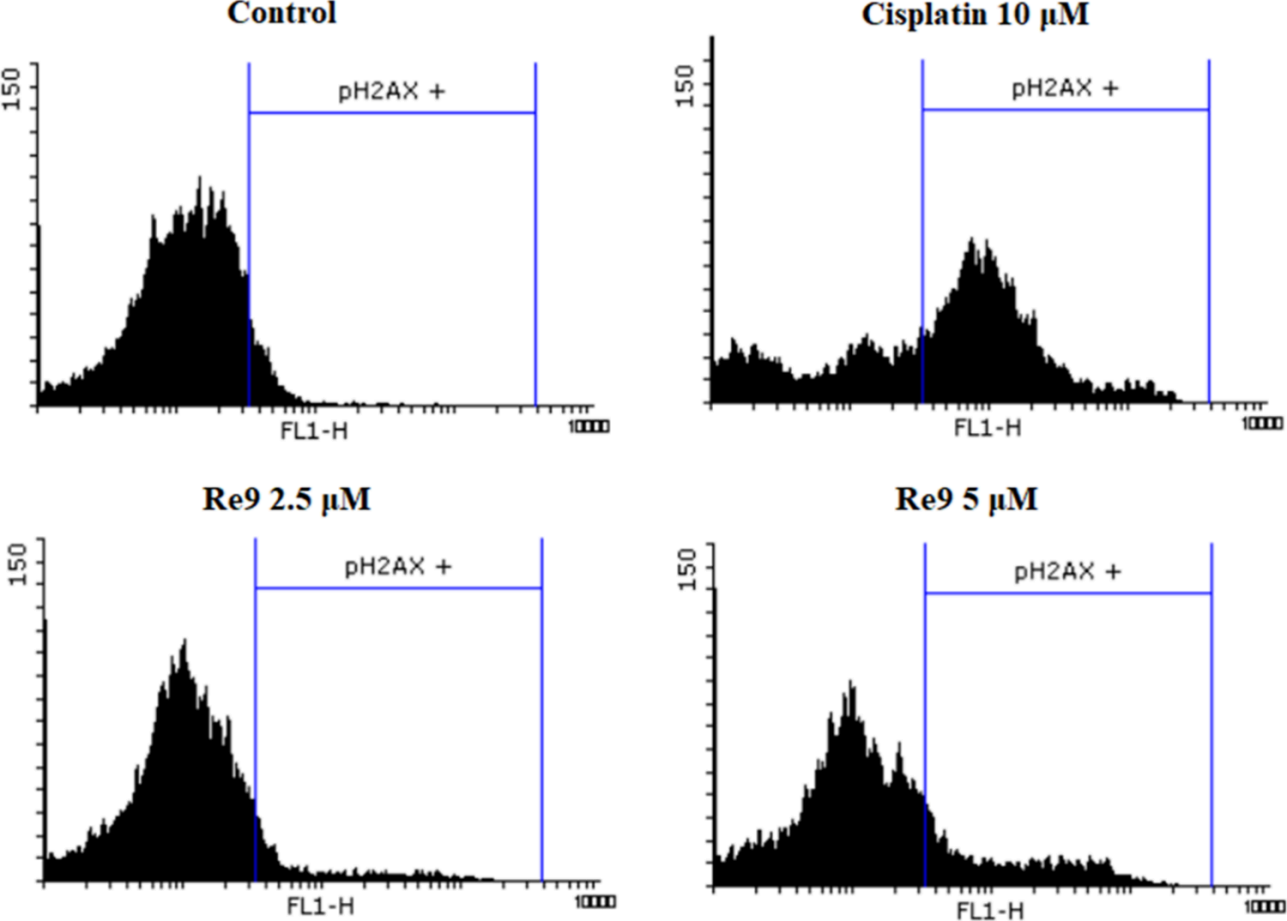
Effect of **Re9** on DNA damage measured by changes in pH2AX staining in the FL1-H channel after 24 h treatment in A2780 cells. Cisplatin was applied as a positive control for DNA damage induction.

For further analyses, we examined the initiation of DNA double-strand breaks using an antiphosphorylated histone H2AX (pH2AX) FITC-conjugated antibody for detection of DNA damage within cells. Intriguingly, the results shown in Figure 9 revealed that treatment with **Re9** resulted in a comparatively minor breakage of DNA in contrast to cisplatin, which significantly contributed to the induction of DNA damage and subsequent apoptosis. At this point, we propose the hypothesis that the limited DNA damage detected in cells treated with **Re9** suggests that the observed cytotoxicity in cancer cells may be attributed to an alternative initiator of programmed cell death, such as the generation of reactive oxygen species (ROS) and/or another type of cell death like pyroptosis, to facilitate this process.

### Intracellular ROS Levels under Normoxia and Hypoxia

**Figure 10:**
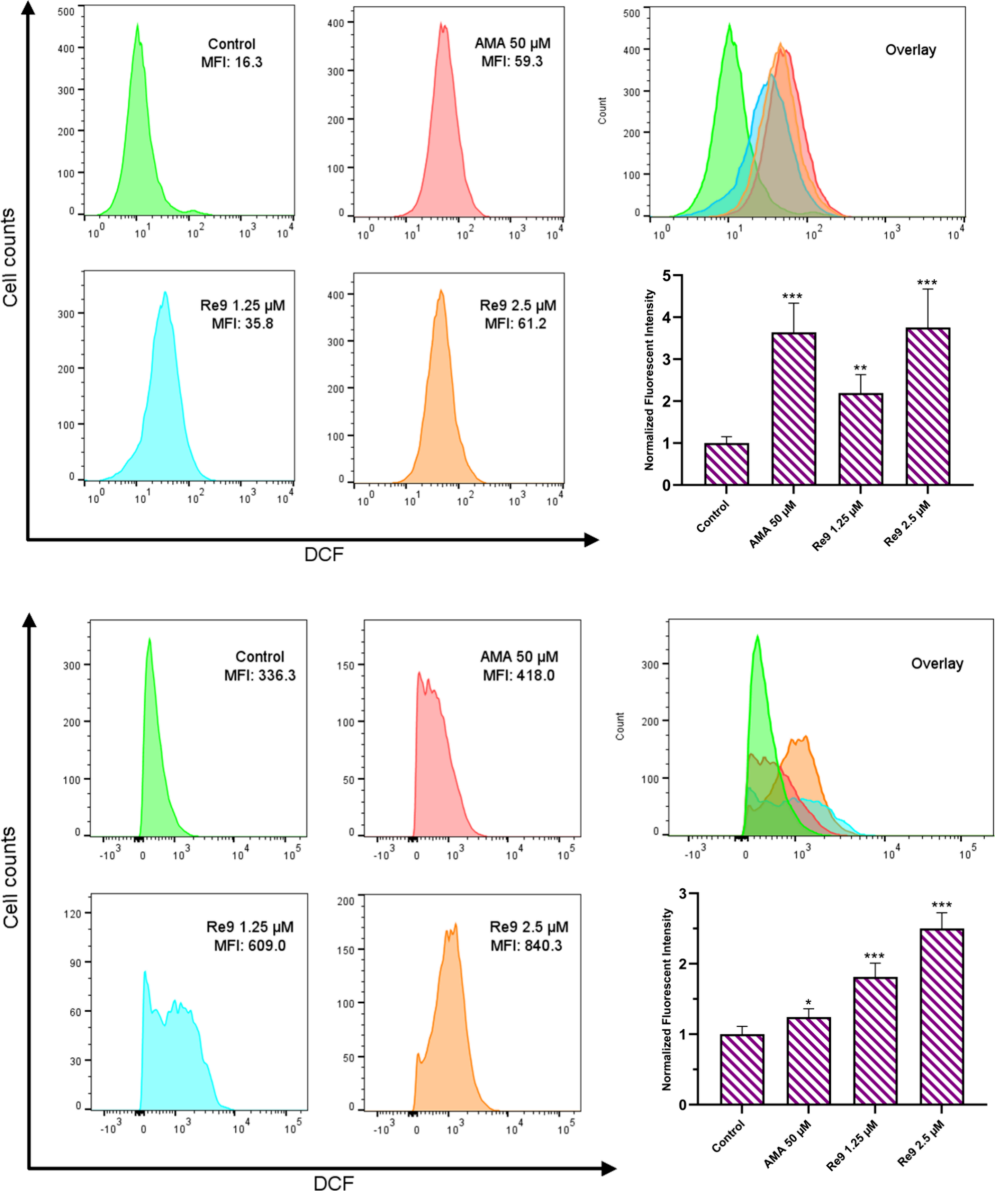
Intracellular ROS generation (A: normoxia (O_2_ 21%) and B: hypoxia (O_2_ 2%)) in **Re9**-treated (1.25 and 2.5 μM, 24 h) A2780 cells measured by flow cytometry (λ_ex_ = 488 nm and λ_em_ = 525 nm). Antimycin A (AMA) is considered as a positive control (50 μM, 6 h). Data expressed as mean ± SD from three replicates. An independent unpaired *t* test was used to define statistical differences between the obtained values (*p < 0.05, **p < 0. 01, ***p < 0.001).

To assess the ability of the mitochondria-targeted compound **Re9** to induce intracellular ROS elevation, flow cytometry with 2′,7′-dichlorofluorescein diacetate (H_2_DCFDA) staining was employed. H_2_DCFDA is a nonfluorescent probe that becomes highly fluorescent upon conversion to 2′,7′-dichlorofluorescein (DCF) in the presence of intracellular ROS. Following a 24 h treatment with **Re9**, a remarkable dose-dependent increase in intracellular ROS levels was observed under both normoxia and hypoxia (Figure 10A,B). Under normoxia conditions, at a concentration of 2.5 μM, the mean fluorescence intensity of DCF in **Re9**-treated cells was approximately 4-fold higher compared to control cells, while in hypoxia, it reduced to 2.5-fold. These compelling findings suggest that **Re9** effectively induces intracellular ROS elevation depending on the amount of oxygen present, leading to ROS-dependent cell death.

### Mitochondrial Membrane Potential Dysfunction

**Figure 11:**
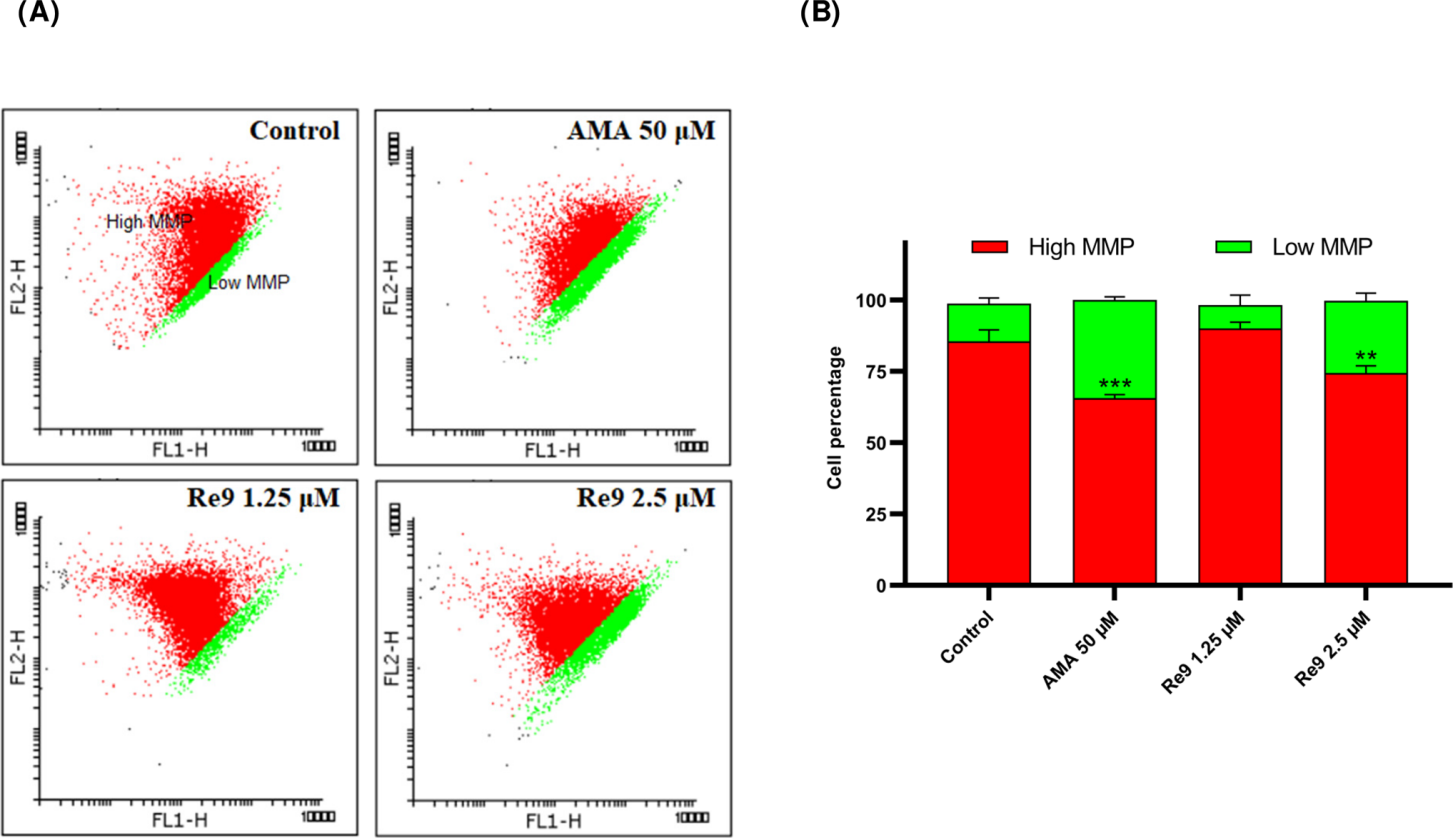
Induction of mitochondrial dysfunction by rhenium complex **Re9**. (A) MMP of **Re9**-treated (24 h) and antimycin A (6 h) A2780 cells analyzed by flow cytometry at indicated concentrations (JC-1 staining, λ_ex_ = 488 nm and λ_em_ = 530 ± 30 nm (green) and 585 ± 30 nm (red)); MMP changes detected as green JC-1 dye monomers (low MMP) or red aggregates (high MMP) in FL1 and FL2 channels. (B) Bar graph presented in percentage of the cells. Data expressed as mean ± SD from three replicates. An independent unpaired *t* test was used to define statistical differences between the obtained values (*p < 0.05, **p < 0. 01, ***p < 0.001).

Mitochondrial membrane potential (MMP) plays a crucial role in regulating cellular processes, and its disruption has been associated with the activation of cell death mechanisms. To investigate the impact of **Re9** on MMP and its potential implications in cellular demise, we investigated the effect of **Re9** on MMP in A2780 cells by performing JC-1 staining after treating the cells with complex **Re9** at concentrations of 1.25 and 2.5 μM for 24 h. Our results (Figure 11) demonstrated a significant decrease in MMP levels following treatment with both **Re9** and the positive control, antimycin A. The reduction in MMP suggests a disturbance in the mitochondrial membrane integrity, implicating mitochondrial dysfunction in the mechanism of **Re9**-induced cytotoxicity. This process might be linked to the generation of reactive oxygen species within the mitochondria and the disruption of membrane integrity mediated by pyroptosis.^68^

### **Re9** Ingestion by *Caenorhabditis elegans*

**Figure 12:**
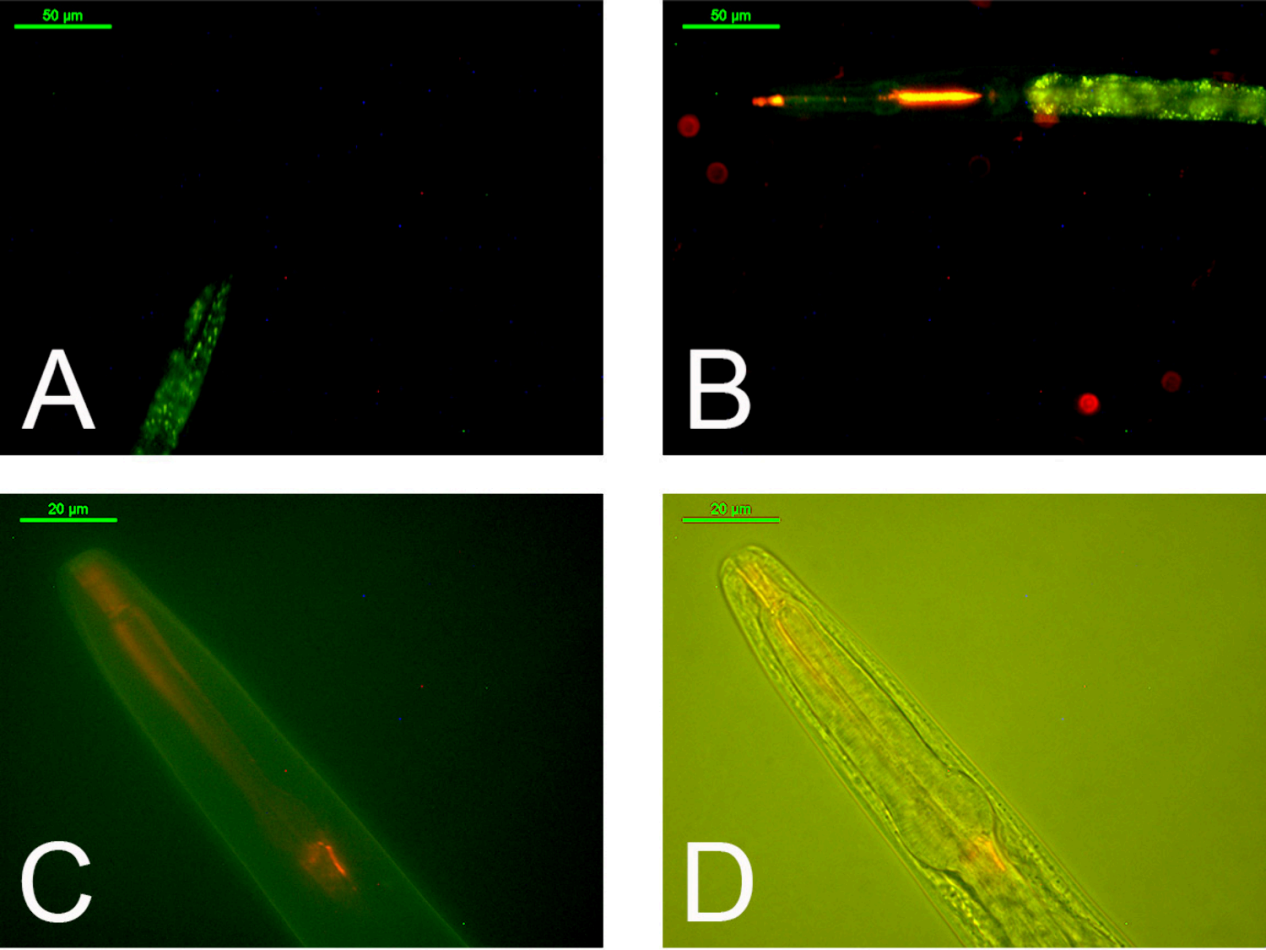
**Re9** ingestion and assimilation by *C. elegans*. (A–D) Representative images of nematodes under fluorescence microscope. (A) Control animal. (B) Nematode treated with **Re9** (150 μM). Scale bar: 50 μm. (C) Closer view under fluorescence of a nematode treated with **Re9** (150 μM) highlighting the pharynx of the animal. (D) Merged image with the brightfield technique. Scale bar: 20 μm.

The model animal *C. elegans* was used to further study the effects of the metal complex that showed better performance *in vitro*, **Re9**. *C. elegans* treated with the complex or with DMSO as a control were visualized under microscope by using fluorescent light (excitation wavelength 480 nm). Due to the intrinsic luminescence of the compound, described above, it was possible to localize the complex into the digestive system of the animal, especially in the pharynx area (Figure 12B–D), an indication of its ingestion and assimilation by the nematode. Control animals did not exhibit any fluorescence in the zone (Figure 12A), supporting the premise that the red luminescence in the pharynx of treated animals was due to **Re9** intake.

### **Re9** effects on *C. elegans* tumor development

**Figure 13:**
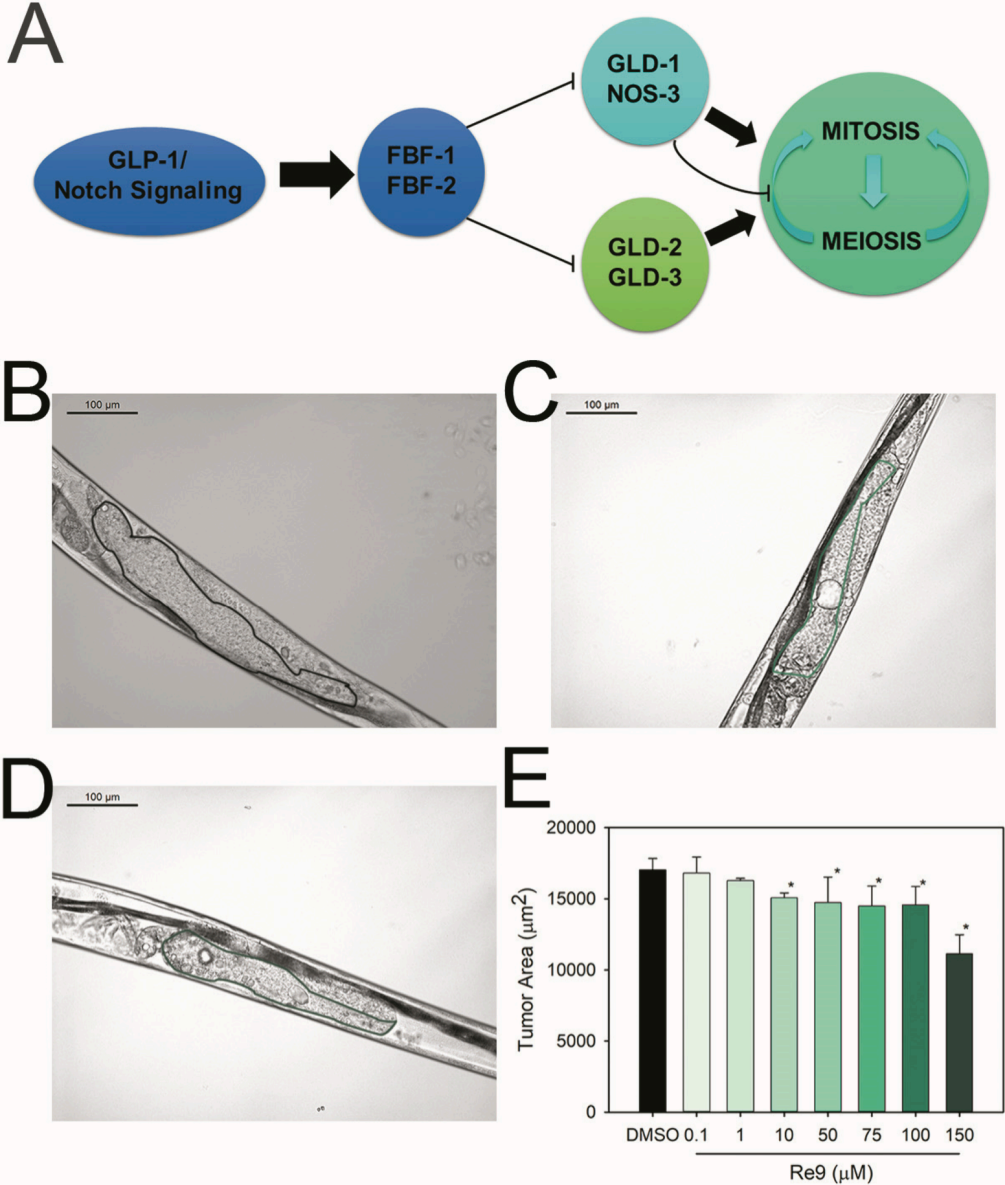
Antitumoral effects of **Re9** on the *C. elegans* strain JK1466. (A) Overview of the regulatory pathway controlling the cell fate decision. (B–D) Representative images of *C. elegans* JK1466 strain gonads. (B) DMSO-treated nematode. (C) **Re9** (10 μM)-treated animal. (D) **Re9** (100 μM)-treated animal. Scale bar: 100 μm. (E) Tumor size evaluation. Two independent assays were performed with *n* ≥ 20. Data is represented as average ± SD * significant at *p* ≤ 0.05 by the ANOVA test.

Oncogenic signaling pathways, such as Notch and Ras, are highly conserved among multicellular organisms; they control many facets of cell proliferation, differentiation, cell cycle progression, cell fate, and cell death. Thus, mutations in these signaling pathways frequently lead to carcinogenesis in humans.^69^ Aberrant function of the Notch signaling pathway has been detected in pancreatic cancer, osteosarcoma, and breast cancer, among others. Alterations in the Ras receptor or its downstream kinases produce the aberrant cell proliferation phenomenon observed in melanoma or hairy cell leukemia. In approximately one-third of all human cancers, Ras is dysregulated.^70^ In *C. elegans*, mutations in these signaling pathways produced several developmental defects, including sterility, infertility, the formation of gonad tumors, and the formation of several pseudovulvas. The mutant strain JK1466 has a loss of function mutation in the *gld-1* gene of the notch signaling pathway (Figure 13A), which controls the transition from mitosis to meiosis of the gonad cells. When *gld-1* is lost, the gonad cells are arrested in mitosis, unable to differentiate, and they accumulate in the gonad, forming tumors lethal to the animals (Figure 13B).^48,51,71^ Nevertheless, **Re9** treatment in a range of concentrations from 10 to 150 μM was able to reduce the size of the tumors by 11.6 and 34.6%, respectively (Figure 13C,D and Table 2). Recent studies showed that cisplatin was able to reduce the tumor size in this strain by 48%.

**Table 2:** *In vivo* Measurements of Tumor Size Using the *C. elegans* Strain JK1466

	C (μM)	*n*	tumor area (μm^2^)	SD	reduction (%)	*p* value vs control
DMSO		144	17041.01	800.56	0.00	
	0.1	53	16817.01	1114.92	1.31	0.161
	1	60	16276.18	167.13	4.49	0.437
	10	74	15073.54	340.20	11.55	<0.001
**Re9**	50	53	14739.35	1784.90	13.51	0.03
	75	58	14502.87	1388.02	14.89	0.003
	100	134	14580.28	1278.45	14.44	<0.001
	150	64	11139.65	1337.46	34.63	<0.001

**Figure 14:**
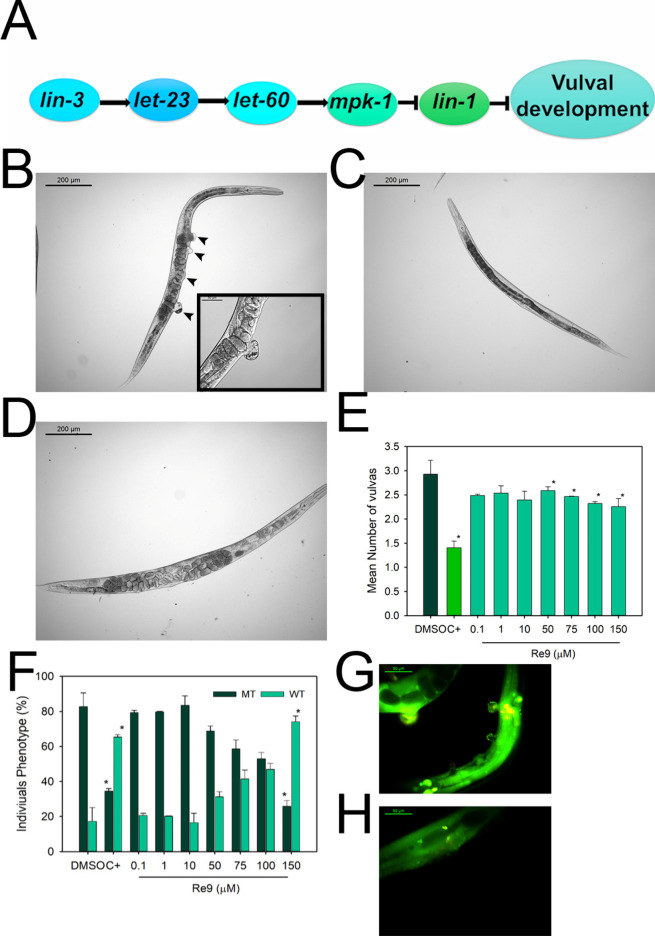
Antitumoral effects of **Re9** on the *C. elegans* strain MT2124. (A) Overview of the regulatory pathway controlling the vulval development. (B–D) Representative images of *C. elegans* MT2124. (B) DMSO treated nematode, arrowheads mark the vulva and the pseudovulvas. In the inset, a magnification of a protruding vulva is shown. (C) Cisplatin (50 μM)-treated animal. (D) **Re9** (150 μM)-treated animal. Scale bar: 200 μm. (E) Average number of vulvas of the multivulva phenotype nematodes. C+ are cisplatin (50 μM)-treated animals. (F) Multivulva phenotype evaluation; MT corresponds to multivulva animals, and WT corresponds to wild-type nematodes; C+ are cisplatin (50 μM)-treated animals. Two independent assays were performed with *n* ≥ 20. Data is represented as average ± SD * significant at *p* ≤ 0.05 by the ANOVA test. (G) MT2124 nematode treated with DMSO and stained with acridine orange. (H) MT2124 nematode treated with **Re9** (150 μM) and stained with acridine orange. Scale bar: 50 μm.

The *C. elegans* strain MT2124 has a loss of function mutation in the gene *let-60* that belongs to the RAS pathway (Figure 14A) and is an ortholog of the human HRas proto-oncogene. *let-60* is required for vulval development, spicule development, or germline meiotic progression, among other functions. MT2124 nematodes have up to four ectopic pseudovulvas,^69^ in addition to the normal vulva, protruding on the ventral side of the worms (Figure 14B,G). **Re9** reduced the MT phenotypic incidence by a 36.0 and a 68.7% at 100 and 150 μM, respectively (Figure 14D,F,H and Table 3). Moreover, the treatment was able to reduce the number of vulvas by 22% (Figure 14E). Meanwhile, cisplatin at 50 μM reduced the incidence by 58.2% and the number of vulvas by 52% (Figure 14C,E,F). The maximum concentration employed for cisplatin was 50 μM since this strain appeared to be extremely sensitive to it; as the complex’s concentration was raised, the nematodes experienced a developmental arrest.

**Table 3:** *In vivo* Antitumoral Effect Evaluation Using the *C. elegans* Strain MT2124

	C (μM)	*n*	WT (%)	SD	MT (%)	SD	*p* value vs control	*n*° vulvas	SD	*p* value vs control
DMSO		277	17.27	7.90	82.73	7.90		2.93	0.29	
CDDP	50	72	65.38	1.40	34.62	1.40	<0.001	1.40	0.14	<0.001
	0.1	135	20.65	1.20	79.35	1.20		2.49	0.02	
	1	159	20.22	0.22	79.78	0.22		2.54	0.15	
	10	120	16.49	5.38	83.51	5.38		2.40	0.18	
**Re9**	50	114	31.25	2.95	68.75	2.95		2.59	0.08	<0.001
	75	116	41.42	5.16	58.58	5.16		2.47	0.01	<0.001
	100	253	46.92	3.50	53.08	3.50	<0.001	2.32	0.04	<0.001
	150	112	74.13	3.31	25.87	3.31	<0.001	2.26	0.17	<0.001

### Re9 and CDDP Effects on *C. elegans* Size

**Figure 15:**
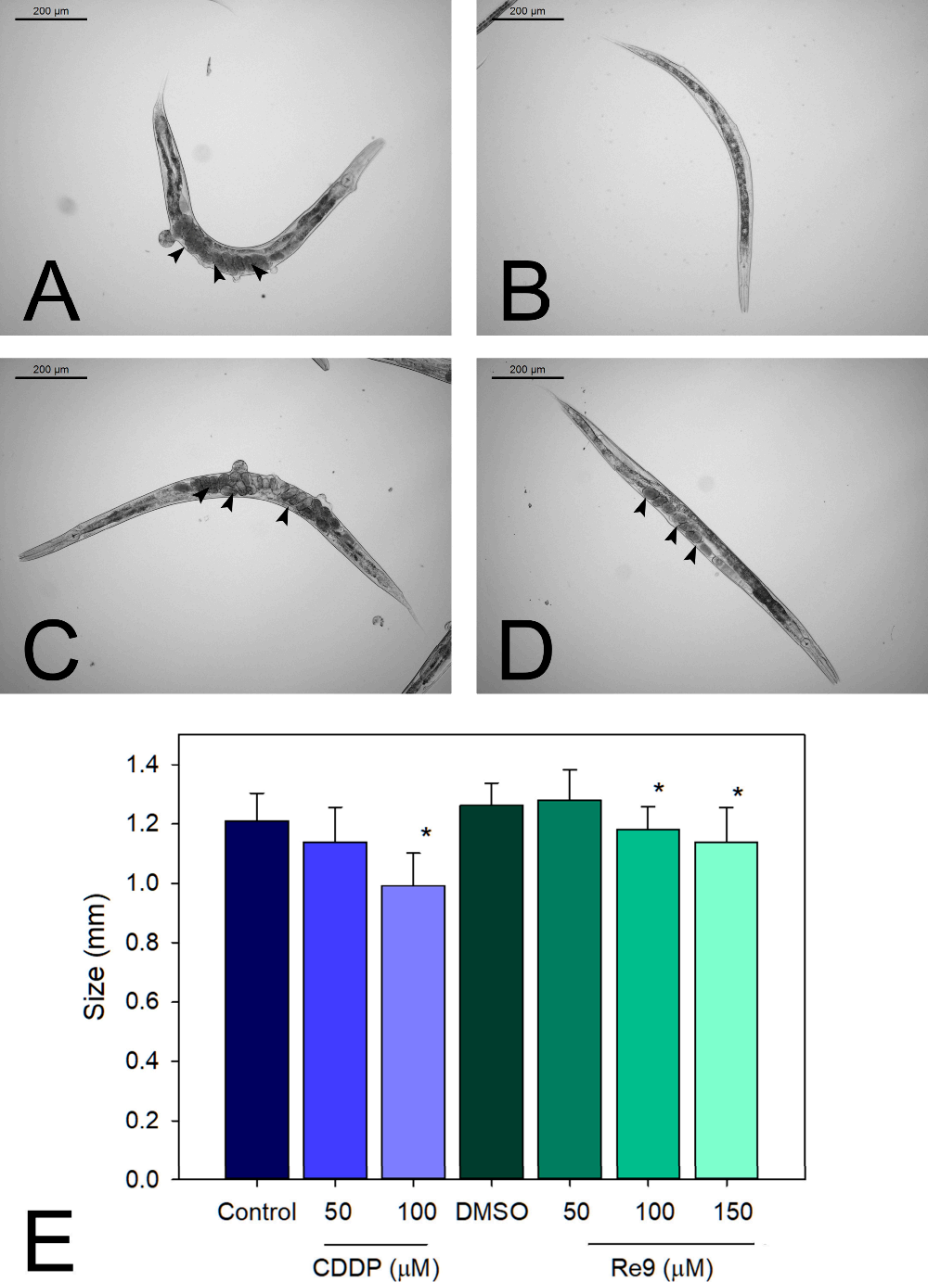
Effects of **Re9** and CDDP on *C. elegans* strain MT2124 size. (A–D) Representative images of *C. elegans* MT2124-treated with the complexes for 72 h. (A) Water-treated nematode, arrowheads mark the oocytes and eggs. (B) Cisplatin (100 μM)-treated animal. (C) DMSO-treated nematode. (D) **Re9** (100 μM)-treated worm. Scale bar 200 μm. (E) Size measurement. Two independent assays were performed with *n* ≥ 20. Data is represented as average ± SD * significant at *p* ≤ 0.05 by the ANOVA test.

The toxicity effects of **Re9** and cisplatin were evaluated by measuring the size and developmental stage of the MT2124 animals exposed to different concentrations of the metal complexes for 72 h. Cisplatin was more toxic than **Re9**; at 100 μM, it reduced the animal size by 18%, whereas under the same conditions, the size of the **Re9**-treated animals was reduced by only 2% (Figure 15E). Moreover, cisplatin hindered the nematode’s development; ordinally, *C. elegans* maintained at 20 °C for 72 h grows from the L1 stage to the young adult stage. At this stage, the gonads are already formed, and there are fertilized oocytes and eggs, as shown in the representative images of Figure 15A,C,D, corresponding to control animals and animals treated with 100 and 150 μM of **Re9**, respectively. Meanwhile, cisplatin-treated animals lacked mature gonads, which is indicative of nematodes at the L4 stage (Figure 15B). García-Rodríguez and coauthors^72^ reported similar results when wild-type larvae were exposed to increasing doses of cisplatin for 48 h; overall, at 100 μM, the animal size was reduced by a 75%. The development stage for control animals at 48 h was of young adults; meanwhile, the nematodes exposed to 100 μM of the compound were in the L2 stage. When L4 animals were treated with cisplatin (100 μM), their progeny was also affected and had only a 10% the expected brood.^72^ Therefore, **Re9** is as effective as cisplatin on reducing the tumor growth in both *C. elegans* tumoral strains Jk1466 and MT2124, with the advantage of being less toxic and more selective, as it did not hinder the nematode development and progeny, as it happened with cisplatin.

### **Re9** Involvement on ROS Formation *In Vivo*

The capacity of **Re9** to increase reactive oxygen species in the *C. elegans* strain MT2124 was studied in order to dilucidate the underlying mechanism of action of the antitumoral effect. The fluorescent probe used, H_2_DCFDA, is a fluorogenic dye that detects hydrogen peroxide, hydroxyl radicals and peroxynitrites. However, the probe does not detect superoxide anions; thus, the nematodes were also stained with dihydroethidium (DHE), a selective probe for superoxide anions.

**Figure 16:**
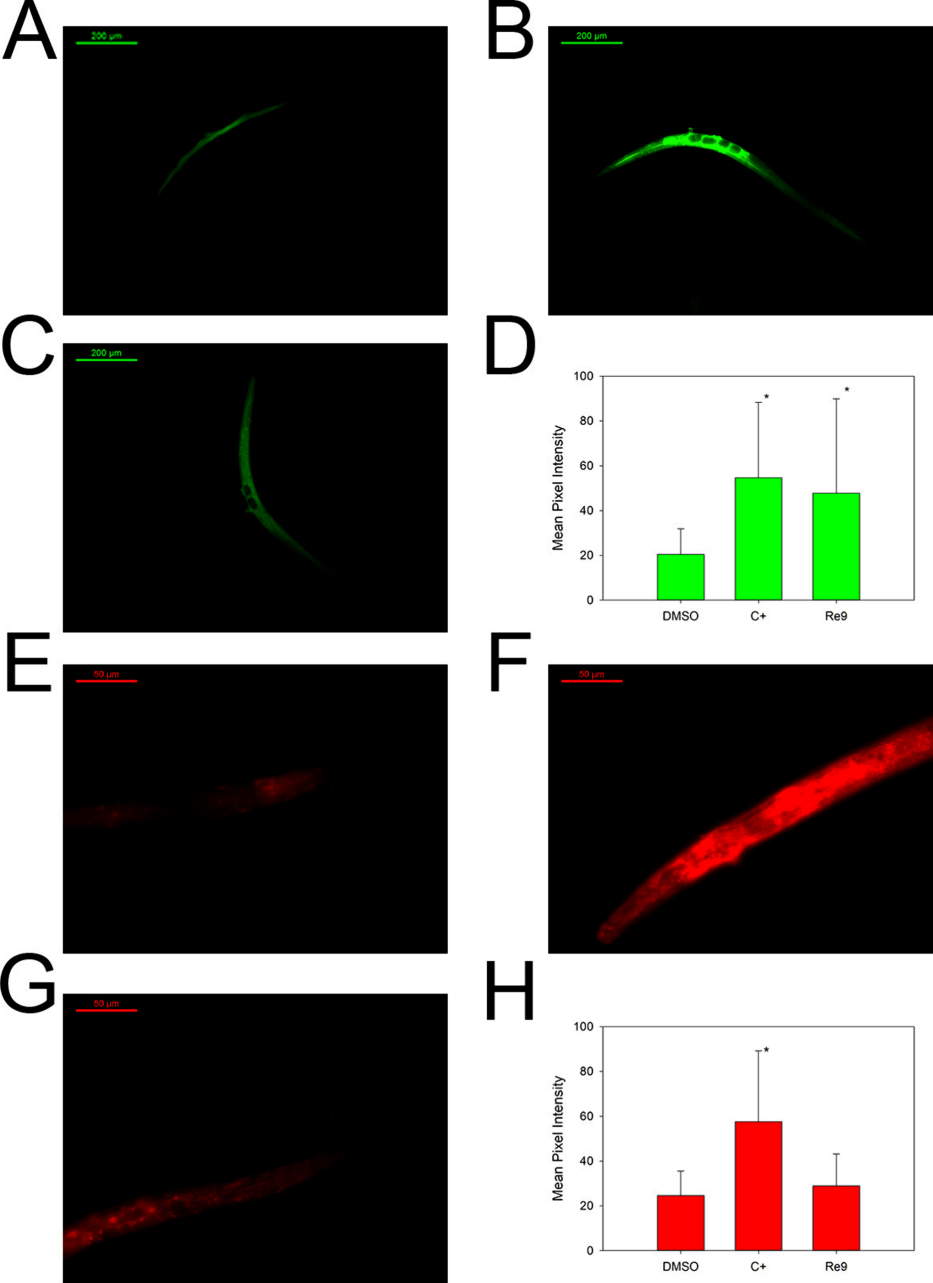
ROS measurements *in vivo*. (A–C) Representative images of MT2124 nematodes stained with DCFH-DA. (A) DMSO-treated nematode. (B) Juglone (20 μM)-treated animal. (C) **Re9** (150 μM)-treated animal. Scale bar: 200 μm. (D) ROS measurement. Two independent assays were performed with *n* ≥ 20. Data is represented as average ± SD * significant at *p* ≤ 0.05 by the ANOVA test. (E–H) Representative images of MT2124 nematodes stained with DHE. (E) DMSO-treated nematode. (F) Paraquat (200 μM)-treated animal. (G) **Re9** (150 μM)-treated animal. Scale bar: 200 μm. (H) Superoxide measurement. Two independent assays were performed with *n* ≥ 20. Data is represented as average ± SD * significant at *p* ≤ 0.05 by the ANOVA test.

Up on 20 h of exposure to 150 μM **Re9**, the level of total ROS inside the nematodes was increased 2-fold (Figure 16C,D) in comparison with the control treated animals (Figure 16A). In contrast, the compound did not generate superoxide anions in the animals (Figure 16G,H). Normally, cancer cells are more sensible to extracellular H_2_O_2_ because the level of their antioxidants enzymes is usually lower than in healthy cells. Thus, when exposed to a high influx of ROS, the cancerous cells lack detox mechanisms to remove them. The accumulation of H_2_O_2_ inside the cell may suppress the tumor growth by activating pro-apoptotic signals that may lead to cell death.^73,74^ Therefore, it is likely that **Re9** is able to reduce the cell proliferation in *C. elegans* by unbalancing the redox status of the tumoral cells.

## Conclusions

We have synthesized nine new anticancer Re(I) agents of the type *fac*-[Re(CO)_3_(N^N)L]^0/+^**Re1**– **Re9** to explore the effect of the different N^N ligands derived of benzimidazole and the monodentate chloride or pyridine derivative ligands on their optical properties and biological activity. In addition, the ester group in the N^N ligand allow further intended functionalization. The anticancer activity of the investigated Re(I) complexes was determined against cervix (HeLa), ovarian (A2780) cancer cells, and BGM as the model cell line for normal cells. The nature of the monodentate ligand **L** strongly impacted the biological properties, exhibiting the cationic complexes incorporating 4-NMe_2_py as the axial ligand, **Re7**–**Re9**, the best performance. Compound **Re9** exhibited potent anticancer activity *in vitro* against a panel of cancer cell lines and 3D HeLa spheroids, and *in vivo* in two *C. elegans* tumoral strains, JK1466 and MT2124, representatives of a broad diversity of human cancers. Biological investigations, employing confocal microscopy and flow cytometry techniques, provided compelling evidence of **Re9**’s specific affinity for accumulating in the mitochondria of living cancer cells. The specific targeting of **Re9** to mitochondria suggests its potential role in disrupting mitochondrial function, inducing cell death mechanisms. Additionally, the compound was able to reduce the germline cell proliferation in the strain JK1466 by a 34% at 150 μM; meanwhile, at the same concentration, **Re9** was able to revert the multivulva phenotype of the strain MT2124 by a 68.7%. Experiments with cisplatin at the same concentration were not possible as it was toxic for the animals. When the nematodes were exposed to 100 μM of cisplatin, the compound reduced their size and hampered their normal development. Therefore, the new anticancer compound **Re9** was as effective as cisplatin and had better selectivity and lower toxicity toward healthy cells. Mechanistically, **Re9** was found to increase the generation of reactive oxygen species (ROS) both *in vivo* and *in vitro*. This enhanced ROS production may be attributed to the activation of pyroptotic pathways or redox imbalance within the tumoral cells, ultimately leading to a reduction in cell proliferation.

## Experimental Section

### Materials and Instrumentation

4-Chloro-3-nitrobenzoic acid, butylamine, zinc in powder, ammonium formate, 2-pyridinecarboxaldehyde, 2-quinolinecarboxaldehyde, benzo[*b*]thiophene-2-carboxaldehyde, Re(CO)_5_Cl, AgCF_3_SO_3_, potassium hexafluorophosphate, pyridine and 4-*N,N*-(dimethylamino)pyridine, and propidium iodide were obtained from Sigma-Aldrich (Madrid, Spain) and used without further purification. The purity of ≥95% of the synthesized complexes used for biological evaluation was determined by elemental analysis and RP-HPLC. The ^1^H and ^13C^{^1^H} NMR spectra were recorded on a Bruker AC 300E, Bruker AV 400, or Bruker AV 600 NMR spectrometer, and chemical shifts were determined by reference to the residual ^1^H and ^13^C{^1^H} solvent peaks. The C, H, N, and S analyses were performed with a Carlo Erba model EA 1108 microanalyzer, with an EAGER 200 software. IR spectra were recorded in a Jasco FT/IR-4600 spectrometer with an ATR-PRO ONE system. The HPLC/MS spectra were performed in an Agilent LC/Q-TOF 6546. The column was a Zorbax Eclipse Plus C18, 2.1 × 50 mm, 1.8 micras. The mobile phase was A (water +0.05% acetic acid) and B (acetonitrile) with a gradient of 2–95% of B. The flow was 0.4 mL/min. The detection wavelength was 400 nm. The samples were dissolved in ACN. The MS spectra show an isotopic distribution of the heaviest set of peaks matched very closely to that calculated for the formulation of the complex cation in every case. The purity of ≥95% of the synthesized complexes used for biological evaluation was determined by RP-HPLC.

### Synthesis Procedures

#### Synthesis of N^N ligands (**L1**–**L3**)

The preparation of ligands **L1**–**L3** was carried out as previously reported.^48,52^

#### Synthesis of Re(I) complexes (**Re1**–**Re9**)

All reactions were carried out under a nitrogen atmosphere. Complexes **Re1**–**Re3** were prepared according to the bibliography.^55^ A solution of the corresponding N^N ligand (0.25 mmol) and Re(CO)_5_Cl (0.25 mmol) were stirred at 110 °C in 10 mL of toluene for 4 h. The mixture was cooled, filtered, and washed with diethyl ether. A yellow-orange solid was obtained in good yields.

##### (**Re1**)

Yellow solid. Isolated yield 50%. ^1^H NMR (600 MHz, CDCl_3_) δ 9.26 (dd, *J* = 5.5, 1.0 Hz, 1H), 8.82 (d, *J* = 1.1 Hz, 1H), 8.26 (dd, *J* = 8.7, 1.1 Hz, 1H), 8.17 (ddd, *J* = 8.1, 7.5, 1.0 Hz, 1H), 8.12 (dd, *J* = 8.1, 0.9 Hz, 1H), 7.61 (ddd, *J* = 7.5, 5.5, 0.9 Hz, 1H), 7.58 (d, *J* = 8.7 Hz, 1H), 4.70–4.57 (m, 2H), 4.01 (s, 3H), 2.03 (m, 2H), 1.59 (m, 2H), 1.06 (t, *J* = 7.4 Hz, 3H). ^13^C NMR (151 MHz, CDCl_3_) δ 197.2, 196.8, 188.7, 166.4, 155.4, 153.4, 147.3, 140.1, 139.4, 138.6, 128.3, 128.0, 127.4, 123.6, 122.6, 110.8, 52.8, 46.4, 31.9, 20.4, 13.8. Mass ESI-MS (pos. ion mode, DMSO): calc.: [M-Cl]^+^ = 580.0882 *m*/*z*; exp.: 580.0854 *m*/*z*. Anal. calc. for C_21_H_19_ClN_3_O_5_Re: %C, 41.01; %H, 3.11; %N, 6.83. Found: %C, 40.70; %H, 3.08; %N, 6.75.

##### (**Re2**)

Yellow solid. Isolated yield 71%. ^1^H NMR (400 MHz, CDCl_3_) δ 9.09 (dd, *J* = 8.8, 0.7 Hz, 1H), 8.95 (d, *J* = 1.1 Hz, 1H), 8.60 (d, *J* = 8.4 Hz, 1H), 8.28 (dd, *J* = 8.7, 1.1 Hz, 1H), 8.18 (d, *J* = 8.4 Hz, 1H), 8.07 (ddd, *J* = 8.8, 6.9, 1.3 Hz, 1H), 7.98 (dd, *J* = 8.0, 1.3 Hz, 1H), 7.81 (ddd, *J* = 8.0, 6.9, 0.7 Hz, 1H), 7.61 (d, *J* = 8.7 Hz, 1H), 4.79–4.61 (m, 2H), 4.03 (s, 3H), 2.06 (m, 2H), 1.59 (m, 2H), 1.06 (t, *J* = 7.3 Hz, 3H). ^13^C NMR (101 MHz, CDCl_3_) δ 197.3, 196.1, 188.9, 166.4, 155.5, 149.3, 149.1, 140.9, 140.7, 138.7, 133.7, 131.3, 130.1, 128.8, 128.6, 128.3, 127.9, 122.5, 119.0, 110.9, 52.9, 46.8, 32.2, 20.4, 13.8. Mass ESI-MS (pos. ion mode, DMSO): calc.: [M-Cl]^+^ = 630.1039 *m*/*z*; exp.: 630.1065 *m*/*z*. Anal. calc. for C_25_H_21_ClN_3_O_5_Re: %C, 45.15; %H, 3.18; %N, 6.32. Found: %C, 45.10; %H, 3.12; %N, 6.30.

##### (**Re3**)

Orange solid. Isolated yield 54%. ^1^H NMR (400 MHz, CDCl_3_) δ 8.91 (d, *J* = 1.0 Hz, 1H), 8.69 (dt, *J* = 8.4, 0.9 Hz, 1H), 8.30 (dd, *J* = 8.8, 1.0 Hz, 1H), 8.09 (dt, *J* = 8.1, 0.8 Hz, 1H), 7.88 (ddd, *J* = 8.4, 7.3, 0.8 Hz, 1H), 7.74 (ddd, *J* = 8.1, 7.3, 0.9 Hz, 1H), 7.62 (d, *J* = 8.8 Hz, 1H), 4.68 (m, 2H), 4.03 (s, 3H), 2.05 (m, 2H), 1.60 (m, 2H), 1.06 (t, *J* = 7.3 Hz, 3H). ^13^C NMR (101 MHz, CDCl_3_) δ 196.9, 195.9, 187.3, 166.2, 155.6, 150.5, 150.3, 140.9, 138.0, 133.0, 130.0, 129.3, 128.8, 128.5, 124.6, 122.6, 122.5, 111.2, 52.9, 47.1, 32.3, 20.5, 13.8. Mass ESI-MS (pos. ion mode, DMSO): calc.: [M-Cl]^+^ = 636.0603 *m*/*z*; exp.: 636.0584 *m*/*z*. Anal. calc. for C_23_H_19_ClN_3_O_5_ReS: %C, 41.16; %H, 2.85; %N, 6.26; %S, 4.78. Found: %C, 41.20; %H, 2.82; %N, 6.20; %S, 4.71.

Complexes **Re4**–**Re9** were prepared according to the bibliography.^56^ A solution of the corresponding **Re1**–**Re3** complex (0.15 mmol) and AgCF_3_SO_3_ (0.15 mmol) was stirred in 50 mL of acetonitrile at 80 °C for 24 h. After removing off the AgCl precipitate, the remaining solution was evaporated to obtain orange solid, which was used without further purification. The solid was dissolved in dry THF:CH_3_OH (3:1). Then, pyridine or 4-*N*,*N*-dimethylaminepyridine (0.15 mmol) was added and the mixture was stirred at 60 °C for 12 h. After the reaction time, KPF_6_ (0.75 mmol) was added, and the mixture was stirred for 1 h. The crude was evaporated, and the resulting solid was filtered and washed with water and hexane. The solid was purified by aluminum oxide column chromatography using CH_2_Cl_2_:CH_3_CN (8:2) as eluent. A yellow-orange solid was obtained in low or good yields.

##### (**Re4**)

Yellow solid. Isolated yield 32%. ^1^H NMR (600 MHz, CD_3_CN) δ 9.39 (dd, *J* = 5.4, 1.0 Hz, 1H), 8.81 (d, *J* = 1.1 Hz, 1H), 8.35 (ddd, *J* = 8.8, 8.1, 1.0 Hz, 1H), 8.29 (dd, *J* = 8.7, 1.5 Hz, 1H), 8.28 (dd, *J* = 8.3, 0.9 Hz, 1H), 8.18 (dd, *J* = 6.6, 1.5 Hz, 2H), 7.93 (dd, *J* = 8.6, 0.5 Hz, 1H), 7.86 (ddd, *J* = 7.5, 5.4, 1.1 Hz, 1H), 7.80 (tt, *J* = 7.7, 1.5 Hz, 1H), 7.24–7.20 (dd, J = 7.7, 6.6 Hz, 2H), 4.66 (m, 2H), 4.00 (s, 3H), 1.87 (m, 2H), 1.39 (m, 2H), 0.90 (t, *J* = 7.3 Hz, 3H). ^13^C NMR (151 MHz, CD_3_CN) δ 197.3, 196.7, 191.8, 167.1, 156.7, 156.1, 153.2, 147.7, 142.7, 140.7, 140.2, 140.2, 130.2, 129.6, 128.3, 127.6, 126.8, 121.1, 113.8, 53.3, 47.4, 31.9, 20.5, 13.9. Mass ESI-MS (pos. ion mode, DMSO): calc.: [M-PF_6_]^+^ = 659.1304 *m*/*z*; exp.: 659.1325 *m*/*z*. Anal. calc. for C_26_H_24_F_6_N_4_O_5_PRe: %C, 38.86; %H, 3.01; %N, 6.97. Found: %C, 38.95; %H, 3.10; %N, 6.86.

##### (**Re5**)

Yellow solid. Isolated yield 16%. ^1^H NMR (600 MHz, CD_3_CN) δ 9.05 (dd, *J* = 8.8, 0.8 Hz, 1H), 8.94 (d, *J* = 1.4 Hz, 1H), 8.89 (d, *J* = 8.5 Hz, 1H), 8.33 (dd, *J* = 8.8, 1.4 Hz, 1H), 8.28 (m, 3H), 8.02 (ddd, *J* = 8.0, 6.9, 0.8 Hz, 1H), 7.97 (d, *J* = 8.8 Hz, 1H), 7.72 (m, 3H), 7.06 (m, 2H), 4.73 (m, 2H), 4.02 (s, 3H), 1.84 (m, 2H), 1.33 (m, 2H), 0.88 (t, *J* = 7.4 Hz, 3H). ^13^C NMR (151 MHz, CD_3_CN) δ 197.3, 196.2, 192.1, 167.1, 157.0, 153.0, 150.4, 149.3, 144.1, 140.8, 140.8, 140.4, 135.7, 131.5, 130.9, 130.8, 130.6, 129.9, 128.6, 127.6, 121.7, 121.3, 114.1, 53.4, 47.8, 32.3, 20.5, 13.9. Mass ESI-MS (pos. ion mode, DMSO): calc.: [M-PF_6_]^+^ = 709.1461 *m*/*z*; exp.: 709.1482 *m*/*z*. Anal. calc. for C_30_H_26_F_6_N_4_O_5_PRe %C, 42.21; %H, 3.07; %N, 6.56. Found: %C, 42.26; %H, 3.12; %N, 6.38.

##### (**Re6**)

Orange solid. Isolated yield 27%. ^1^H NMR (600 MHz, CDCl_3_) δ 8.90 (d, *J* = 1.0 Hz, 1H), 8.68 (dd, *J* = 8.5, 0.8 Hz, 1H), 8.37 (dd, *J* = 8.6, 1.0 Hz, 1H), 8.26 (d, *J* = 8.2, 0.9 Hz, 1H), 8.02 (dd, *J* = 6.7, 1.5 Hz, 2H), 7.98 (ddd, *J* = 8.5, 7.2, 0.9 Hz, 1H), 7.84 (ddd, *J* = 8.2, 7.2, 0.8 Hz, 1H), 7.78 (d, *J* = 8.6 Hz, 1H), 7.72 (tt, *J* = 7.8, 1.5 Hz, 1H), 7.20 (dd, *J* = 7.8, 6.7 Hz, 2H), 5.00–4.75 (m, 2H), 4.06 (s, 3H), 2.00 (m, 2H), 1.55 (m, 2H), 1.02 (t, *J* = 7.4 Hz, 3H). ^13^C NMR (151 MHz, CDCl_3_) δ 195.9, 194.8, 189.2, 166.0, 158.3, 151.8, 151.0, 149.5, 140.2, 140.1, 138.7, 134.5, 130.7, 129.7, 129.6, 128.9, 127.3, 124.2, 122.6, 120.5, 113.1, 53.1, 47.7, 32.1, 20.5, 13.8. Mass ESI-MS (pos. ion mode, DMSO): calc.: [M-PF_6_]^+^ = 715.1025 *m*/*z*; exp.: 715.1045 *m*/*z*. Anal. calc. for C_28_H_24_F_6_N_4_O_5_PReS: %C, 39.12; %H, 2.81; %N, 6.52; %S, 3.73. Found: %C, 39.02; %H, 2.85; %N, 6.49; %S, 3,79.

##### (**Re7**)

Yellow solid. Isolated yield 59%. ^1^H NMR (600 MHz, CD_3_CN) δ 9.35 (d, *J* = 5.5, 0.9 Hz, 1H), 8.77 (d, *J* = 1.4 Hz, 1H), 8.35 (ddd, *J* = 8.8, 7.9, 1.6 Hz, 1H), 8.31 (dt, *J* = 7.8, 0.9 Hz, 1H), 8.29 (dd, *J* = 8.8, 1.4 Hz, 1H), 7.92 (d, *J* = 8.8 Hz, 1H), 7.83 (ddd, *J* = 7.2, 5.5, 1.4 Hz, 1H), 7.53 (d, *J* = 7.4 Hz, 2H), 6.24 (d, *J* = 7.4 Hz, 2H), 4.69 (td, *J* = 7.8, 2.5 Hz, 2H), 3.99 (s, 3H), 2.85 (s, 6H), 1.85 (m, 2H), 1.34 (m, 2H), 0.88 (t, *J* = 7.4 Hz, 3H). ^13^C NMR (151 MHz, CD_3_CN) δ 197.8, 197.2, 192.2, 167.0, 156.6, 155.9, 155.6, 151.5, 147.6, 142.5, 140.3, 140.1, 129.9, 129.5, 128.3, 126.7, 121.1, 113.8, 108.9, 53.3, 47.4, 39.4, 32.0, 20.5, 13.9. Mass ESI-MS (pos. ion mode, DMSO): calc.: [M-PF_6_]^+^ = 702.1726 *m*/*z*; exp.: 702.1748 *m*/*z*. Anal. calc. for C_28_H_29_F_6_N_5_O_5_PRe: %C, 39.72; %H, 3.45; %N, 8.27. Found: %C, 39.84; %H, 3.54; %N, 8.29.

##### (**Re8**)

Orange solid. Isolated yield 48%. ^1^H NMR (400 MHz, DMSO-*d*_6_) δ 9.13 (dd, *J* = 8.6, 0.5 Hz, 1H), 8.93 (dd, *J* = 8.5, 0.5 Hz, 1H), 8.79 (d, *J* = 0.5 Hz, 1H), 8.61 (d, *J* = 8.6 Hz, 1H), 8.45 (dd, *J* = 8.0, 1.0 Hz, 1H), 8.35 (ddd, *J* = 8.5, 7.2, 1.0 Hz, 1H), 8.30 (m, 2H), 8.07 (ddd, *J* = 8.0, 7.2, 0.8 Hz, 1H), 6.98 (d, *J* = 7.6 Hz, 2H), 6.30 (d, *J* = 7.6 Hz, 2H), 4.98 (t, *J* = 7.4 Hz, 2H), 3.99 (s, 3H), 2.78 (s, 6H), 1.81–1.64 (m, 2H), 1.26–1.07 (m, 2H), 0.75 (t, *J* = 7.4 Hz, 3H). ^13^C NMR (151 MHz, DMSO) δ 196.9, 195.7, 191.7, 165.6, 155.8, 154.1, 149.6, 149.1, 147.5, 143.3, 139.1, 134.8, 130.4, 129.9, 129.4, 128.8, 128.0, 127.3, 121.2, 119.3, 113.9, 108.2, 52.8, 46.3, 38.5, 31.5, 19.1, 13.5. Mass ESI-MS (pos. ion mode, DMSO): calc.: [M-PF_6_]^+^ = 752.1883 *m*/*z*; exp.: 752.1904 *m*/*z*. Anal. calc. for C_32_H_31_F_6_N_5_O_5_PRe: %C, 42.86; %H, 3.48; %N, 7.81. Found: %C, 42.86; %H, 3.58; %N, 7.87.

##### (**Re9**)

Yellow solid. Isolated yield 36%. ^1^H NMR (600 MHz, CD_3_CN) δ 8.88 (d, *J* = 1.1 Hz, 1H), 8.70 (dd, *J* = 8.4, 0.9 Hz, 1H), 8.38 (dt, *J* = 8.3, 1.0 Hz, 1H), 8.34 (dd, *J* = 8.8, 1.1 Hz, 1H), 8.06 (ddd, *J* = 8.4, 7.2, 1.0 Hz, 1H), 7.97 (d, *J* = 8.8 Hz, 1H), 7.89 (ddd, *J* = 8.3, 7.2, 0.9 Hz, 1H), 7.42 (d, *J* = 7.4 Hz, 2H), 6.17 (d, *J* = 7.4 Hz, 2H), 4.72 (m, 2H), 4.01 (s, 3H), 2.81 (s, 6H), 2.13 (m, 2H), 1.46–1.28 (m, 2H), 0.91 (t, *J* = 7.3 Hz, 3H). ^13^C NMR (151 MHz, CD_3_CN) δ 197.24, 196.8, 190.9, 166.9, 159.8, 155.6, 152.5, 151.7, 150.3, 141.0, 139.5, 135.1, 131.6, 130.5, 130.2, 128.9, 125.2, 123.6, 121.2, 114.2, 108.9, 53.4, 48.2, 39.3, 32.2, 20.7, 13.9. Mass ESI-MS (pos. ion mode, DMSO): calc.: [M-PF_6_]^+^ = 758.1447 *m*/*z*; exp.: 758.1468 *m*/*z*. Anal. calc. for C_30_H_29_F_6_N_5_O_5_PReS: %C, 39.91; %H, 3.24; %N, 7.76; %S, 3.55. Found: %C, 39.83; %H, 3.25; %N, 7.67; %S, 3.59.

### X-ray Crystal Structure Analysis

A suitable crystal of **Re3** was grown upon slow solvent evaporation from an NMR tube of a solution of **Re3** in CDCl_3_, whereas tiny needle crystals of **Re3**·CHCl_3_ were grown from CHCl_3_/hexane. Crystals of **Re8** were grown from acetonitrile/hexane. Details of the X-ray structure determinations and refinement parameters for the compound are given in Tables S2 and S3 in the Supporting Information. Crystals were mounted on glass fibers and transferred to the cold gas stream of the diffractometer Bruker Smart APEX. Data were recorded with Mo *K*α radiation (λ = 0.71073 Å) in ω scan mode. The structure was solved by direct methods; refinement was done by full-matrix least-squares on *F*^2^ using the SHELXL program suite^75,76^; empirical (multiscan) absorption correction with SADABS (Bruker). Graphics were drawn with DIAMOND.^77^ CCDC reference numbers are 2282513 for **Re3**, 2325369 for **Re3**·CHCl_3_ and 2282514 for **Re8**. Special features: the butyl chain in **Re8** is disordered over two positions with 58 and 42% occupancy for the A and B labeled atoms. Further, the PF_6_ anion is rotationally disordered. The structure of **Re8** also contains two partially occupied CH_2_Cl_2_ solvent molecules (79 and 55% occupancy) with large temperature factors and unaccounted solvent residues possibly from a shared position of CH_2_Cl_2_ with a hexane cosolvent molecule. These unaccounted solvent residues give rise to solvent accessible voids of 106 Å^3^ in the structure of **Re8**. An image of the molecule of **Re8** with the butyl chain disorder and the two CH_2_Cl_2_ solvent molecules is shown in Table S3b

### Photophysical Characterization

UV/vis spectroscopy was carried out on a PerkinElmer Lambda 750 S spectrometer with the operating software. Solutions of all complexes were prepared in acetonitrile and water (1% DMSO) at 10 μM. Emission spectra were obtained with a Horiba Jobin Yvon Fluorolog 3–22 modular spectrofluorometer with a 450 W xenon lamp. Measurements were performed in a right-angled configuration using 10 mm quartz fluorescence cells for solutions at 298 K. Emission quantum yields (Φ) were measured using a Hamamatsu C11347 absolute PL quantum yield spectrometer; the estimated uncertainty is ±10% or better. For quantum yields measurements, solutions of all complexes were prepared in acetonitrile and previously degassed by bubbling argon for 20 min.

### Stability in Solution and Cell Culture Medium

The stability of complexes in DMSO and cell culture medium was evaluated by UV/vis spectra at *t* = 0 and after 48 h at 37 °C. The solutions were prepared in DMSO or RPMI (5% DMSO) at 10 μM.

### Biological Studies

#### Cell Culture and Re Complex Stock Solutions

Human ovarian carcinoma cell lines (A2780) were cultured in RPMI-1640 medium, while the human cervix adenocarcinoma cell line (HeLa), tumor breast cancer cell line, and nontumorigenic buffalo green monkey cells (BGM) were cultured in DMEM and EMEM (containing nonessential amino acids) medium. The cell culture media were supplemented with 10% fetal bovine serum (FBS), 1% l-glutamine, and 1% penicillin/streptomycin. The cells were maintained in a humidified incubator at 37 °C with a 5% CO_2_ atmosphere and subcultured 2–3 times a week, each with an appropriate density for its specific cell line. Prior to the experiments, the cell lines were confirmed to be free from mycoplasma contamination using Hoechst DNA staining standard procedures. During cell-based assays, the maximum amount of dimethyl sulfoxide (DMSO) added as a solvent for treatment was limited to 0.4% (v/v) to avoid any potential vehicle-induced toxicity to the cells.

#### Antiproliferative Activity

Cells were cultured in 96-well plates and allowed to reach confluence. The tested compounds were dissolved in DMSO at a maximum concentration of 0.4% (v/v) and immediately diluted with fresh media. The cells were then incubated with varying concentrations of the **Re1**–**Re9** for 48 h at 37 °C. After the incubation period, a 50 μL aliquot of MTT solution (1 mg/mL) was added to each well, and the plates were further incubated for 4 h. The culture medium was carefully removed, and DMSO (50 μL per well) was added and incubated for 5 min with shaking. The absorbance at 570 nm was measured using a microplate reader (FLUOstar Omega).

#### Cytotoxicity Evaluation on 3D Multicellular Spheroids

To generate HeLa multicellular tumor spheroids (MCTS), 96-well Corning microplates with an ultralow attachment surface coating were utilized. The process involved preparing a single suspension of HeLa cells at a density of 5 × 10^3^ cells per well in complete DMEM medium, which was then dispensed into the wells. The plates were covered and placed in an incubator with a temperature of 37 °C and a 5% CO_2_ atmosphere. Within 3 days, uniform MCTS with a diameter of 200 μm were formed from the cell suspension and maintained under these conditions. On the first day of treatment, the MCTS were treated with **Re7**–**Re9** and cisplatin at their concentration of IC_50_. The media were changed every 3 days by replacing 50% of the existing media. The formation, integrity, diameter, and volume of the MCTS were monitored over a span of 10 days using a DMi1 inverted phase contrast microscope (Leica Microsystems). The volumes of the MCTS were calculated using the equation *V* = 4/3π*r*^3^, where “*V*″ represents volume and “*r*″ represents the radius of the MCTS measured with ImageJ software.

#### ICP-MS Measurement

A2780 cells were seeded in 6-well plates at a density of 10^6^ cells per well in 1.8 mL of complete growth medium and incubated for 24 h prior to treatment. Subsequently, the cells were treated with 5 μM of the **Re7**–**Re9** and cisplatin for 2 h at 37 and 4 °C. After trypsinization, the A2780 cells were counted and further digested in 30% HNO_3_ at room temperature overnight. The amount of rhenium was determined using inductively coupled plasma mass spectrometry (ICP-MS). The assay was performed in three independent experiments (*n* = 2 per replicate).

#### Confocal Fluorescence Imaging

Fluorescence microscopy experiments were conducted using a STELLARIS 8 Leica Microsystems confocal microscope, which featured a 405 nm laser diode, an argon-ion laser, and a 488 nm laser. The microscope was equipped with a temperature and CO_2_ control system. HeLa cells were cultured on ibidi-plates until they reached confluence. Subsequently, imaging was performed at 37 °C with a 63× glycerol immersion objective. In colocalization studies, Mitotracker Green staining (100 nM in PBS; 30 min) was observed using the 488 nm laser, while the 405 nm laser diode was employed for **Re9** detection. Colocalization coefficients were determined using the JaCoP plugin in ImageJ software.

#### Scanning Electron Microscopy

A2780 cells were treated with CDDP and **Re9** for 24 h. Cells then were fixed with 4% PFA fix solution for 30 min and washed with PBS three times. Sample were dehydrated through a graded series of ethanol (30, 50, 70, 95, and 100%), dried by the tertiary butanol method, and then imaged with a SEM operating at 20.0 kV.

#### Cell Death Study

The annexin V/PI assay was performed following the instructions provided by the manufacturer (Roche). A2780 cells were seeded in 12-well plates at a density of 3 × 10^5^ cells/well and incubated overnight. The cells were treated with specific concentrations of compounds **Re9** and cisplatin (positive control) for a duration of 24 h. After the treatment, the cells were collected and stained with annexin V and PI, following the procedure mentioned earlier. The staining was carried out at room temperature for 15 min in the absence of light, and the samples were immediately analyzed using flow cytometry (FACSCalibur Beckton Dickinson) with an excitation wavelength of 488 nm. The absorbance at 488 nm of compound **Re9** was considered negligible. The data obtained from the assay were analyzed using FlowJo Software (TreeStar).

#### DNA Damage Induction

DNA damage was evaluated by flow cytometry in A2780 cells. In brief, cells were seeded in 12-well plates at 2 × 10^5^ cells/well and treated for 24 h with **Re9** (2.5 and 5 μM) and cisplatin (10 μM) as a positive control for DNA damage induction. Cells were then collected by trypsinization, washed with PBS, and fixed in 200 μL 0.2% PFA for 5 min. After fixation, cells were pelleted, suspended in a 3% FBS solution containing anti phospho-H2AX (ser139) FITC-conjugated monoclonal antibody (CR55T33, eBioscience) at a concentration of 0.6 μg/mL, and incubated for 2 h at room temperature in the dark. Analysis of stained cells was carried out using a Becton Dickinson FACSCalibur flow cytometer with 10,000 acquisitions per sample, registering the FL1-H channel (λ_exc_ = 488 nm). Two independent experiments, each with *n* = 2, were conducted.

#### Intracellular ROS Generation

To assess the ROS generation ability of the **Re9** in cancer cells, A2780 cells were allowed to attach to the cell surface of 12-well plates at a density of 3 × 10^5^ cells per well. **Re9** (at a concentration of 1.25 and 2.5 mM) were then added for a duration of 24 h under two different oxygen conditions: normoxia (21% O_2_) and hypoxia (2% O_2_). Subsequently, a staining solution containing dihydroethidium (DHE) at a concentration of 10 mM was loaded into each well and incubated for 30 min. Afterward, the staining solution was removed, and the fluorescence emitted was measured using a flow cytometer (Fortessa X20) using the 96-well plate adaptation and analyzed by FlowJo Software.

#### Mitochondrial Membrane Potential Assay

A2780 cells were cultured in 12-well plate at a concentration of 3 × 10^6^ cells/well and treated with **Re9** and antimycin A (positive control) for a duration of 24 h. After the treatment period, the cells were collected and resuspended in prewarmed PBS containing JC-1 (1 μM). The cell suspension was then incubated for 30 min at 37 °C. Following the incubation, the cells were washed twice with PBS and immediately analyzed using a flow cytometer (FACSCAlibur Beckton Dickinson). Fluorescence measurements were performed by detecting both the monomeric (emission at 530 ± 30 nm; green) and aggregated (emission at 585 ± 30 nm; red) forms of JC-1 upon excitation at 488 nm. For each sample, a total of 10,000 events were acquired during the analysis.

### *Caenorhabditis elegans* Strains and Maintenance

*Caenorhabditis elegans* strains JK1466 [*gld-1(q485)/dpy-5(e61) unc-13(e51)*] and MT2124 [*let-60(n1046)*] were kindly donated by the Caenorhabditis Genetic Center (CGC, Saint Paul, Minnesota, United States), which is funded by the “NIH Office of Research Infrastructure Programs” (P40 OD010440). The strains were maintained and cultured following the protocols established by Stiernagle.^78−80^ Synchronous cohorts of *C. elegans* prepared using the bleach method were used in all the assays.

### Re9 ingestion by *C. elegans*

The localization of **Re9** inside the animals was performed using wild-type L4 larvae treated with **Re9** (150 μM) or DMSO (0.4%) for 20 h at 20 °C. Then, the animals were visualized under fluorescent light using the I3 filter cube of a Leica DM 2500 LED microscope. Images were acquired at 40× and 100× magnification.

### Antitumoral Evaluation in *C. elegans* Strain JK1466

**Re9** effect on tumor size *in vivo* was measured following the protocol described by Ortega and coauthors.^51^ Briefly, L1 larvae of *C. elegans* strain JK1466 were treated with the compound in a concentration range between 0.1 to 150 μM in S basal medium supplemented with previously induced *E. coli* HT115 *gld-1* at 20 °C under orbital shaking; DMSO 0.4% was used as negative control. Tumor size was evaluated at the fourth day of adulthood using a bright-field microscope Leica DM 2500 LED microscope equipped with a Leica DFC550 camera (Leica Microsystems, Wetzlar, Germany). Images of the tumoral gonads were taken at 40×, and the size of the tumor was evaluated using the ImageJ software. Two independent assays were performed with *n* ≥ 20, and the statistical significance was estimated by the ANOVA test.

### Antitumoral Evaluation in *C. elegans* Strain MT2124

MT2124 (Muv phenotype) L1 larvae were incubated for 72 h at 20 °C in S basal medium with different concentrations of **Re9** (0.1–150 μM) supplemented with *E. coli* OP50. Antitumoral evaluation was performed following the protocol described by Medina and coauthors with modifications.^81^ Ten microliters of a suspension containing young adult nematodes were place in a microscope slide along with 10 μL of sodium azide to reduce its movement. Images of whole worms were taken at 10× magnification using the bright-field microscope. The animals were classified as multivulva (MT) if they had more than one vulva protruding from the left lateral side (Figure 12B) or wild-type if they only had one (WT). Then, the percentage of multivulva animals were calculated with eq 1.
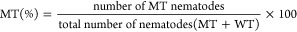
1

Additionally, the number of vulvas of the multivulva animals were counted. Two independent assays were performed with *n* ≥ 25 and the statistical significance was estimated by the ANOVA test.

Pseudovulvas morphology was visualized using acridine orange staining, following the protocol published by Ortega-Forte et al.^48^ MT2124 animals were treated with the 150 μM of **Re9** or DMSO for 72 h at 20 °C. Then, the nematodes were washed in M9 buffer and transferred to 5 mL of fresh M9 containing 100 μL of concentrated *E. coli* OP50 and 5 μg mL^–1^ of acridine orange and left to stain for 1 h at 20 °C under orbital shaking. After 1 h, the animals were washed with M9 three times. A Leica DM 2500 LED fluorescence microscope was used to acquire the images, using the I3 filter cube and 40× magnification.

### Size Measurements

Compounds toxicity was estimated using the parameters body length and development. Bright field images of animals treated with **Re9** (50, 100, and 150 μM), cisplatin (50 and 100 μM), and water (cisplatin control) or DMSO (**Re9** control) for 72 h at 20 °C were taken and analyzed using imageJ software.^82^ Body length was measured from the tip of the nose to the tail of each animal. The development stage was assigned considering the size, vulva and gonads formation, oocytes presence and the appearance of embryos.

### Measurement of Total ROS Generation *In Vivo*

Total ROS generated inside the nematodes were evaluated with the fluorescent probe 2′,7′-dichlorodihydrofluorescein diacetate (DCFH-DA), following the published protocol with modifications.^51^ L4 larvae were treated **Re9** (150 μM), DMSO (0.4%) or paraquat (methyl viologen 200 μM) at 20 °C; after 20 h, the supernatant was discarded and the animals were washed three times with M9. Then, the nematodes were incubated for 1 h in S medium containing 10 μM of DCFH-DA at 37 °C in the dark. The stained nematodes were washed again with M9 buffer and mounted onto glass slides containing 10 mM sodium azide to reduce their mobility. Images of fluorescence were taken at constant exposure times using the 10× magnification and the I3 filter cube. The analysis of the images was performed with ImageJ software using only the green channel. Two independent assays were performed with *n* ≥ 10 and the statistical significance was estimated by the ANOVA test.

### Measurement of Superoxide Anion Generation *In Vivo*

Superoxide anion production *in vivo* was measured with the specific fluorescent probe DHE (dihydroethidium) following the published protocol of Ortega-Forte et al.,^48^ with slight modifications. Briefly, synchronized wild-type L4 larvae were treated with **Re9** (150 μM), DMSO (0.4%) or paraquat (methyl viologen 200 μM) for 20 h at 20 °C. Then, the animals washed three times with PBS buffer and stained with 1 mL of a DHE solution (30 μM in PBS) for 1 h at 37 °C under orbital shaking. Following the staining procedure, the animals were visualized under fluorescent light using the N2.1 filter cube and the 40× lens. The analysis of the images was performed with ImageJ software using only the red channel. Two independent assays were performed with *n* ≥ 10, and the statistical significance was estimated by the ANOVA test.

## Supporting Information Available

Synthetic schemes of ligands L1-L3, nuclear magnetic resonance (NMR), IR spectroscopy, mass spectrometry, HPLC, X-ray diffraction, PD XRD measurements, photophysical properties, and photobiological and stability studies (DOCX)
X-ray crystallographic data of AMC_70_0m (CIF)
X-ray crystallographic data of MC_C3 (CIF)
Molecular formula strings and biological data (CSV)
X-ray crystallographic data of Re3_M4 (CIF)

## Supplementary Material

jm3c01869_si_001.docx

jm3c01869_si_002.cif

jm3c01869_si_005.cif

jm3c01869_si_006.csv

jm3c01869_si_007.cif

## Abbreviations Used

ACN: acetonitrile
CHCl_3_: chloroform
DMSO: dimethyl sulfoxide
IC_50_: concentration of the agent inhibiting cell growth by 50%
ICP-MS: inductively coupled plasma mass spectrometry
LLCT: ligand-to-ligand charge transfer
MLCT: metal-to-ligand charge transfer
