# Supplementary material for: Novel Re(I) Complexes as Potential Selective Theranostic Agents in Cancer Cells and In Vivo in Caenorhabditis elegans Tumoral Strains
Source: J Med Chem. 2024 Mar 7;67(10):7891–910. doi: 10.1021/acs.jmedchem.3c01869 (PMC11129195; doi:10.1021/acs.jmedchem.3c01869)
Supplement: Supplementary file 1 — jm3c01869_si_001.docx [file jm3c01869_si_001.docx]

**Supporting Information**

**Novel Re(I) complexes as potential selective theranostic agents in cancer cells and *in vivo* in *Caenorhabditis elegans* tumoral strains**

Alicia Marco,‡a Pezhman Ashoo,‡a Samanta Hernández-García,b Pedro Martínez-Rodríguez,b Natalia Cutillas,a Annette Vollrath,c Dustin Jordan,c Christoph Janiak,c Fernando Gandía-Herrero*,b and José Ruiz*a

*aDepartamento de Química Inorgánica, Universidad de Murcia, and Institute for Bio-Health Research of Murcia (IMIB-Arrixaca), E-30100 Murcia, Spain.*

*bDepartamento de Bioquímica y Biología Molecular A. Unidad Docente de Biología, Facultad de Veterinaria, Universidad de Murcia, E-30100, Murcia, Spain*

*cInstitut für Anorganische Chemie und Strukturchemie, Heinrich-Heine-Universität Düsseldorf, Universitätsstrasse 1, D-40225 Düsseldorf, Germany*

‡These authors contributed equally.

*Corresponding author’s e-mail address:

jruiz@um.es (J. Ruiz), [fgandia@um.es](mailto:fgandia@um.es) (F. Gandía-Herrero)

Table of Contents

[1. Synthetic schemes of ligands L1–L3 S2](#_Toc156547521)

[2. Nuclear Magnetic Resonance (NMR) S2](#_Toc156547522)

[3. IR spectroscopy S11](#_Toc156547523)

[4. Mass spectrometry S16](#_Toc156547524)

[5. High performance liquid chromatography (HPLC) analysis S19](#_Toc156547525)

[6. X-ray structure of complexes Re3, Re3·CHCl3 and Re8 S24](#_Toc156547526)

[7. Photophysical properties S35](#_Toc156547527)

[8. Stability studies S37](#_Toc156547528)

[9. Scanning electron microscopy S44](#_Toc156547529)

[10. Cell death study S45](#_Toc156547530)

[11. References S45](#_Toc156547531)

# 1. Synthetic schemes of ligands L1–L3

The N^N ligands (Scheme S1) were obtained as previously reported.1,2

**Scheme S1.** Synthesis of N^N ligands.

# 2. Nuclear Magnetic Resonance (NMR)

**Figure S1.** 1H NMR spectrum of **Re1**, 600 MHz, CDCl3.

**Figure S2.** 13C NMR spectrum of **Re1**, 151 MHz, CDCl3.

**Figure S3.** 1H NMR spectrum of **Re2**, 400 MHz, CDCl3.

**Figure S4.** 13C NMR spectrum of **Re2**, 101 MHz, CDCl3.

**Figure S5.** 1H NMR spectrum of **Re3**, 400 MHz, CDCl3.

**Figure S6.** 13C NMR spectrum of **Re3**, 101 MHz, CDCl3.

**Figure S7.** 1H NMR spectrum of **Re4**, 600 MHz, CD3CN.

**Figure S8.** 13C NMR spectrum of **Re4**, 151 MHz, CD3CN.

**Figure S9.** 1H NMR spectrum of **Re5**, 600 MHz, CD3CN.

**Figure S10.** 13C NMR spectrum of **Re5**, 151 MHz, CD3CN.

**Figure S11.** 1H NMR spectrum of **Re6**, 600 MHz, CDCl3.

**Figure S12.** 13C NMR spectrum of **Re6**, 151 MHz, CDCl3.

**Figure S13.** 1H NMR spectrum of **Re7**, 600 MHz, CD3CN.

**Figure S14.** 13C NMR spectrum of **Re7**, 151 MHz, CD3CN.

**Figure S15.** 1H NMR spectrum of **Re8**, 400 MHz, DMSO-*d6*.

**Figure S16.** 13C NMR spectrum of **Re8**, 151 MHz, DMSO-*d6*.

**Figure S17.** 1H NMR spectrum of **Re9**, 400 MHz, DMSO-*d6*.

**Figure S18.** 13C NMR spectrum of **Re9**, 151 MHz, CD3CN.

# 3. IR spectroscopy


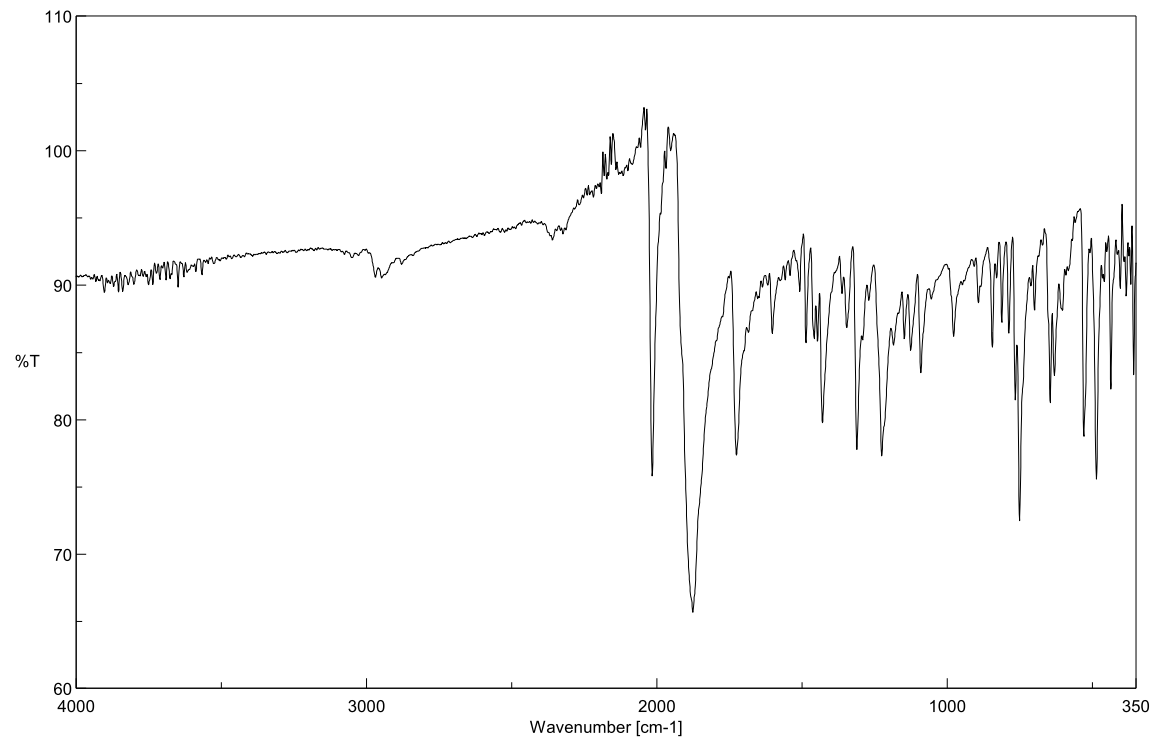


**Figure S19.** IR spectrum of complex **Re1**.


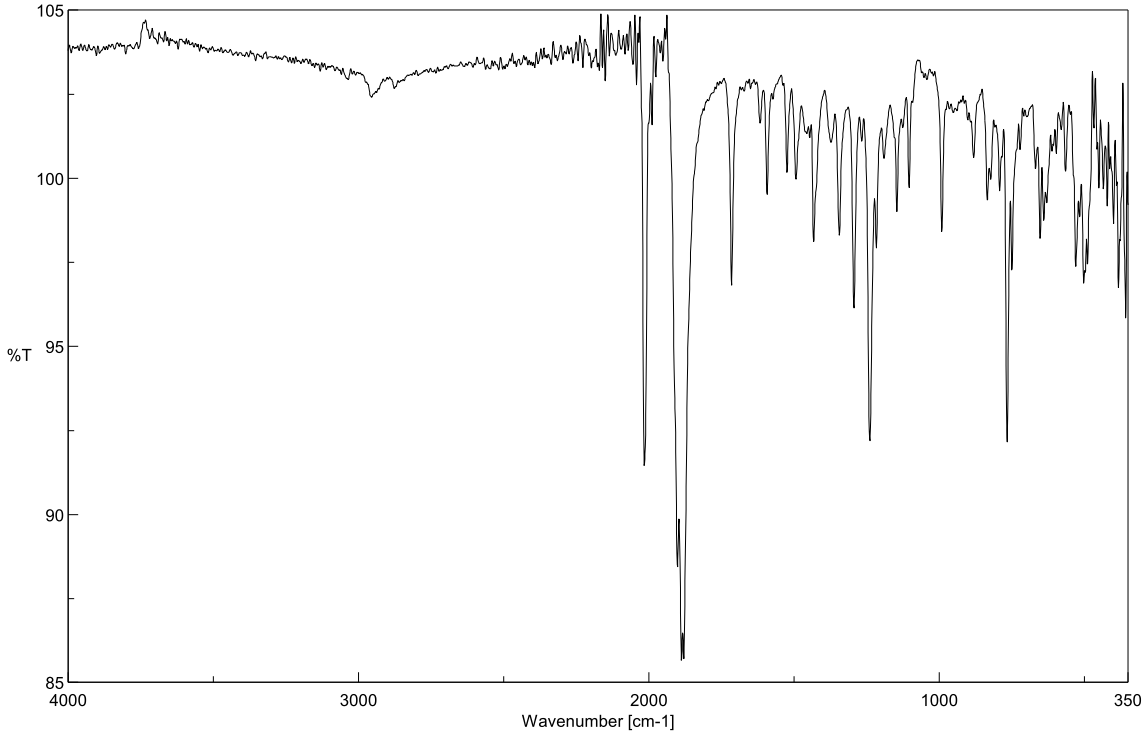


**Figure S20.** IR spectrum of complex **Re2**.


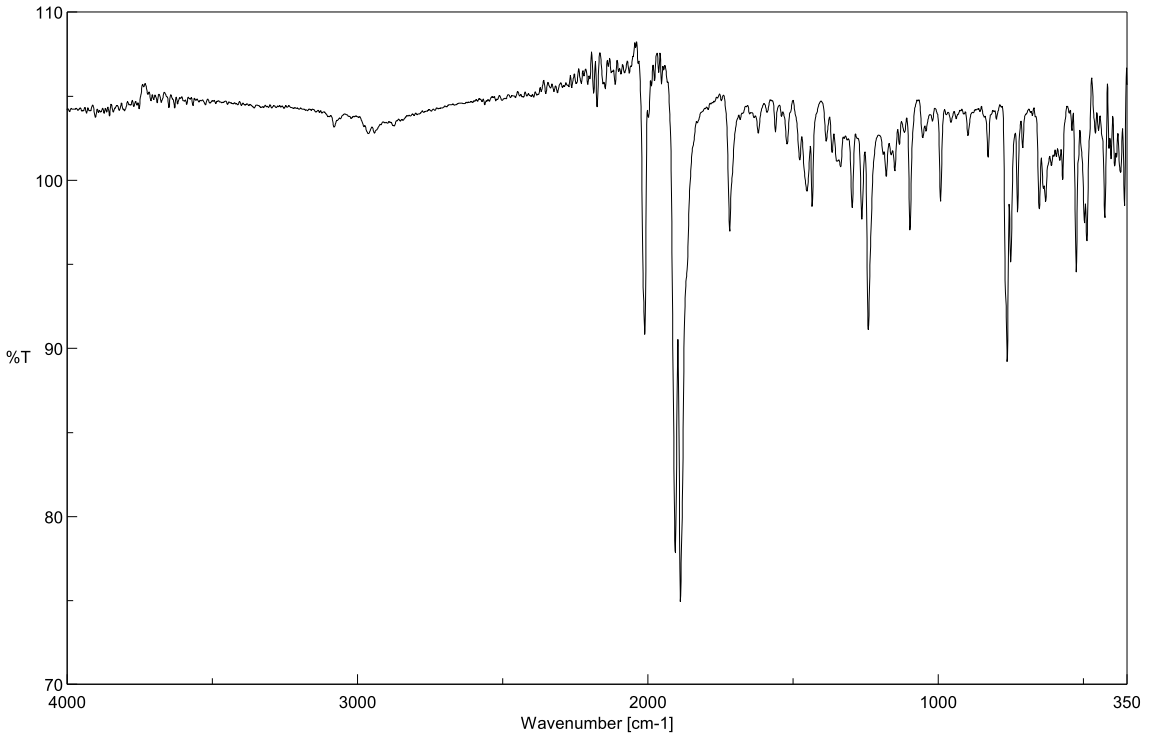


**Figure S21.** IR spectrum of complex **Re3**.


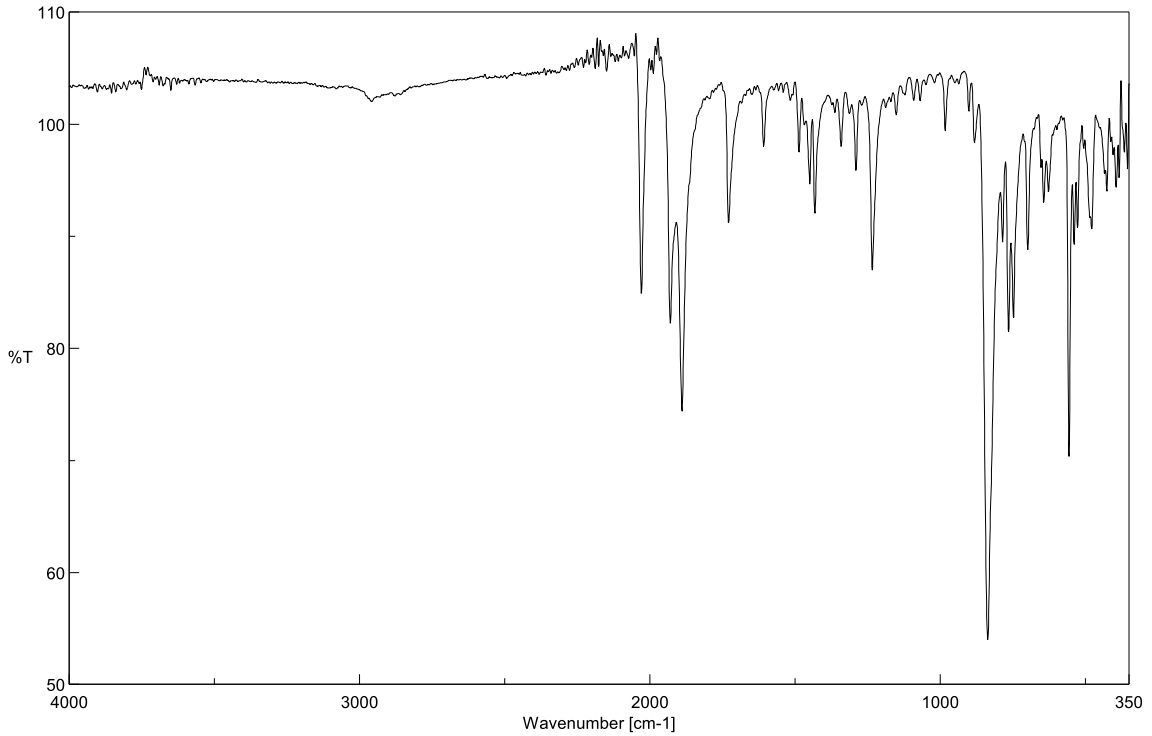


**Figure S22.** IR spectrum of complex **Re4**.


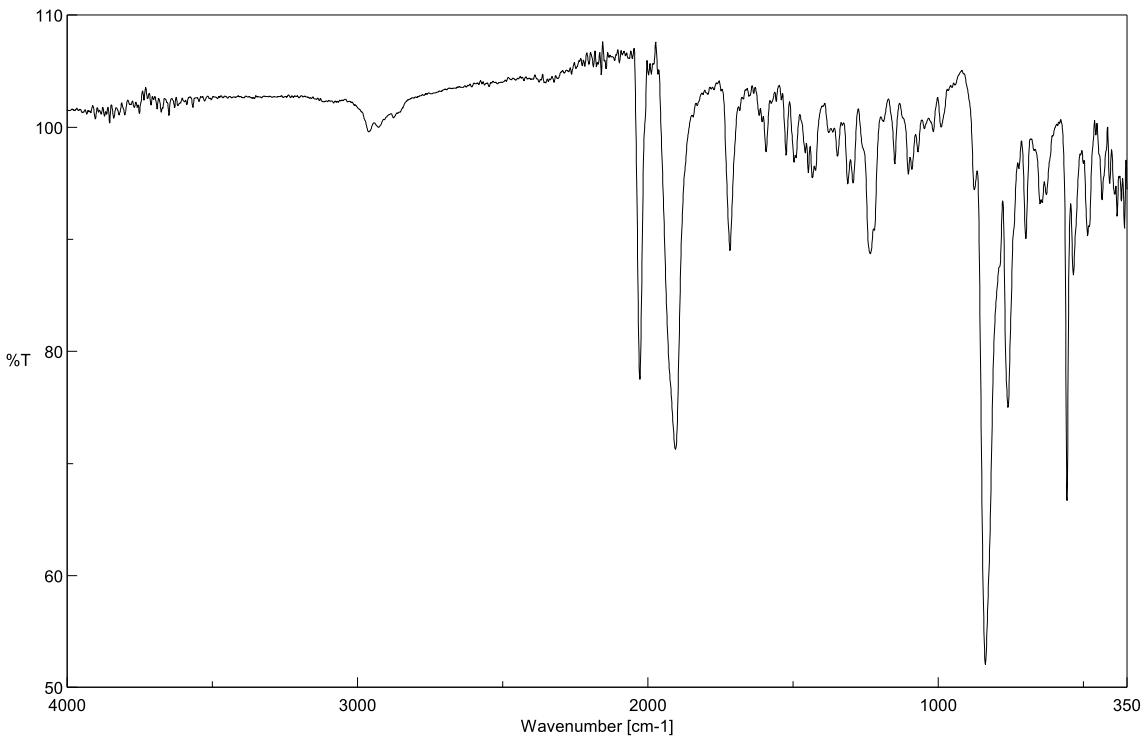


**Figure S23.** IR spectrum of complex **Re5**.


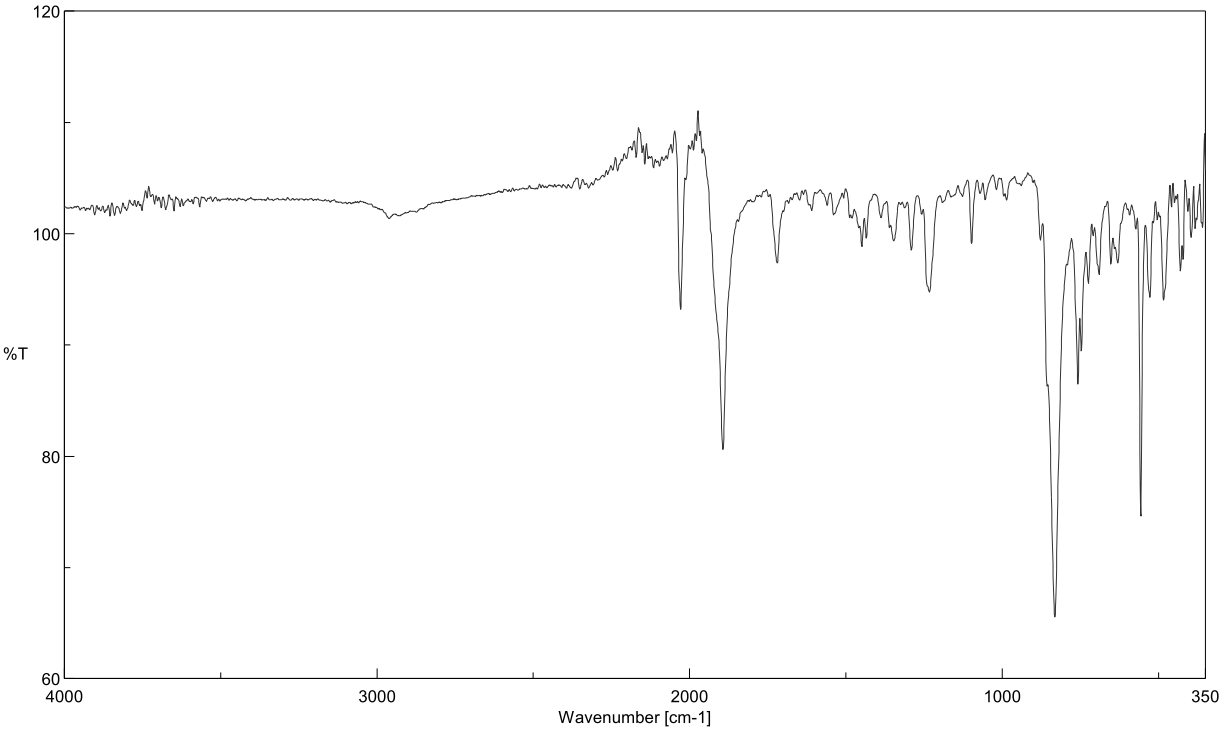


**Figure S24.** IR spectrum of complex **Re6**.


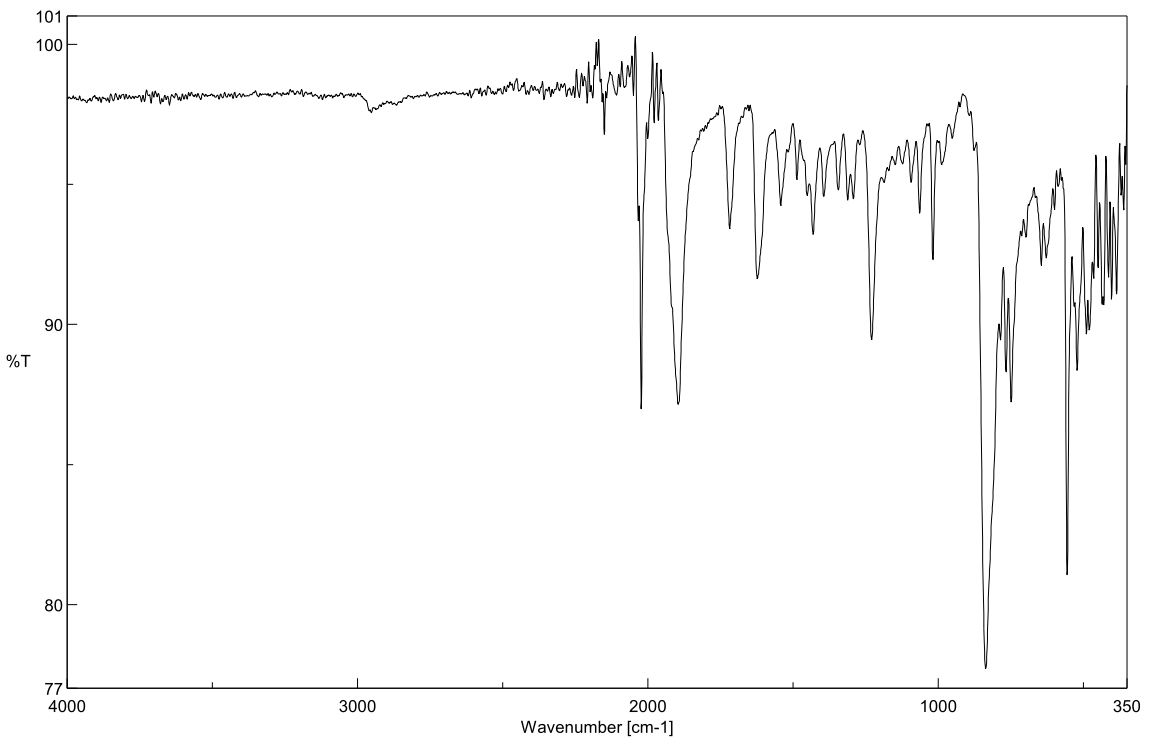


**Figure S25.** IR spectrum of complex **Re7**.


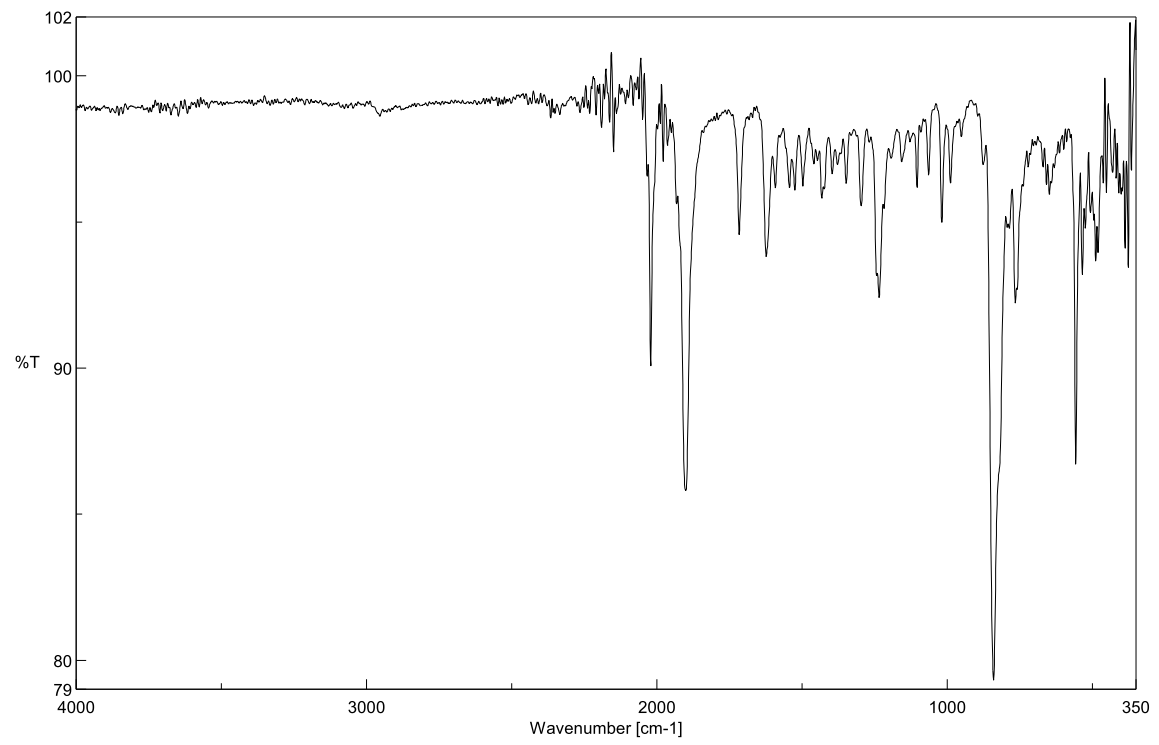


**Figure S26.** IR spectrum of complex **Re8**.


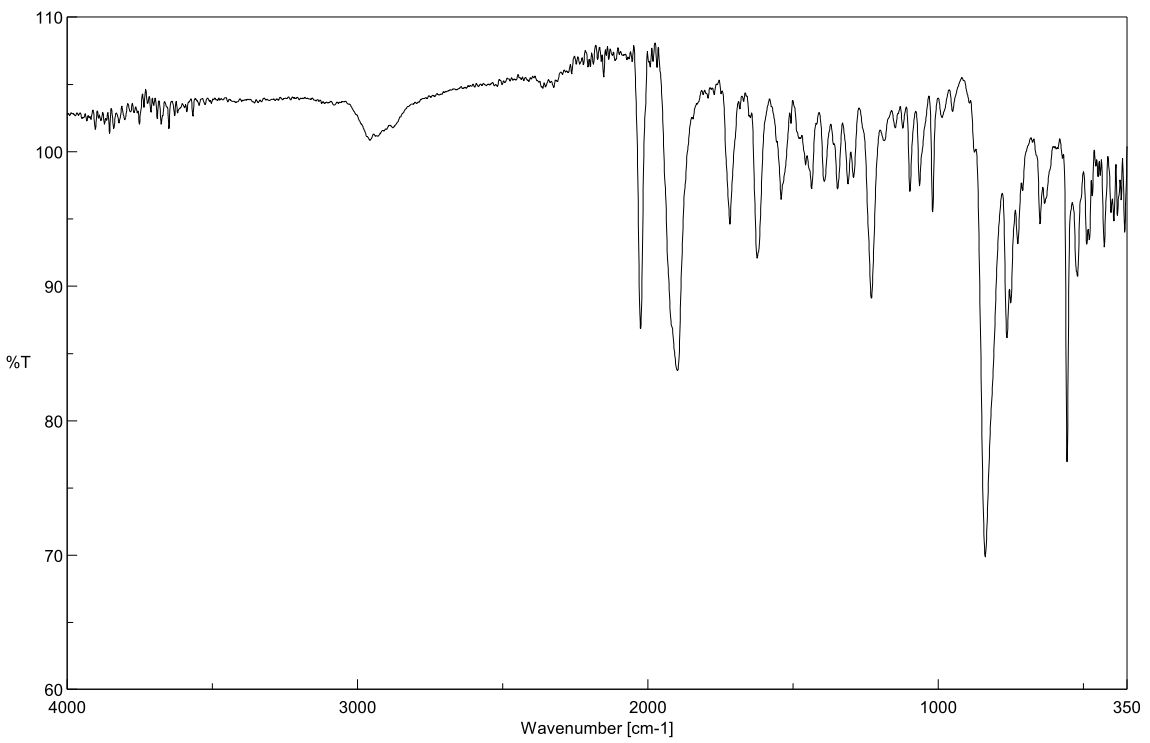


**Figure S27.** IR spectrum of complex **Re9**.

# 4. Mass spectrometry


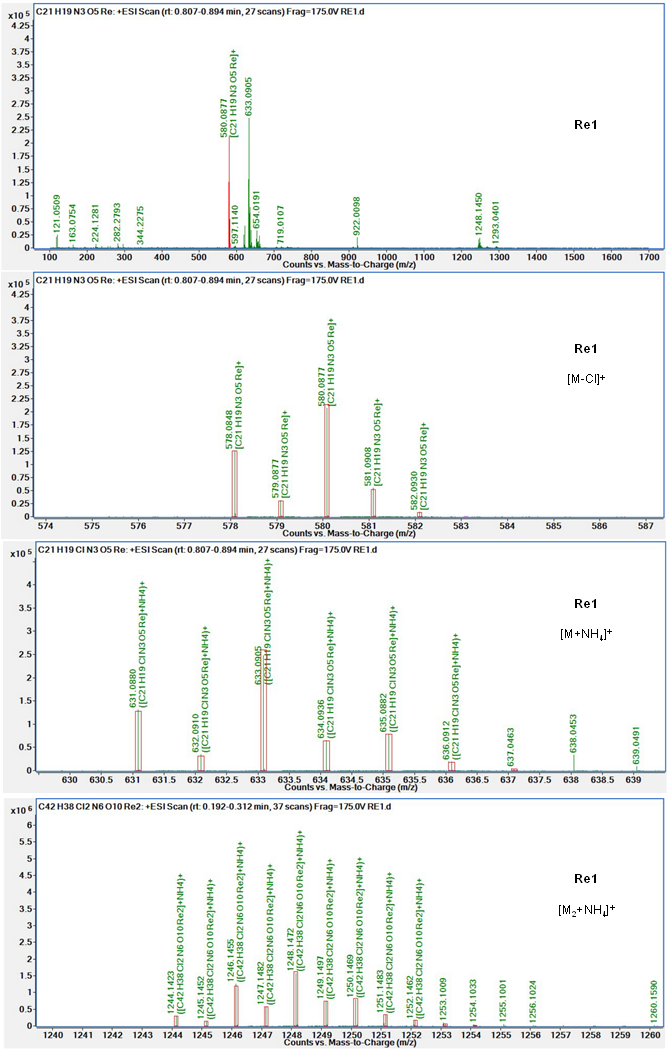


**Figure S28**. ESI-HRMS spectrum of **Re1** in positive ion mode in CH3CN.


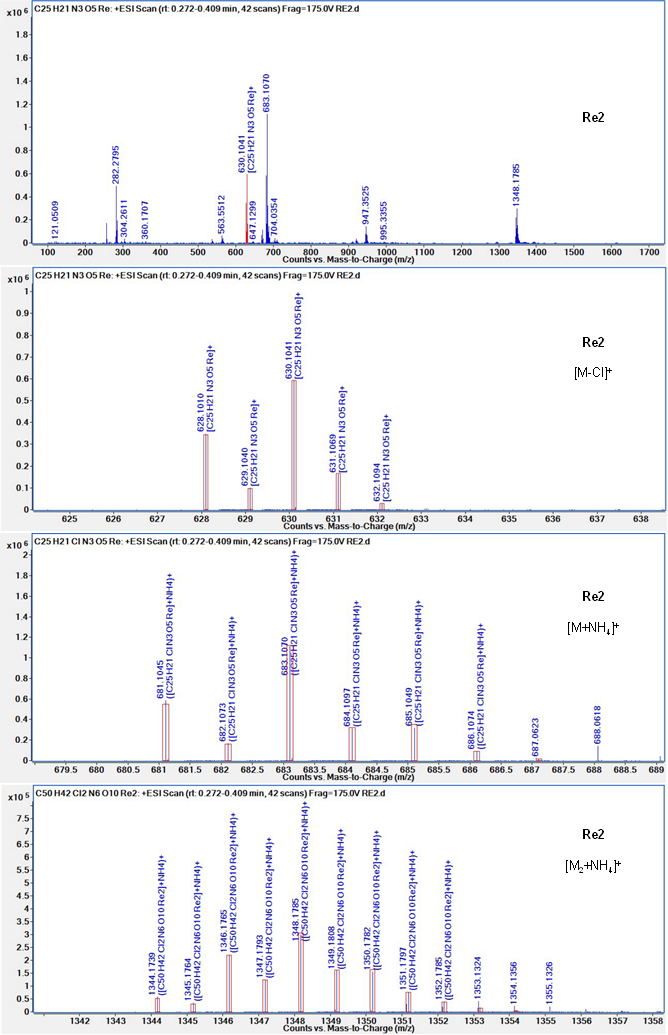


**Figure S29**. ESI-HRMS spectrum of **Re2** in positive ion mode in CH3CN.


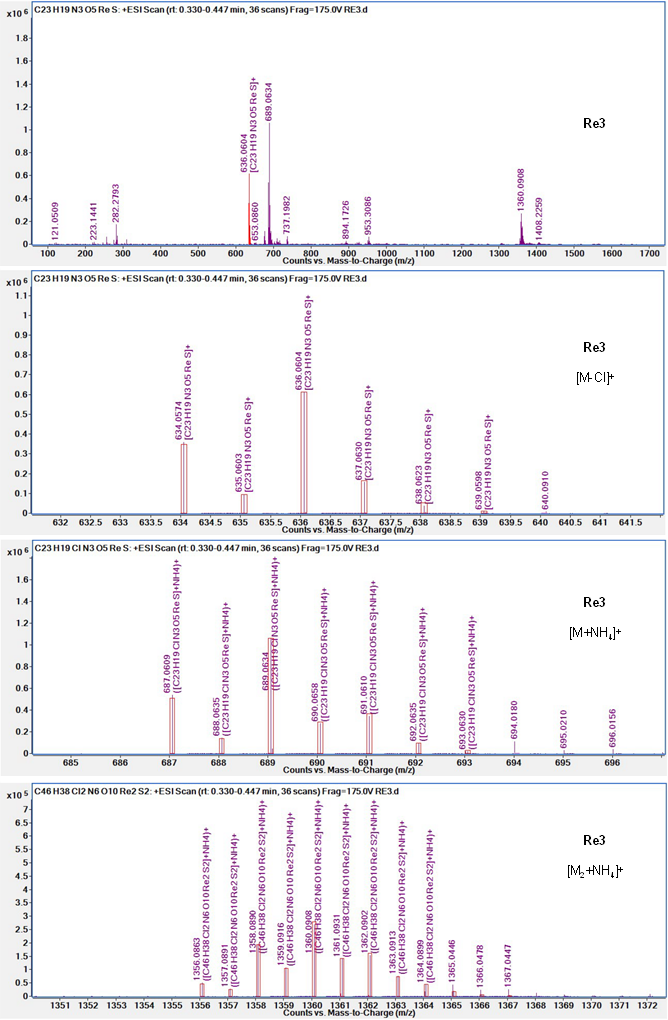


**Figure S30**. ESI-HRMS spectrum of **Re3** in positive ion mode in CH3CN.

# 5. High performance liquid chromatography (HPLC) analysis

| **Table S1.** HPLC method. | | |
| --- | --- | --- |
| Time (min) | 0.05% acetic acid in dH2O | Acetonitrile |
| Start conditions | 98 | 2 |
| 15.00 | 5 | 95 |
| 18.00 | 98 | 2 |
| 20.00 | 5 | 95 |


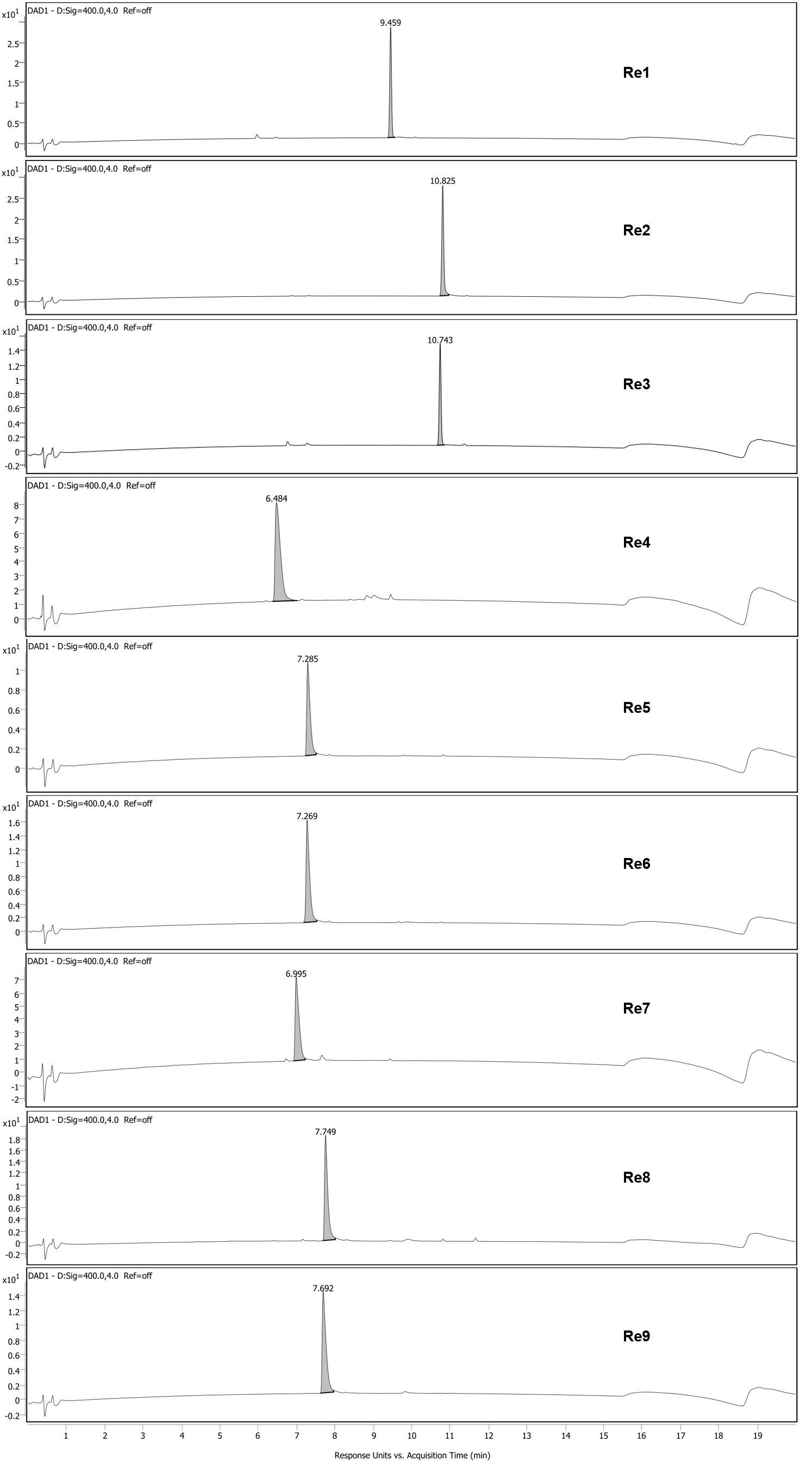


**Figure S31.** RP-HPLC chromatograms with UV detection at 400 nm of complexes **Re1**–**Re9**. Acetonitrile/water (0.1% formic acid) was used as the mobile phase. The purity found was >95% for all the complexes.


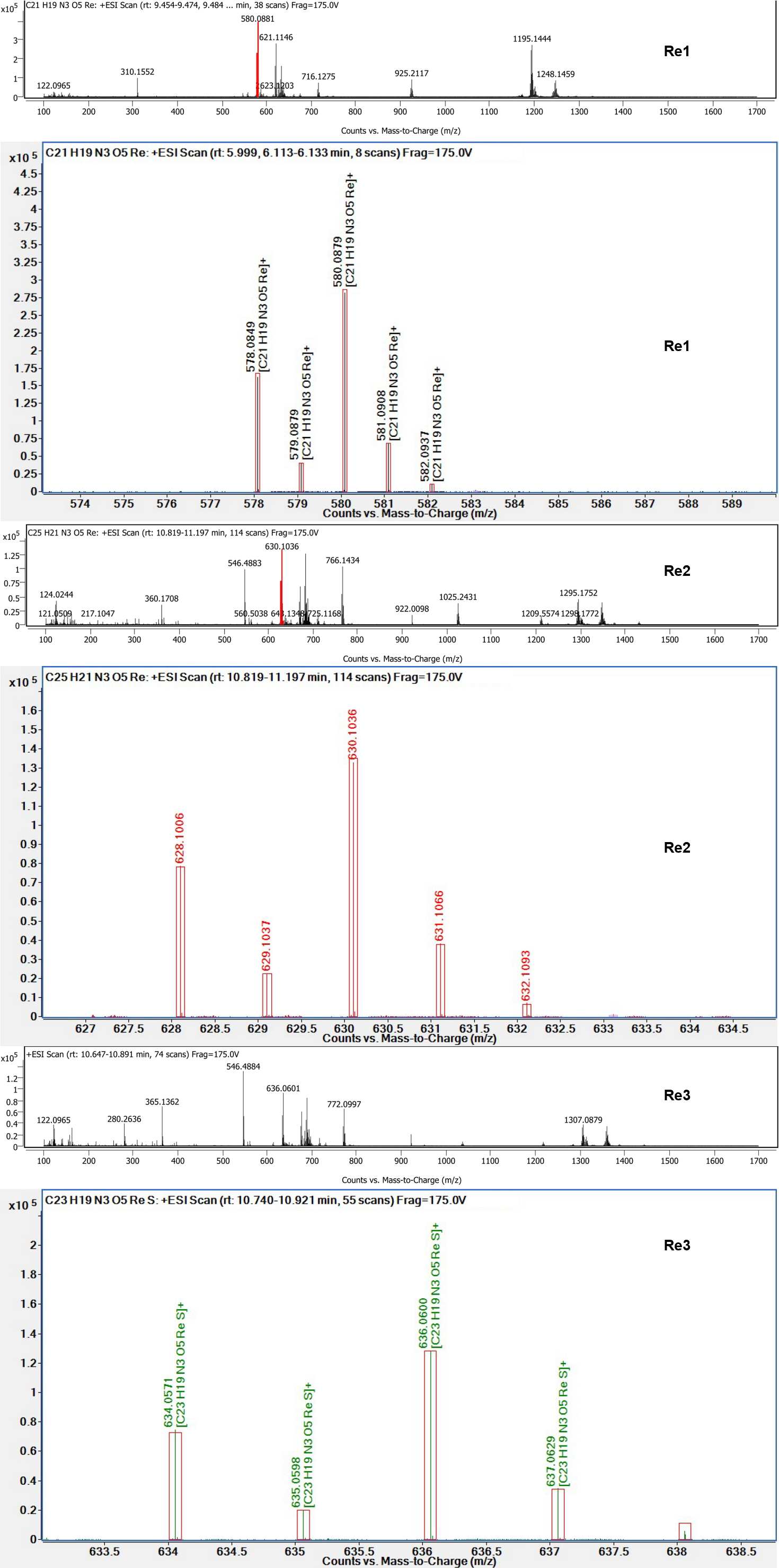


**Figure S32.** Mass spectra of the ∼9–11 min peak of chromatograms of Fig. S31 with peak of interest extracted for complexes **Re1**–**Re3**.


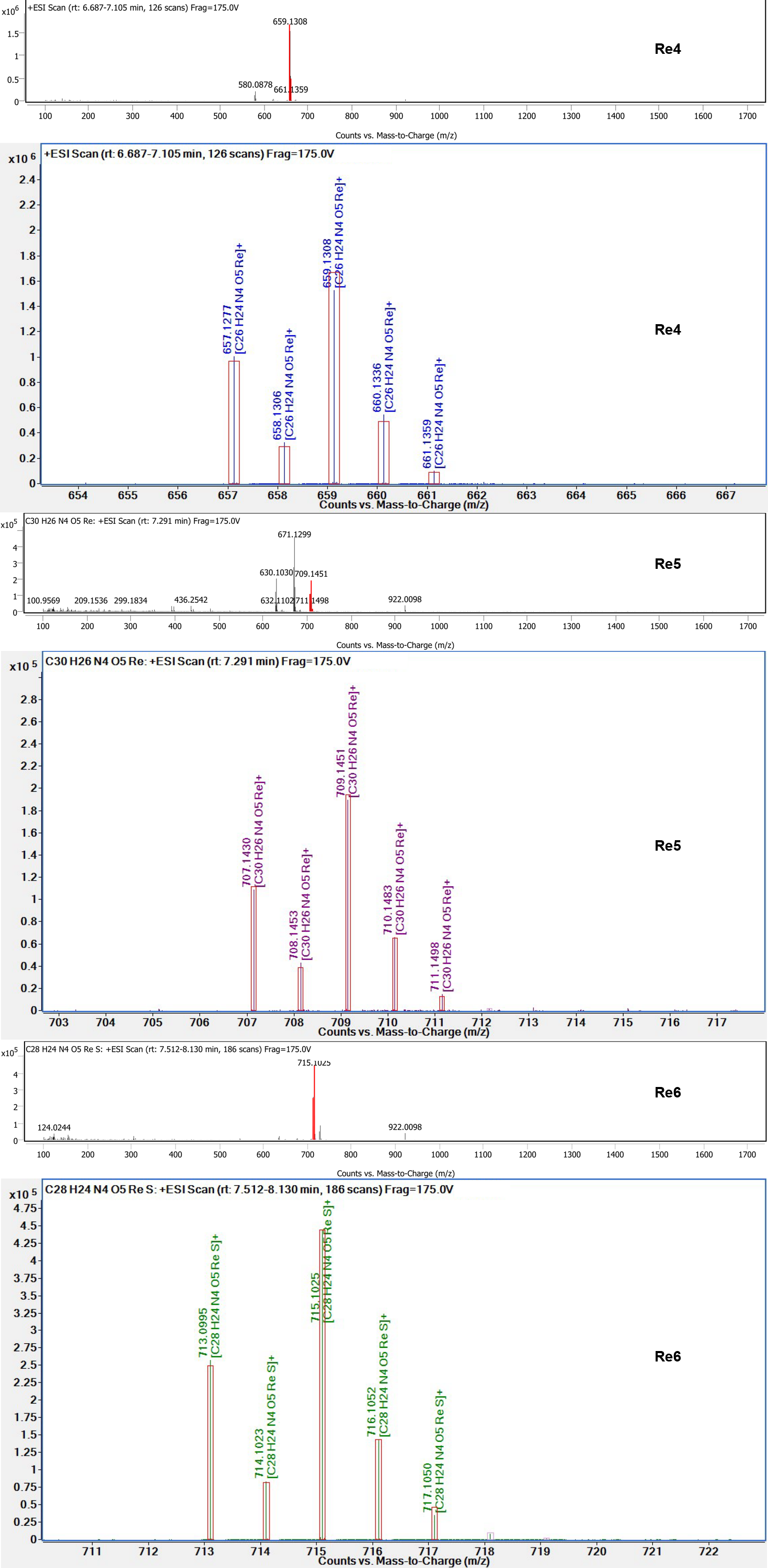


**Figure S33.** Mass spectra of the ∼6–7 min peak of chromatograms of Fig. S31 with peak of interest extracted for complexes **Re4**–**Re6**.


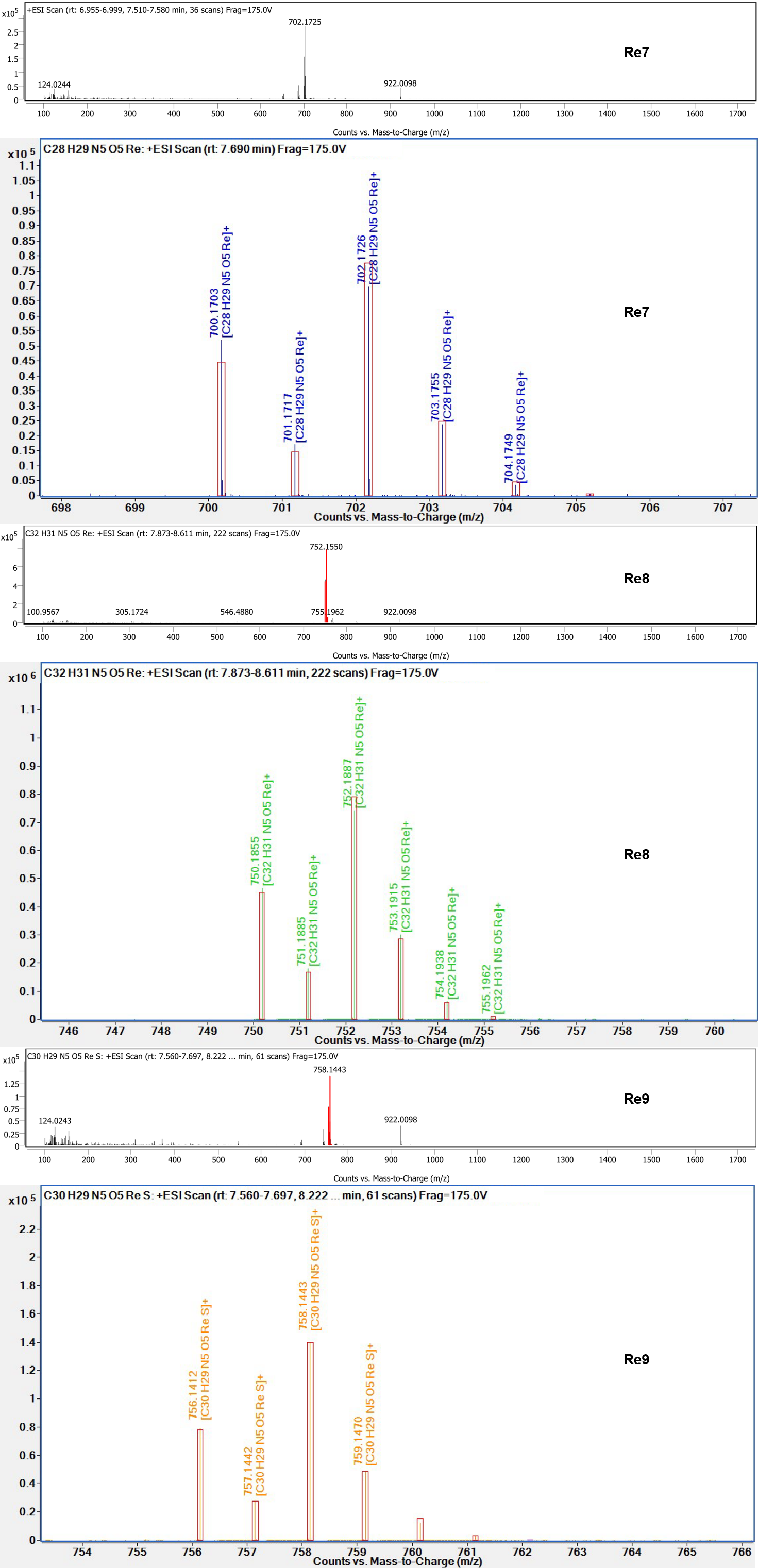


**Figure S34.** Mass spectra of the ∼6–8 min peak of chromatograms of Fig. S31 with peak of interest extracted for complexes **Re7**–**Re9**.

# 6. X-ray structure of complexes Re3, Re3·CHCl3 and Re8

CCDC reference numbers are 2282513 for **Re3**, 2325369 for **Re3**·CHCl3 and 2282514 for **Re8**.

**Table S2a**. Crystal data and structure refinement details for **Re3**.

*Crystal data*

| C23H19ClN3O5ReS | *D*x = 1.939 Mg m-3 |
| --- | --- |
| *Mr* = 671.12 | Mo *K*α radiation,  = 0.71073 Å |
| Orthorhombic, *Pbcn* | Cell parameters from 9484 reflections |
| *a* = 16.6417 (5) Å |  = 2.5–28.7° |
| *b* = 20.7828 (5) Å |  = 5.54 mm-1 |
| *c* = 13.2927 (4) Å | *T* = 100 K |
| *V* = 4597.4 (2) Å3 | Block, yellow |
| *Z* = 8 | 0.16 × 0.09 × 0.05 mm |
| *F*(000) = 2608 |  |

*Data collection*

| Bruker D8 Quest CCD  diffractometer | 5053 reflections with *I* > 2σ(*I*) |
| --- | --- |
| Radiation source: fine-focus sealed tube | *R*int = 0.030 |
|  and  scans | max = 28.7°, min = 2.0° |
| Absorption correction: multi-scan  (*SADABS*; Krause, 2015) | *h* = -2222 |
| *T*min = 0.648, *T*max = 0.746 | *k* = -2828 |
| 96910 measured reflections | *l* = -1717 |
| 5945 independent reflections |  |

*Refinement*

| Refinement on *F*2 | Primary atom site location: structure-invariant direct methods |
| --- | --- |
| Least-squares matrix: full | Secondary atom site location: difference Fourier map |
| *R*[*F*2 > 2σ(*F*2)] = 0.0276  *R*[*F*2, all data] = 0.0374 | Hydrogen site location: inferred from neighbouring sites |
| *wR*[*F*2 > 2σ(*F*2)] = 0.0567  *wR*[*F*2, all data] = 0.0661 | H-atom parameters constrained |
| *S* = 1.13 | *w* = 1/[2(*F*o2) + (0.0164*P*)2 + 25.6488*P*]  where *P* = (*F*o2 + 2*F*c2)/3 |
| 5945 reflections | (Δ/σ)max = 0.001 |
| 309 parameters | max = 3.32 e Å-3 |
| 0 restraints | min = -0.83 e Å-3 |

**Table S2b.** Selected bond distances (Å) and bond angles (°) for **Re3** referring to the atom numbering in the image below:


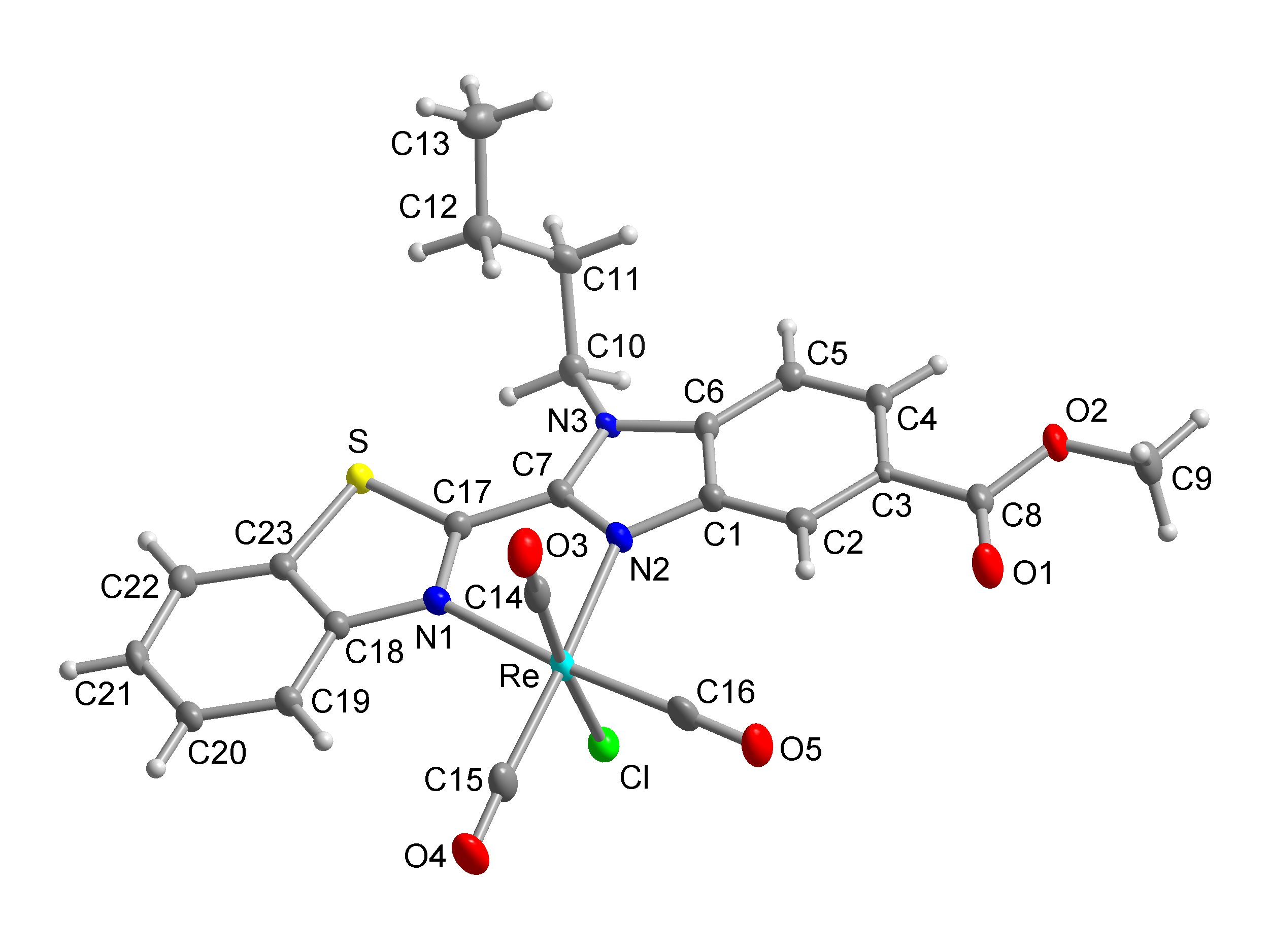


| Re—C16 | 1.908 (4) | O4—C15 | 1.151 (5) |
| --- | --- | --- | --- |
| Re—C15 | 1.921 (4) | O3—C14 | 1.135 (5) |
| Re—C14 | 1.928 (4) | O5—C16 | 1.156 (5) |
| Re—N2 | 2.152 (3) | N1—C17 | 1.324 (4) |
| Re—N1 | 2.208 (3) | N1—C18 | 1.393 (4) |
| Re—Cl | 2.4707 (10) | N2—C7 | 1.323 (4) |
| S—C17 | 1.718 (3) | N2—C1 | 1.381 (4) |
| S—C23 | 1.736 (3) | N3—C7 | 1.358 (4) |
| O1—C8 | 1.199 (5) | N3—C6 | 1.399 (4) |
| O2—C8 | 1.341 (4) | N3—C10 | 1.473 (4) |
| O2—C9 | 1.441 (5) |  |  |
|  |  |  |  |
| C16—Re—C15 | 85.95 (15) | C16—Re—Cl | 89.64 (13) |
| C16—Re—C14 | 89.84 (16) | C15—Re—Cl | 93.91 (14) |
| C15—Re—C14 | 90.67 (17) | C14—Re—Cl | 175.34 (11) |
| C16—Re—N2 | 98.94 (13) | N2—Re—Cl | 81.48 (8) |
| C15—Re—N2 | 173.21 (14) | N1—Re—Cl | 84.53 (8) |
| C14—Re—N2 | 94.02 (13) | C17—S—C23 | 89.41 (16) |
| C16—Re—N1 | 171.04 (13) |  |  |
| C15—Re—N1 | 101.20 (13) | O4—C15—Re | 177.6 (4) |
| C14—Re—N1 | 95.41 (13) | O3—C14—Re | 178.2 (3) |
| N2—Re—N1 | 73.50 (11) | O5—C16—Re | 177.7 (4) |

**Table S2c**. Crystal data and structure refinement details for **Re3**·CHCl3.

When the crystals of **Re3** are grown from a CHCl3 solution with overlayering of hexane or upon solvent evaporation over a few days they are obtained as very small needles. Two data sets from two very tiny needle fragments gave the structure of **Re3** as a CHCl3 solvate, a lower quality one in the triclinic space group P-1 and a somewhat better one in the monoclinic space group P21/c which is reported here.

*Crystal data*

| C23H19ClN3O5ReS·CHCl3 | *F*(000) = 1536 |
| --- | --- |
| *Mr* = 790.49 | *D*x = 1.839 Mg m-3 |
| Monoclinic, *P*21/*c* | Cu *K*α radiation,  = 1.54184 Å |
| *a* = 11.4025 (9) Å | Cell parameters from 3643 reflections |
| *b* = 24.8750 (15) Å |  = 3.9–67.6° |
| *c* = 10.1631 (7) Å |  = 12.80 mm-1 |
| β = 97.960 (8)° | *T* = 150 K |
| *V* = 2854.9 (3) Å3 | Needle, clear orange |
| *Z* = 4 | 0.09 × 0.04 × 0.03 × 0.03 (radius) mm |

*Data collection*

| XtaLAB Synergy, Dualflex, HyPix diffractometer | 5088 independent reflections |
| --- | --- |
| Radiation source: micro-focus sealed X-ray tube, PhotonJet (Cu) X-ray Source | 3142 reflections with *I* > 2σ(*I*) |
| Mirror monochromator | *R*int = 0.167 |
| Detector resolution: 10.0000 pixels mm-1 | max = 67.1°, min = 3.6° |
|  scans | *h* = -1313 |
| Absorption correction: for a sphere with *CrysAlis PRO* 1.171.41.90a (Rigaku Oxford Diffraction, 2020) Spherical absorption correction using equivalent radius and absorption coefficient. Empirical absorption correction using spherical harmonics, implemented in SCALE3 ABSPACK scaling algorithm. | *k* = -2917 |
| *T*min = 0.630, *T*max = 0.651 | *l* = -1210 |
| 21897 measured reflections |  |

*Refinement*

| Refinement on *F*2 | Hydrogen site location: inferred from neighbouring sites |
| --- | --- |
| Least-squares matrix: full | H-atom parameters constrained |
| *R*[*F*2 > 2σ(*F*2)] = 0.0794  *R*[*F*2, all data] = 0.1394 | *w* = 1/[2(*F*o2) + 50.1027*P*]  where *P* = (*F*o2 + 2*F*c2)/3 |
| *wR*[*F*2 > 2σ(*F*2)] = 0.1559  *wR*[*F*2, all data] = 0.1852 | (Δ/σ)max < 0.001 |
| *S* = 1.04 | max = 2.26 e Å-3 |
| 5088 reflections | min = -2.09 e Å-3 |
| 346 parameters | Extinction correction: *SHELXL2017*/1 (Sheldrick 2017), Fc*=kFc[1+0.001xFc2λ3/sin(2θ)]-1/4 |
| 0 restraints | Extinction coefficient: 0.00064 (8) |
| Primary atom site location: dual |  |

**Table S2d.** Selected bond distances (Å) and bond angles (°) for **Re3**·CHCl3 referring to the atom numbering in the image below:


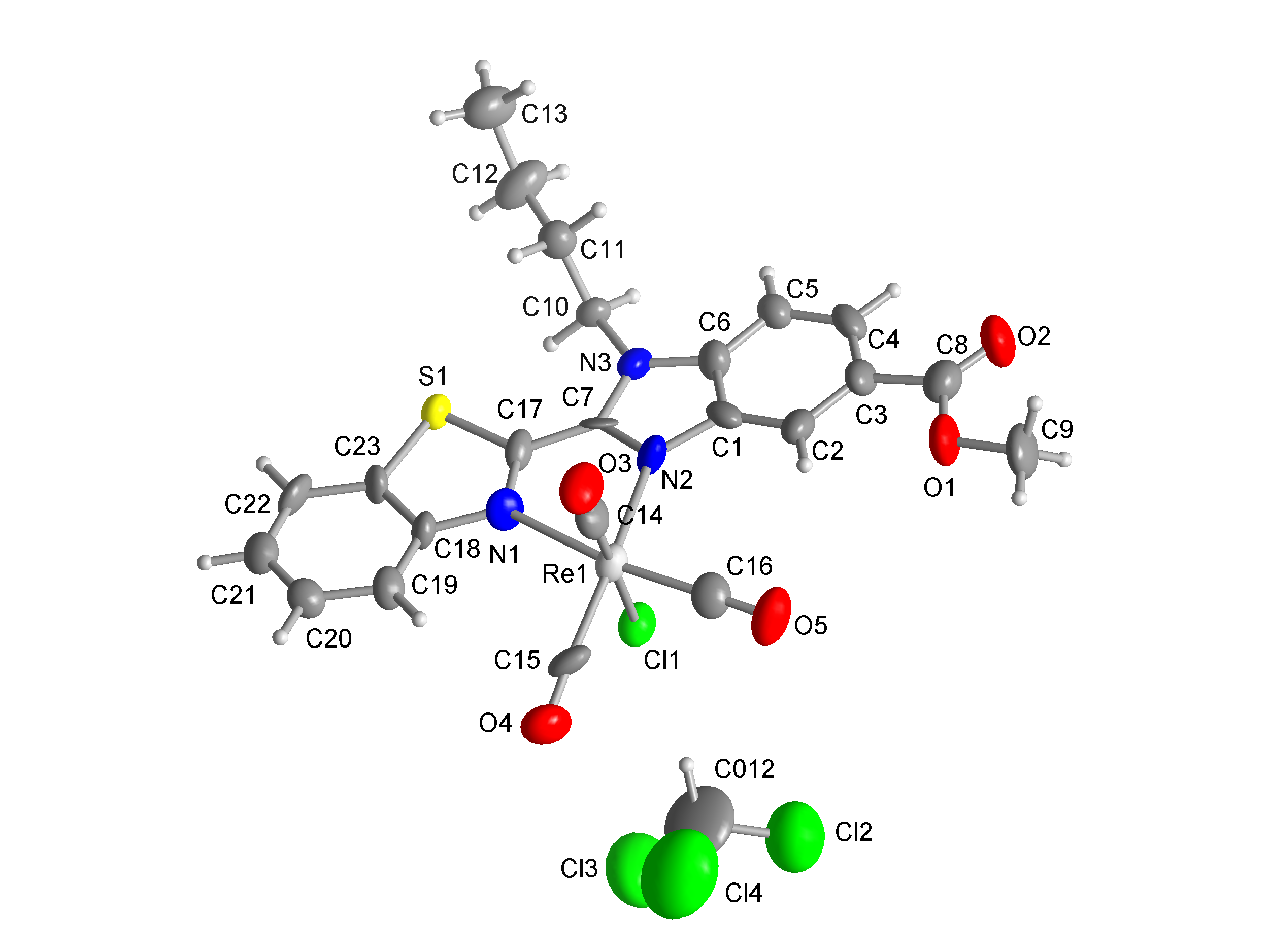


| Re1—Cl1 | 2.489 (4) | O3—C14 | 1.151 (19) |
| --- | --- | --- | --- |
| Re1—N1 | 2.215 (11) | O4—C15 | 1.164 (18) |
| Re1—N2 | 2.167 (12) | O5—C16 | 1.158 (18) |
| Re1—C14 | 1.899 (18) | N1—C17 | 1.299 (18) |
| Re1—C15 | 1.876 (17) | N1—C18 | 1.396 (18) |
| Re1—C16 | 1.896 (17) | N2—C1 | 1.410 (18) |
| S1—C17 | 1.746 (13) | N2—C7 | 1.321 (18) |
| S1—C23 | 1.769 (14) | N3—C6 | 1.379 (19) |
| O1—C8 | 1.35 (2) | N3—C7 | 1.383 (18) |
| O1—C9 | 1.47 (2) | N3—C10 | 1.490 (17) |
| O2—C8 | 1.19 (2) |  |  |
|  |  |  |  |
| N1—Re1—Cl1 | 85.2 (3) | C15—Re1—C16 | 87.8 (7) |
| N2—Re1—Cl1 | 83.0 (4) | C16—Re1—Cl1 | 94.4 (5) |
| N2—Re1—N1 | 73.4 (4) | C16—Re1—N1 | 171.7 (6) |
| C14—Re1—Cl1 | 179.5 (5) | C16—Re1—N2 | 98.3 (6) |
| C14—Re1—N1 | 95.0 (5) | C16—Re1—C14 | 85.5 (7) |
| C14—Re1—N2 | 97.6 (6) | C17—S1—C23 | 88.4 (7) |
| C15—Re1—Cl1 | 88.4 (5) |  |  |
| C15—Re1—N1 | 100.5 (6) | O3—C14—Re1 | 178.5 (15) |
| C15—Re1—N2 | 169.7 (6) | O4—C15—Re1 | 176.4 (13) |
| C15—Re1—C14 | 91.1 (7) | O5—C16—Re1 | 179.0 (16) |


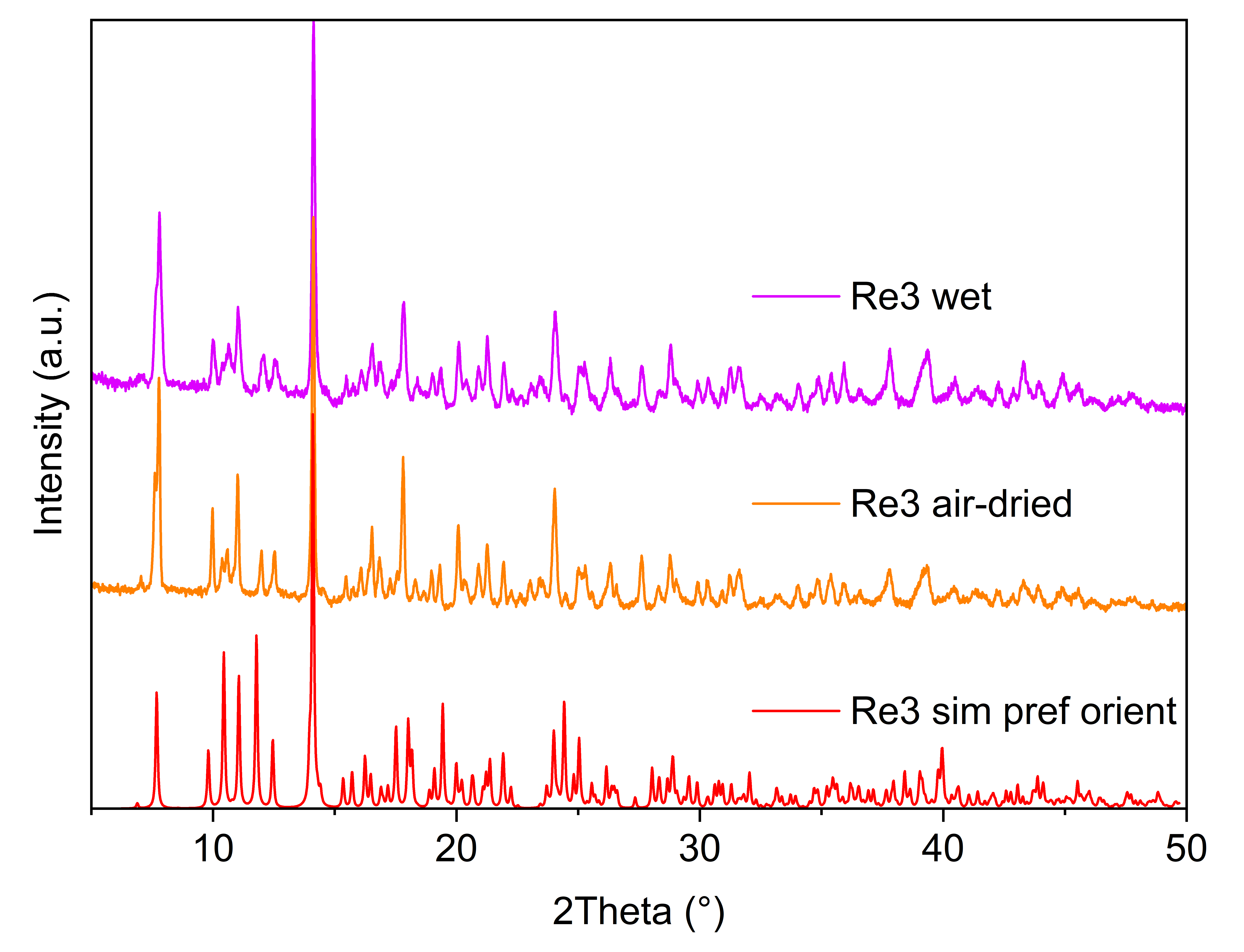


**Figure S35**. Powder X-ray diffraction patterns of small needle crystals of **Re3** which were grown from a CHCl3 solution and measured in the wet and air-dried state at ambient temperature. The simulated diffractogram was obtained from the low-quality single-crystal X-ray data set in the triclinic space group P-1 of **Re3**·CHCl3 by also taking into account a preferred orientation of the needle-shaped crystals along 002 with March-Dollase factor of 0.6. In the simulated diffractograms the peaks are slightly shifted to higher 2theta angles because of the lower temperature (100 K) in the underlying crystal structure data. A lower temperature usually leads to a contraction of the bond lengths and unit cell parameters thereby giving reflections at higher 2theta angles.

PXRD measurements were obtained with a Rigaku MiniFlex (600 W, 40 kV, 15 mA) and Cu‑Kα radiation (λmean = 1.54184 Å) at ambient temperature with a low‑background silicon holder in Bragg-Brentano geometry. The highest reflex was normalized to 1. Simulated powder patterns were derived from the single crystal data with the MERCURY 2020.3.0 software.

**Table S3a.** Crystal data and structure refinement details for **Re8**.

*Crystal data*

| C32H31N5O5Re·F6P·C0.275H0.55Cl0.55·0.79(CH2Cl2) | *D*x = 1.643 Mg m-3 |
| --- | --- |
| *Mr* = 987.23 | Mo *K*α radiation,  = 0.71073 Å |
| Orthorhombic, *Pnna* | Cell parameters from 9298 reflections |
| *a* = 21.5188 (18) Å |  = 2.4–29.6° |
| *b* = 21.545 (2) Å |  = 3.30 mm-1 |
| *c* = 17.2204 (14) Å | *T* = 100 K |
| *V* = 7983.8 (12) Å3 | Needle, yellow |
| *Z* = 8 | 0.33 × 0.05 × 0.02 mm |
| *F*(000) = 3894 |  |

*Data collection*

| Bruker D8 Quest CCD  diffractometer | 7758 reflections with *I* > 2σ(*I*) |
| --- | --- |
| Radiation source: fine-focus sealed tube | *R*int = 0.038 |
|  and  scans | max = 26.4°, min = 1.8° |
| Absorption correction: multi-scan  (*SADABS*; Krause, 2015) | *h* = -2626 |
| *T*min = 0.576, *T*max = 0.746 | *k* = -2626 |
| 133853 measured reflections | *l* = -2121 |
| 8199 independent reflections |  |

*Refinement*

| Refinement on *F*2 | 0 restraints |
| --- | --- |
| Least-squares matrix: full | Hydrogen site location: inferred from neighbouring sites |
| *R*[*F*2 > 2σ(*F*2)] = 0.0818  *R*[*F*2 > 2σ(*F*2)] = 0.0848 | H-atom parameters constrained |
| *wR*[*F*2 > 2σ(*F*2)] = 0.1829  *wR*[*F*2, all data] = 0.1841 | *w* = 1/[2(*F*o2) + (0.0345*P*)2 + 168.3022*P*]  where *P* = (*F*o2 + 2*F*c2)/3 |
| *S* = 1.25 | (Δ/σ)max < 0.001 |
| 8199 reflections | max = 2.64 e Å-3 |
| 539 parameters | min = -3.79 e Å-3 |

**Table S3b.** Selected bond distances (Å) and bond angles (°) for **Re8** referring to the atom numbering in the image below:


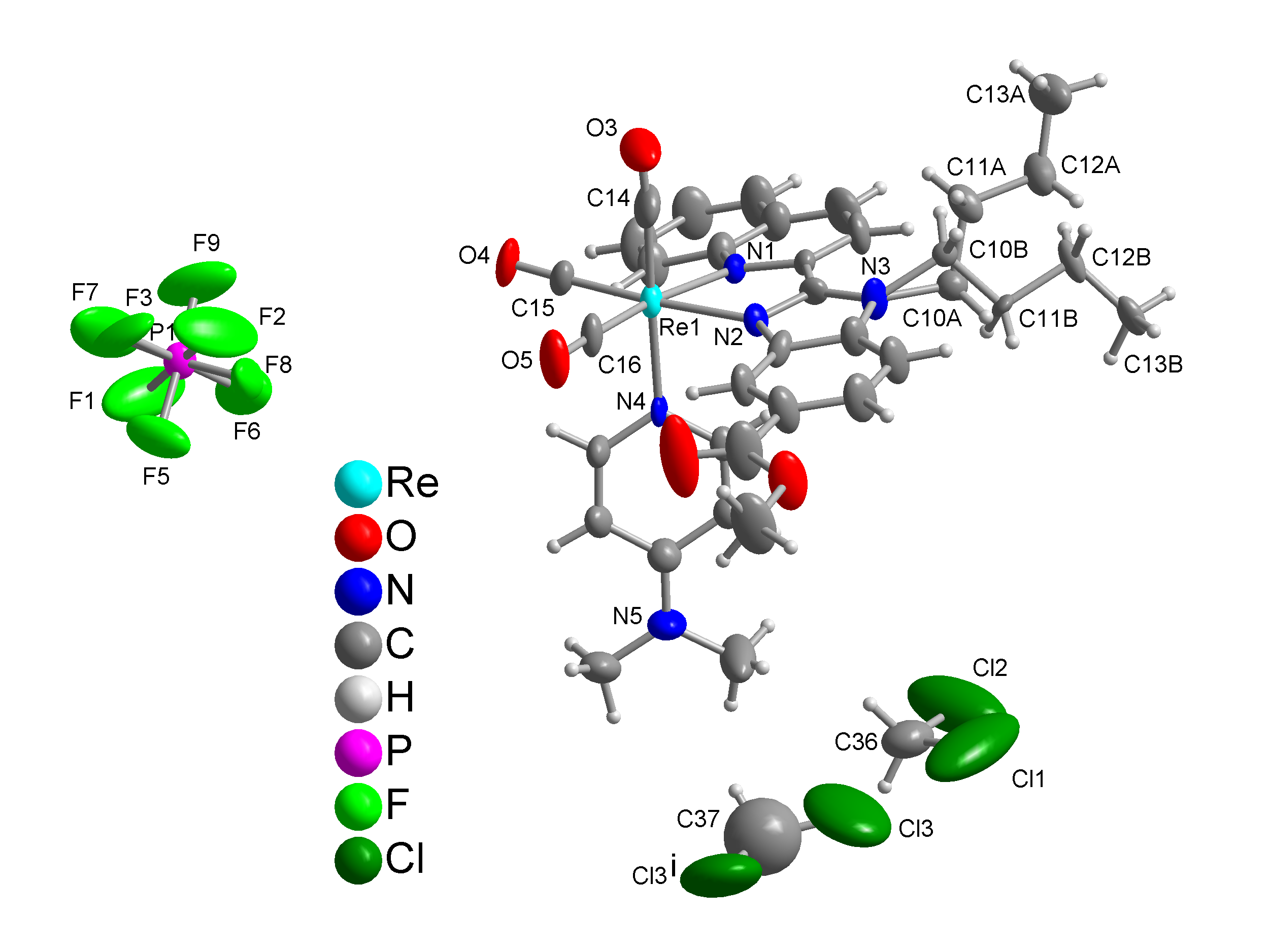


The molecular structure of complex **Re8** shows also the butyl chain disorder and the two CH2Cl2 solvent molecules (50% thermal ellipsoids; H atoms with arbitrary radii). The atoms C10A, C10B and C37 could only be isotropically refined. Symmetry transformation (i) -x+1/2, -y+1, z.

| Re1—C16 | 1.889 (11) | N1—C18 | 1.372 (14) |
| --- | --- | --- | --- |
| Re1—C15 | 1.910 (10) | N1—C17 | 1.377 (12) |
| Re1—C14 | 1.915 (15) | N2—C7 | 1.322 (12) |
| Re1—N2 | 2.145 (8) | N2—C1 | 1.370 (13) |
| Re1—N4 | 2.202 (8) | N3—C7 | 1.347 (13) |
| Re1—N1 | 2.254 (8) | N3—C6 | 1.375 (14) |
| O1—C8 | 1.198 (17) | N3—C10A | 1.51 (2) |
| O2—C8 | 1.342 (15) | N3—C10B | 1.53 (2) |
| O2—C9 | 1.427 (16) | N4—C30 | 1.355 (13) |
| O3—C14 | 1.152 (17) | N4—C26 | 1.357 (12) |
| O4—C15 | 1.153 (13) | N5—C28 | 1.337 (15) |
| O5—C16 | 1.164 (14) | N5—C31 | 1.470 (19) |
|  |  | N5—C32 | 1.480 (17) |
|  |  |  |  |
| C16—Re1—C15 | 85.3 (4) | C16—Re1—N1 | 171.9 (4) |
| C16—Re1—C14 | 88.5 (6) | C15—Re1—N1 | 102.5 (4) |
| C15—Re1—C14 | 87.7 (5) | C14—Re1—N1 | 94.1 (4) |
| C16—Re1—N2 | 99.4 (4) | N2—Re1—N1 | 72.9 (3) |
| C15—Re1—N2 | 175.1 (4) | N4—Re1—N1 | 86.1 (3) |
| C14—Re1—N2 | 90.9 (4) | N2—Re1—N4 | 86.9 (3) |
| C16—Re1—N4 | 91.0 (5) |  |  |
| C15—Re1—N4 | 94.5 (4) | O3—C14—Re1 | 175.9 (11) |
| C14—Re1—N4 | 177.6 (5) | O4—C15—Re1 | 177.3 (11) |
|  |  | O5—C16—Re1 | 176.9 (13) |
|  |  |  |  |

**Analysis of “Short Ring-Interactions" for possible π-stacking interactions in Re3 and Re8**

The PLATON3,4-listing "Analysis of Short Ring-Interactions" for possible π-stacking interactions yielded significant π-stacking for **Re3** with rather short centroid-centroid contacts (<3.8 Å), near parallel ring planes (alpha < 10° to ~0° or even exactly 0° by symmetry), small slip angles (β, γ <25°) and vertical displacements (slippage <1.5 Å) which translate into a sizable overlap of the aryl-plane areas (Scheme S2, Table S4, Figure S36).5,6

In **Re8** there are much fewer π-stacking interactions (Table S6, Figure S37) between the cations because of the intermittant PF6 anions and solvent molecules.

**Scheme S2.** Graphical presentation of the parameters used for the description of π-π stacking.

In addition, there are minor C-H···O/Cl contacts in **Re3** (Table S5) and some weak C-H···π contacts in **Re8** (Table S7).

Significant intermolecular C-H···π contacts start below around 2.7 Å for the (C-)H···ring centroid distances with H-perp also starting at below 2.6-2.7 Å and C-H··Cg > 145° (Scheme **S3**).7–12

**Scheme S3**. Graphical presentation of the parameters used for the description of CH-π interactions.8

**Table S4.** Packing Analysis for **Re3** for possible  interactions.

(see Scheme S2 for explanation).

=============================================================================

Analysis of Short Ring-Interactions with Cg-Cg Distances < 4.0 Ang., Alpha < 20.000 Deg. and Beta < 60.0 Deg.

=============================================================================

- Cg(I) = Plane number I (= ring number in () above)

- Alpha = Dihedral Angle between Planes I and J (Deg)

- Beta = Angle Cg(I)-->Cg(J) or Cg(I)-->Me vector and normal to plane I (Deg)

- Gamma = Angle Cg(I)-->Cg(J) vector and normal to plane J (Deg)

- Cg-Cg = Distance between ring Centroids (Ang.)

- CgI_Perp = Perpendicular distance of Cg(I) on ring J (Ang.)

- CgJ_Perp = Perpendicular distance of Cg(J) on ring I (Ang.)

- Slippage = Distance between Cg(I) and Perpendicular Projection of Cg(J) on Ring I (Ang).

Cg(I) Res(I) Cg(J) [ ARU(J)] Cg-Cg Alpha Beta Gamma CgI_Perp CgJ_Perp Slippage

Cg(2) [ 1] -> Cg(5) [ 4555.01] 3.693(2) 2.27(18) 23.5 22.3 3.4162(14) 3.3859(16) 1.474

Cg(4) [ 1] -> Cg(4) [ 4655.01] 3.801(2) 7.13(18) 27.6 27.6 3.3685(15) 3.3685(15) 1.761

Cg(4) [ 1] -> Cg(5) [ 3555.01] 3.590(2) 2.77(18) 22.0 22.8 -3.3102(16) -3.3292(15) 1.343

Cg(5) [ 1] -> Cg(2) [ 4555.01] 3.693(2) 2.27(18) 22.3 23.5 3.3859(16) 3.4161(14) 1.402

Cg(5) [ 1] -> Cg(4) [ 3455.01] 3.590(2) 2.77(18) 22.8 22.0 -3.3293(15) -3.3102(16) 1.390

Cg(5) [ 1] -> Cg(5) [ 4555.01] 3.493(2) 2.68(18) 13.6 13.6 3.3944(16) 3.3944(16) 0.823

Cg(6) [ 1] -> Cg(4) [ 3455.01] 3.8914(19) 3.41(15) 31.8 32.4 -3.2871(12) -3.3083(16) 2.049

Cg(6) [ 1] -> Cg(5) [ 4555.01] 3.4110(19) 2.42(15) 3.1 3.1 3.4061(12) 3.4061(16) 0.184

Cg(6) [ 1] -> Cg(6) [ 4555.01] 3.5962(16) 2.00(11) 18.6 18.6 3.4079(12) 3.4080(12) 1.148

Cg(7) [ 1] -> Cg(5) [ 3555.01] 3.9836(19) 2.97(15) 32.9 34.1 -3.2987(12) -3.3459(15) 2.162

[ 3455] = -1/2+X,1/2-Y,-Z

[ 3555] = 1/2+X,1/2-Y,-Z

[ 4555] = -X,Y,1/2-Z

[ 4655] = 1-X,Y,1/2-Z

Cg2 = centroid of ring S-N1-C17-C18-C23

Cg4 = centroid of ring C1-C2-C3-C4-C5-C6

Cg5 = centroid of ring C18-C19-C20-C21-C22-C23

Cg6 = centroid of ring S-N1-C17-C18-C19-C20-C21-C22-C23

Cg7 = centroid of ring N2-N3-C1-C2-C3-C4-C5-C6-C7


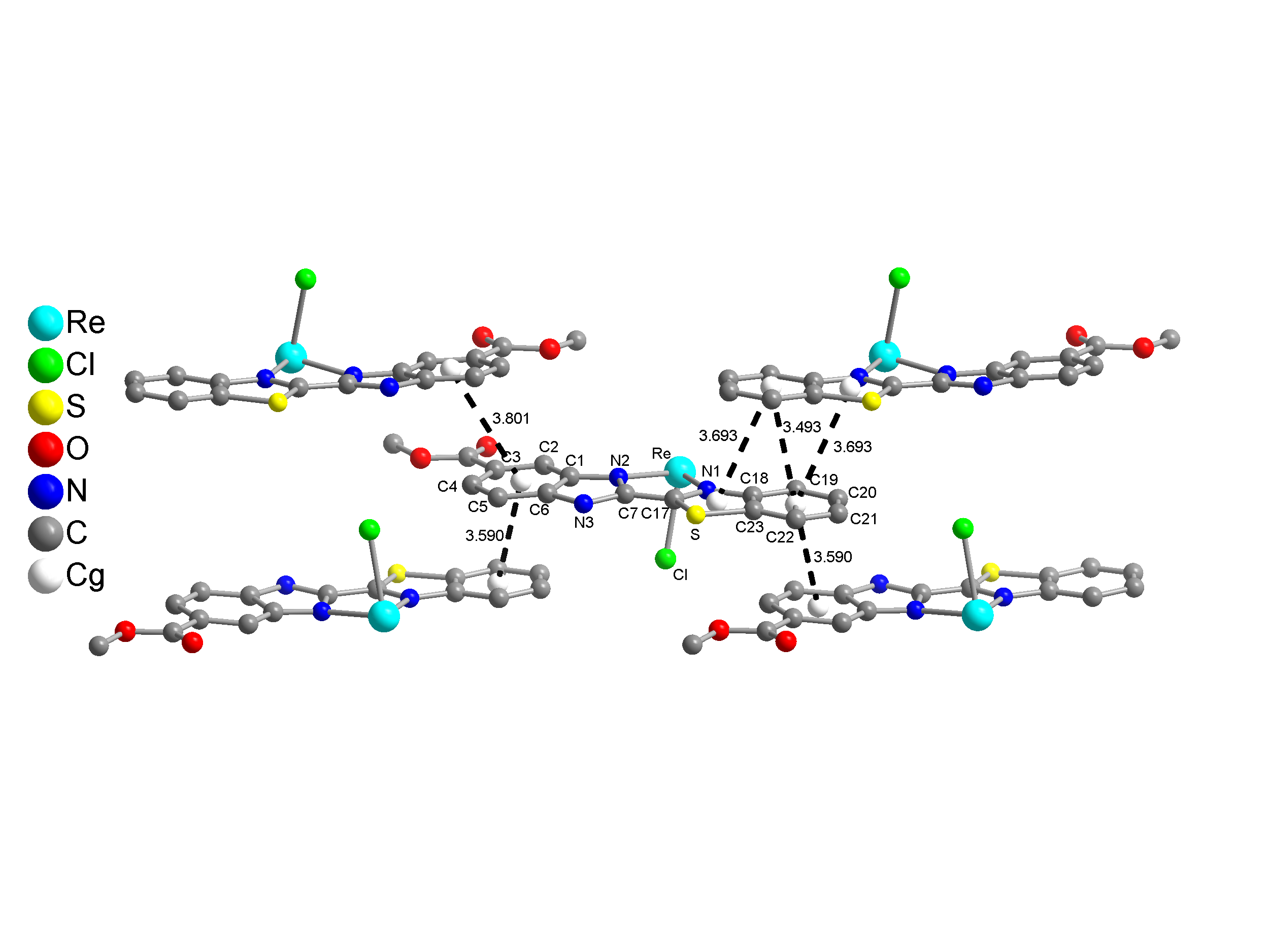


**Figure S36.** π-π interactions in the packing of **Re3** in the crystal, indicated as dashed black lines with the centroid-centroid contact given in Å. Further details of these π-π interactions, including the symmetry transformations are listed in the above Table S4 (Cg = ring centroid). Hydrogen atoms, the butyl group and the CO ligands are not shown for clarity. The contacts involving the centroids of two anellated rings (Cg6, Cg7 in Table S4) are also not depicted.

**Table S5.** Analysis of *inter*molecular C-H...X Interactions in **Re3**.

===================================================================================

Analysis of Potential Hydrogen Bonds and Schemes with d(D...A) < R(D)+R(A)+0.50, d(H...A) < R(H)+R(A)-0.12 Ang., D-H...A > 100.0 Deg

Nr Typ Res Donor --- H....Acceptor [ ARU ] D - H H...A D...A D - H...A

------------------------------------------------------------------------------------------------------------------------------------

2 1 C(4) --H(4) ..Cl [ 3555.01] 0.95 2.63 3.410(3) 140

4 1 C(12) --H(12A) ..Cl [ 2555.01] 0.99 2.75 3.630(4) 148

5 1 C(22) --H(22) ..O(1) [ 7555.01] 0.95 2.29 3.227(4) 169

:: No Classic Hydrogen Bonds Found

Translation of ARU-Code to CIF and Equivalent Position Code

===========================================================

[ 3555.] = [ 4_555] = 1/2+x,1/2-y,-z

[ 7555.] = [ 8_665] = 1/2-x,1/2+y,z

[ 2555.] = [ 2_555] = 1/2-x,1/2-y,1/2+z

**Table S6.** Packing Analysis for **Re8** for possible  interactions.

(see Scheme S2 for explanation).

=============================================================================

Analysis of Short Ring-Interactions with Cg-Cg Distances < 4.0 Ang., Alpha < 20.000 Deg. and Beta < 60.0 Deg.

=============================================================================

- Cg(I) = Plane number I (= ring number in () above)

- Alpha = Dihedral Angle between Planes I and J (Deg)

- Beta = Angle Cg(I)-->Cg(J) or Cg(I)-->Me vector and normal to plane I (Deg)

- Gamma = Angle Cg(I)-->Cg(J) vector and normal to plane J (Deg)

- Cg-Cg = Distance between ring Centroids (Ang.)

- CgI_Perp = Perpendicular distance of Cg(I) on ring J (Ang.)

- CgJ_Perp = Perpendicular distance of Cg(J) on ring I (Ang.)

- Slippage = Distance between Cg(I) and Perpendicular Projection of Cg(J) on Ring I (Ang).

Cg(I) Res(I) Cg(J) [ ARU(J)] Cg-Cg Alpha Beta Gamma CgI_Perp CgJ_Perp Slippage

Cg(5) [ 1] -> Cg(5) [ 3556.01] 3.903(8) 6.5(7) 22.6 22.6 3.605(6) 3.604(6) 1.498

Cg(6) [ 1] -> Cg(6) [ 3555.01] 3.696(9) 6.5(8) 20.5 20.5 3.463(7) 3.463(7) 1.293

[ 3556] = X,1/2-Y,3/2-Z

[ 3555] = X,1/2-Y,1/2-Z

Cg5 = centroid of ring C1-C2-C3-C4-C5-C6

Cg6 = centroid of ring C18-C19-C20-C21-C22-C23


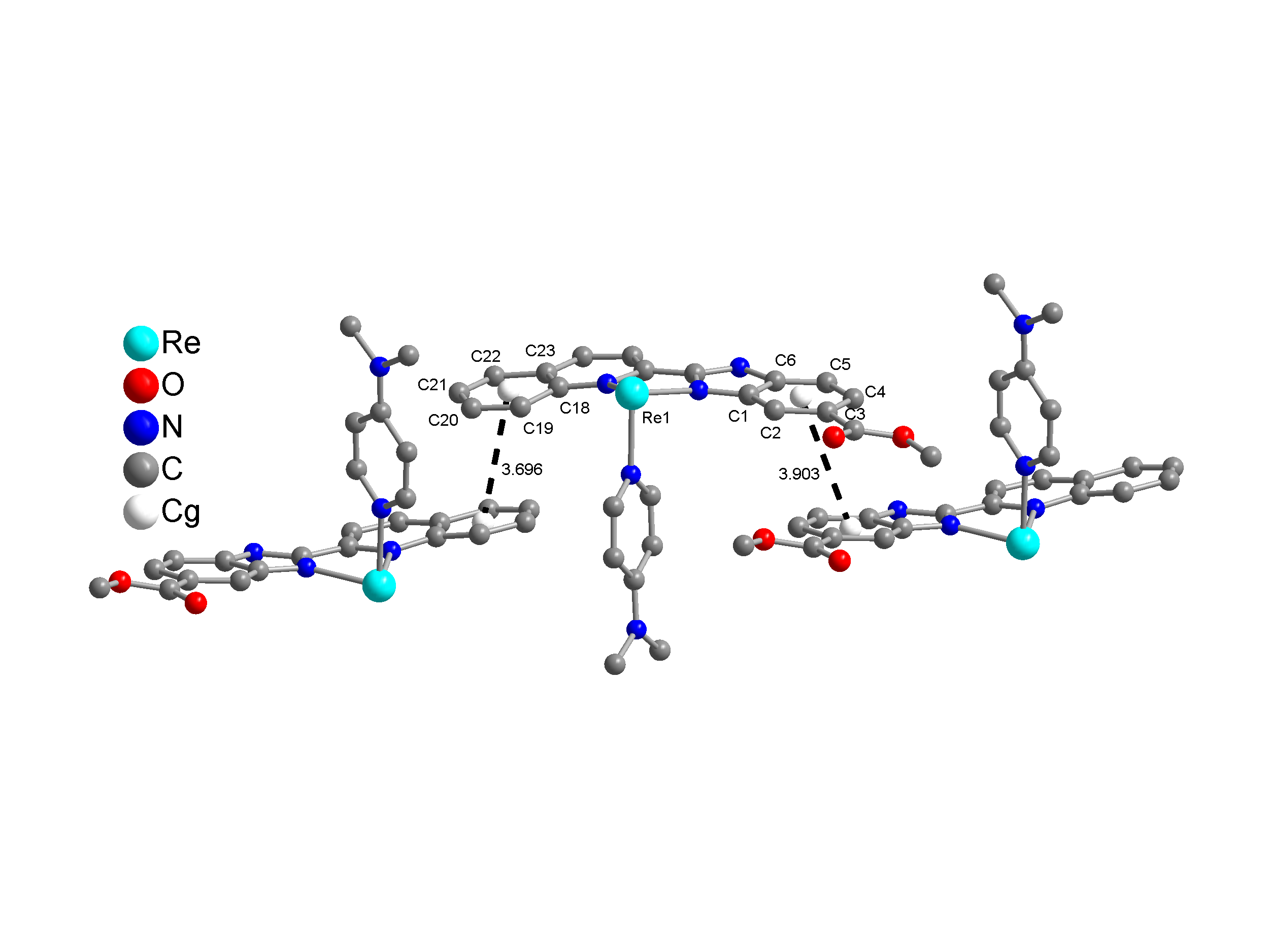


**Figure S37.** π-π interactions in the packing of the cation of **Re8** in the crystal, indicated as dashed black lines with the centroid-centroid contact given in Å. Further details of these π-π interactions, including the symmetry transformations are listed in the above Table S6 (Cg = ring centroid). Hydrogen atoms, the butyl group and the CO ligands are not shown for clarity.

**Table S7.** Analysis of *inter*molecular C-H...Cg(Pi-Ring) Interactions (H..Cg < 3.0 Ang. - Gamma < 30.0 Deg) in **Re8** (see Scheme S3 for explanation).

- Cg(J) = Center of gravity of ring J (Plane number above)

- H-Perp = Perpendicular distance of H to ring plane J

- Gamma = Angle between Cg-H vector and ring J normal

- C-H..Cg = C-H-Cg angle (degrees)

- C..Cg = Distance of X to Cg (Angstrom)

- C-H, Pi = Angle of the X-H bond with the Pi-plane (i.e.' Perpendicular = 90 degrees, Parallel = 0 degrees)

X--H(I) Res(I) Cg(J) [ ARU(J)] H..Cg H-Perp Gamma X-H..Cg X..Cg X-H,Pi

C(20) -H(20) [ 1] -> Cg(4) [ 3555.01] 2.95 2.78 19.53 170 3.884(15) 81

C(36) -H(36A) [ 3] -> Cg(5) [ 3556.01] 2.93 2.84 13.93 128 3.62(2) 50

C(36) -H(36B) [ 3] -> Cg(6) [ 7555.01] 2.82 2.79 8.66 135 3.59(2) 53

[ 3555] = X,1/2-Y,1/2-Z

[ 3556] = X,1/2-Y,3/2-Z

[ 7555] = -X,1/2+Y,1/2+Z

Cg4 = centroid of ring N4-C26-C27-C28-C29-C30

Cg5 = centroid of ring C1-C2-C3-C4-C5-C6

Cg6 = centroid of ring C18-C19-C20-C21-C22-C23

# 7. Photophysical properties


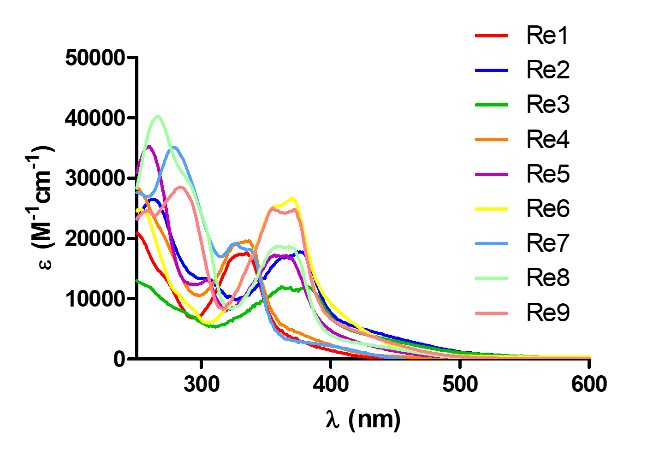


**Figure S38.** UV/vis spectra of complexes in aerated water (1% DMSO) at 20 ºC, 10 µM.


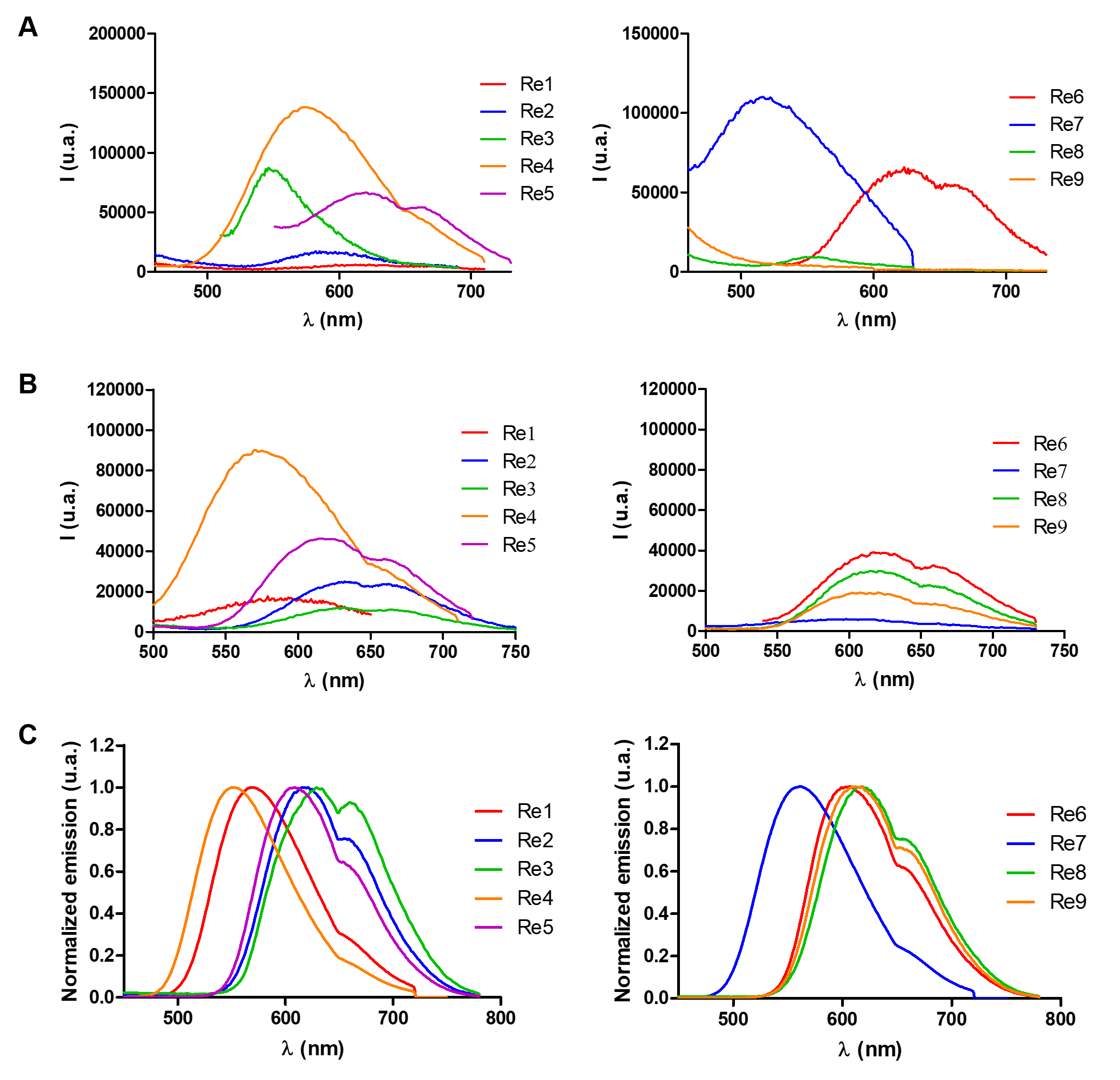


**Figure S39.** Emission spectra of complexes **Re1**–**Re9** in (A) aerated acetonitrile (B) aerated water (1% DMSO) and (C) solid powder, λexc(max) at the wavelength of their maximum absorption (330–380 nm), 10 µM for solutions.

**Table S8.** Absorption and emission wavelengths of complexes **Re1**–**Re9** in acetonitrile and water (1% DMSO) aerated and solid powder.

|  | **Acetonitrile** | | | **Water (1% DMSO)** | | | **Solid powder** | | |
| --- | --- | --- | --- | --- | --- | --- | --- | --- | --- |
| **Comp** | **λ (Ԑ, M-1cm-1)** | **λexc** | **λem** | **λ (Ԑ, M-1cm-1)** | **λexc** | **λem** | **λexc** | **λem** |
| **Re1** | 241 (32660), 280 (14220), 327 (20490), 342 (19080), 385 (4100) | 330 | - | 275 (12600), 328 (17220), 338 (17050), 388 (2190) | 330 | 590 | 370 | 570 |
| **Re2** | 263 (40850), 305 (16250), 360 (23610), 370 (23730), 413 (4200) | 380 | - | 265 (26440), 307 (13130), 367 (16980), 380 (17420), 430 (4690) | 380 | 630 | 400 | 620 |
| **Re3** | 256 (21870), 288 (10410), 357 (27550), 374 (27450), 417 (3770) | 330 | 550 | 289 (7490), 366 (11680), 385 (11710), 433 (3770) | 380 | 630 | 400 | 630 |
| **Re4** | 246 (32980), 328 (20960), 340 (20380), 383 (4800) | 330 | 580 | 275 (18660), 329 (19180), 339 (19000), 384 (3600) | 360 | 570 | 370 | 550 |
| **Re5** | 263 (40850), 307 (15180), 361 (21280), 372 (20780), 420 (3470) | 370 | 630 | 262 (34660), 307 (12420), 358 (17000), 371 (16400), 422 (2730) | 365 | 620 | 370 | 610 |
| **Re6** | 257 (23600), 361 (26050), 373 (26630), 427 (4730) | 370 | 630 | 287 (9520), 358 (25220), 373 (26080), 422 (5270) | 370 | 620 | 370 | 605 |
| **Re7** | 249 (27260), 281 (33810), 329 (19290), 342 (18790), 393 (2790) | 320 | 520 | 282 (34590), 330 (18570), 340 (17540), 400 (2180) | 370 | 600 | 370 | 560 |
| **Re8** | 265 (56450), 295 (37380), 359 (26020), 373 (25380), 431 (3660) | 320 | - | 270 (39290), 295 (27150), 360 (18740), 373 (17890), 440 (2040) | 370 | 620 | 370 | 620 |
| **Re9** | 259 (27500), 289 (32550), 359 (28150), 375 (26730), 439 (3710) | 360 | - | 287 (28140), 358 (24700), 374 (24210), 437 (3370) | 370 | 620 | 370 | 620 |

# 8. Stability studies


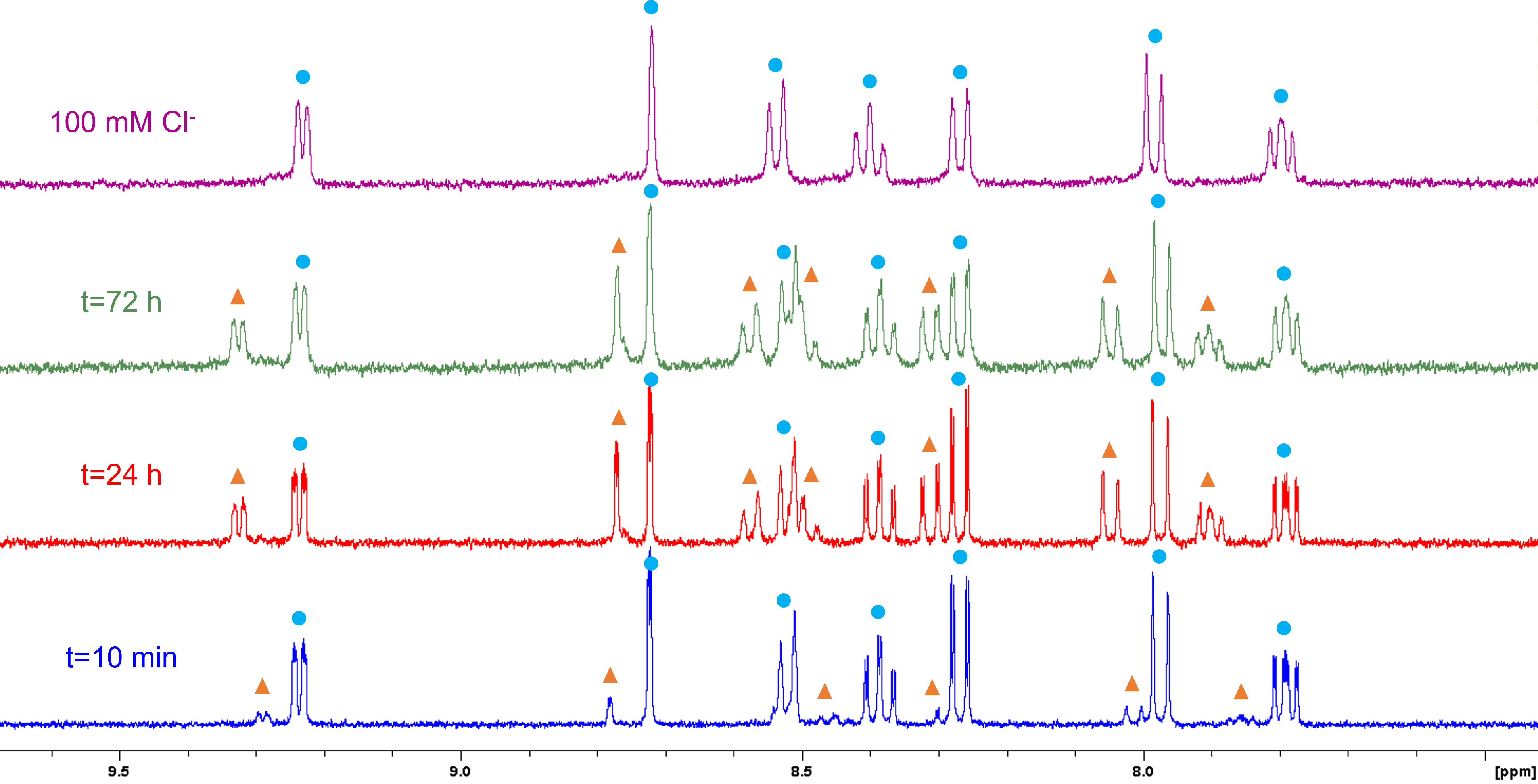


**Figure S40.** Reversible aquation and anation of **Re1**. 1H NMR spectra (400 MHz) of **Re1** (1 mM) in MeOD-d4 at r.t. After 10 min, 24 h and 72 h (blue, red and green lines, respectively), and after 72 hours and the addition of NaCl in MeOD-d4 (100 mM) (purple line).

**Figure S41.** UV/vis spectra of complexes **Re1**–**Re9** (10 µM) in DMSO at t=0 h and after 48 h.


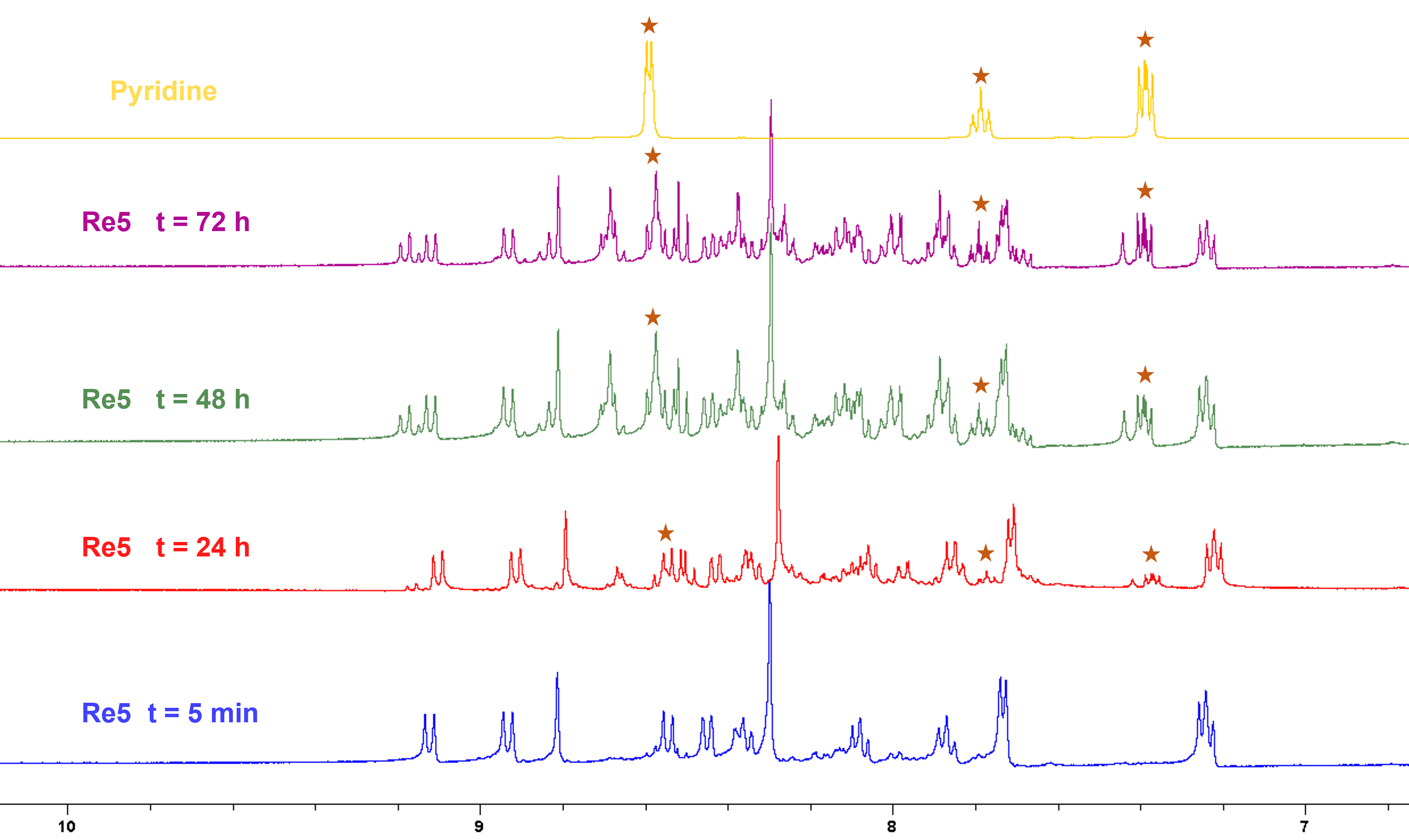


**Figure S42.** 1H NMR spectra of **Re5** at different time and pyridine (yellow) in DMSO-d6 at r.t., 400 MHz.


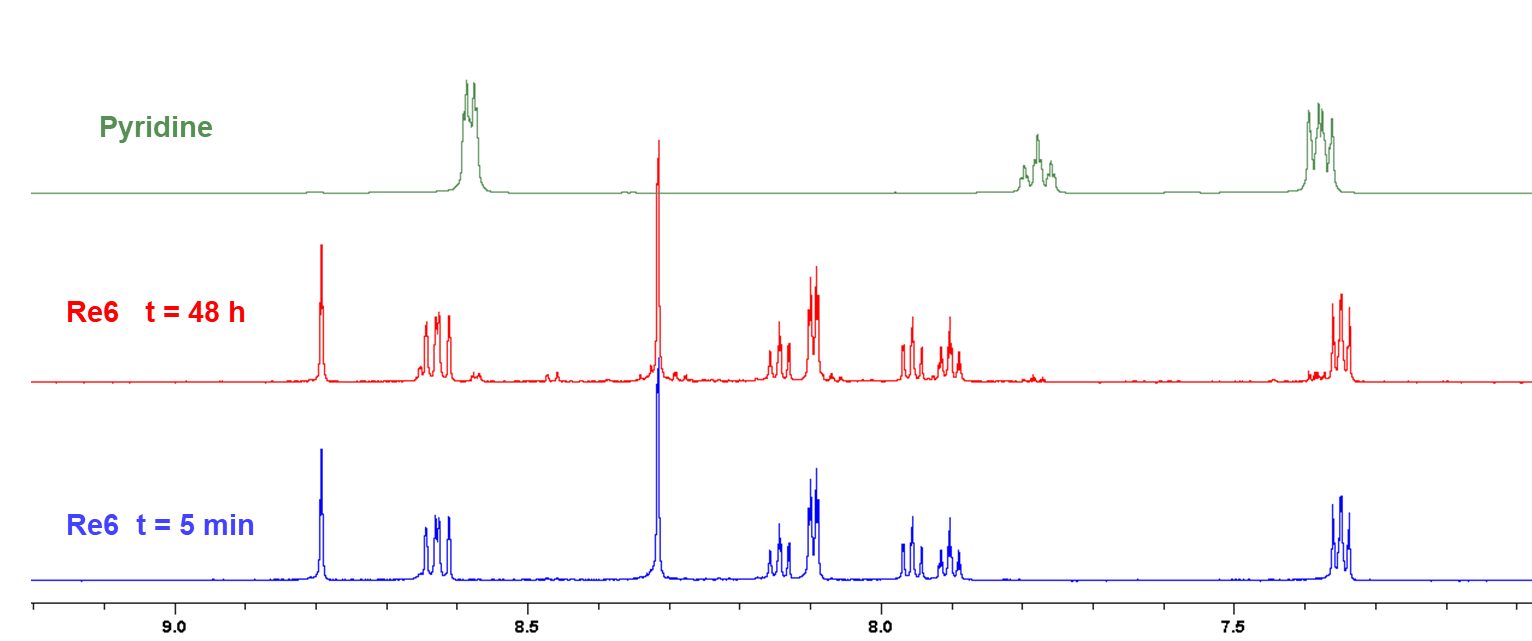


**Figure S43.** 1H NMR spectra of **Re6** at different time and pyridine (green) in DMSO-d6 at r.t., 600 MHz.


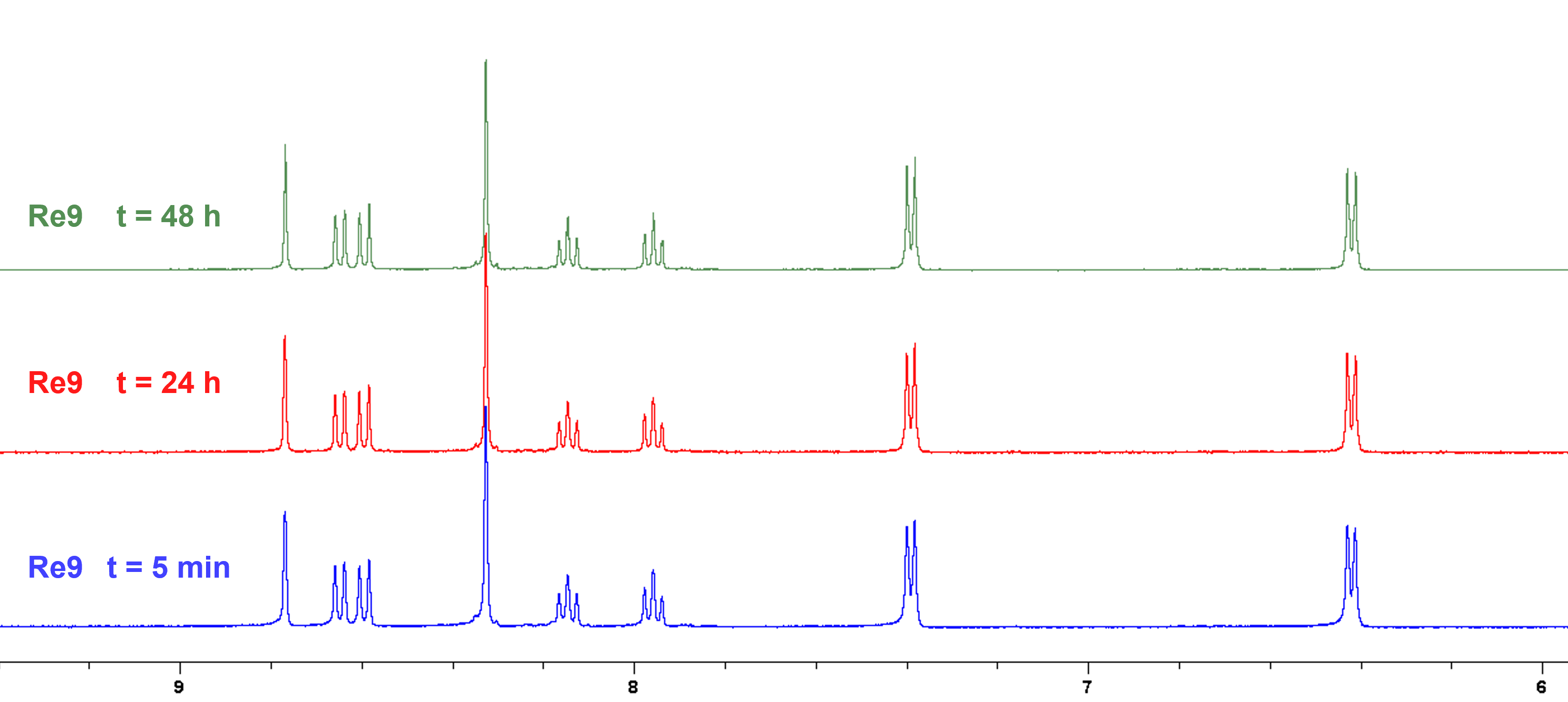


**Figure S44.** 1H NMR spectra of **Re9** at different time in DMSO-d6 at r.t., 400 MHz.

**Figure S45.** UV/vis spectra of complexes **Re1**–**Re8** (10 µM) in RPMI (5% DMSO) at t=0 and after 48 h.


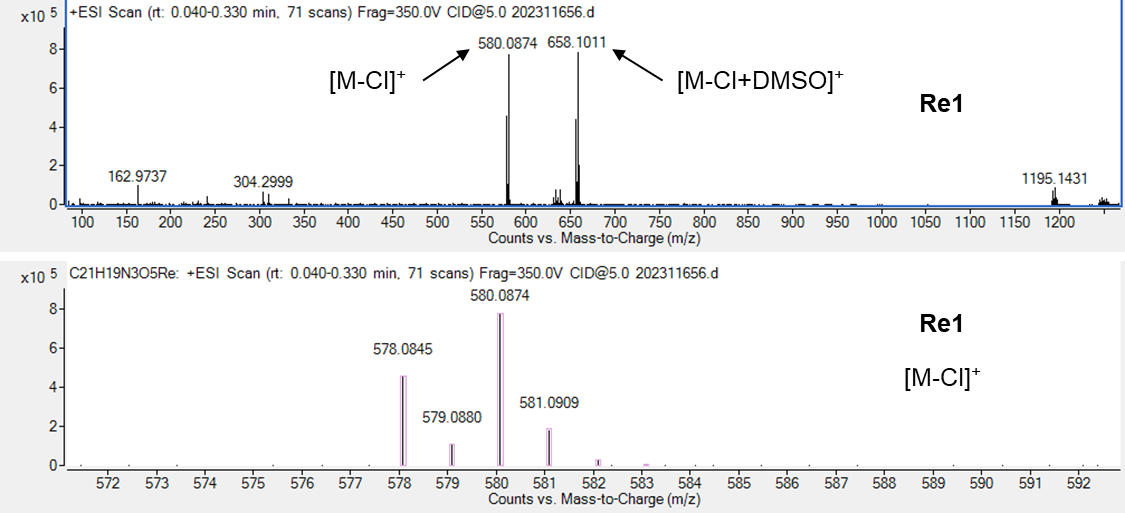


**Figure S46.** ESI-HRMS spectrum of **Re1** in positive ion mode in DMSO.


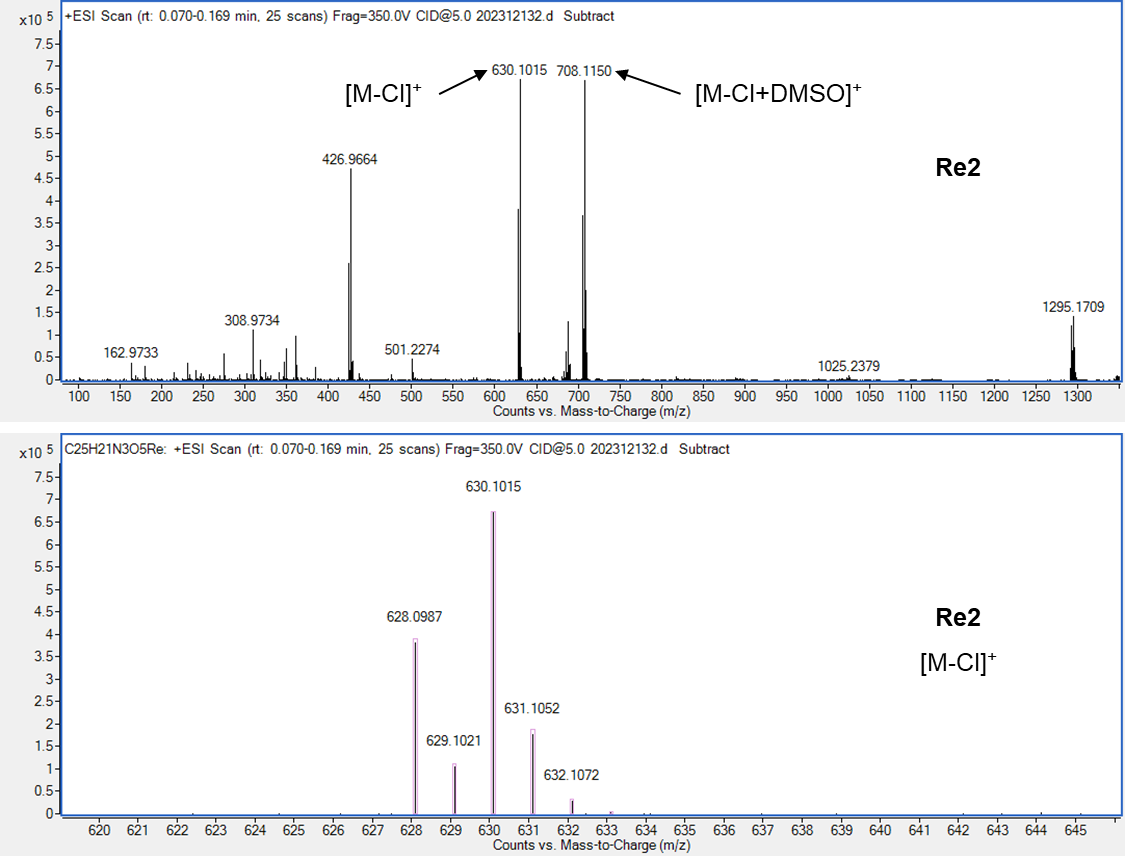


**Figure S47.** ESI-HRMS spectrum of **Re2** in positive ion mode in DMSO.


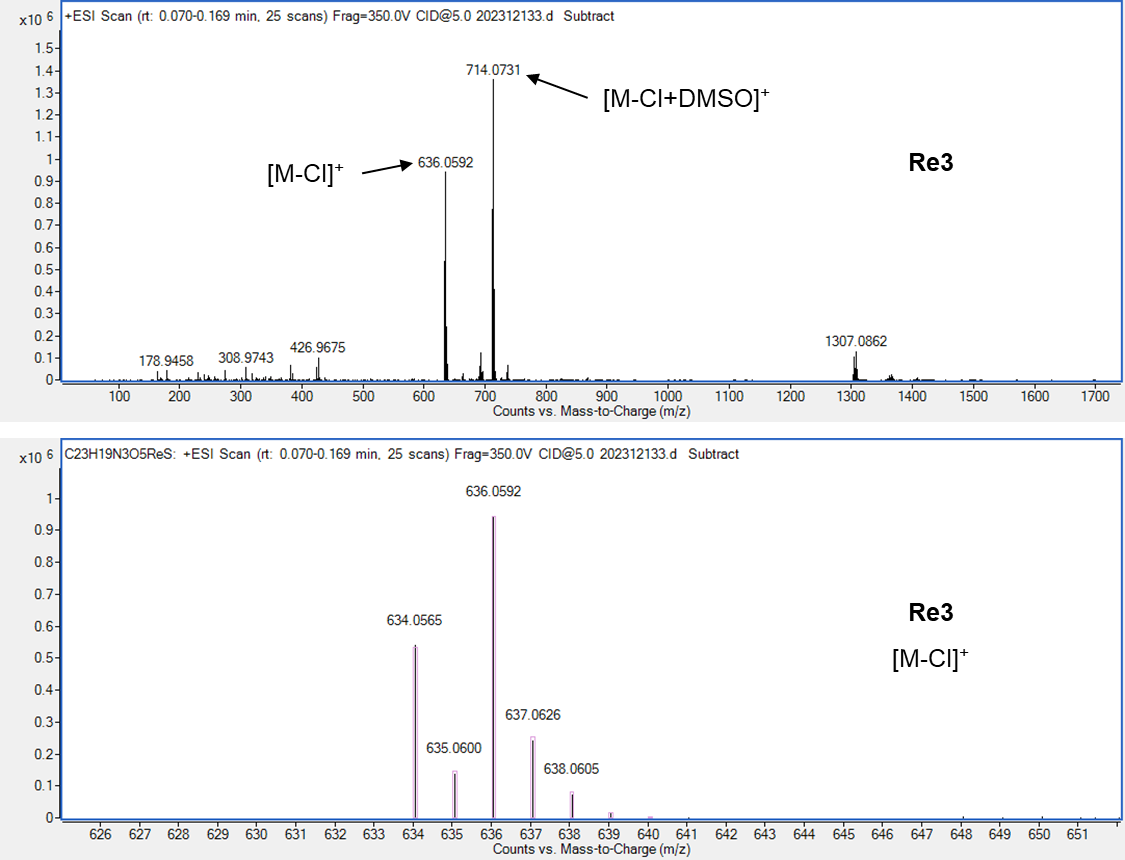


**Figure S48.** ESI-HRMS spectrum of **Re3** in positive ion mode in DMSO.

# 9. Scanning electron microscopy

| A) | B) |
| --- | --- |
| 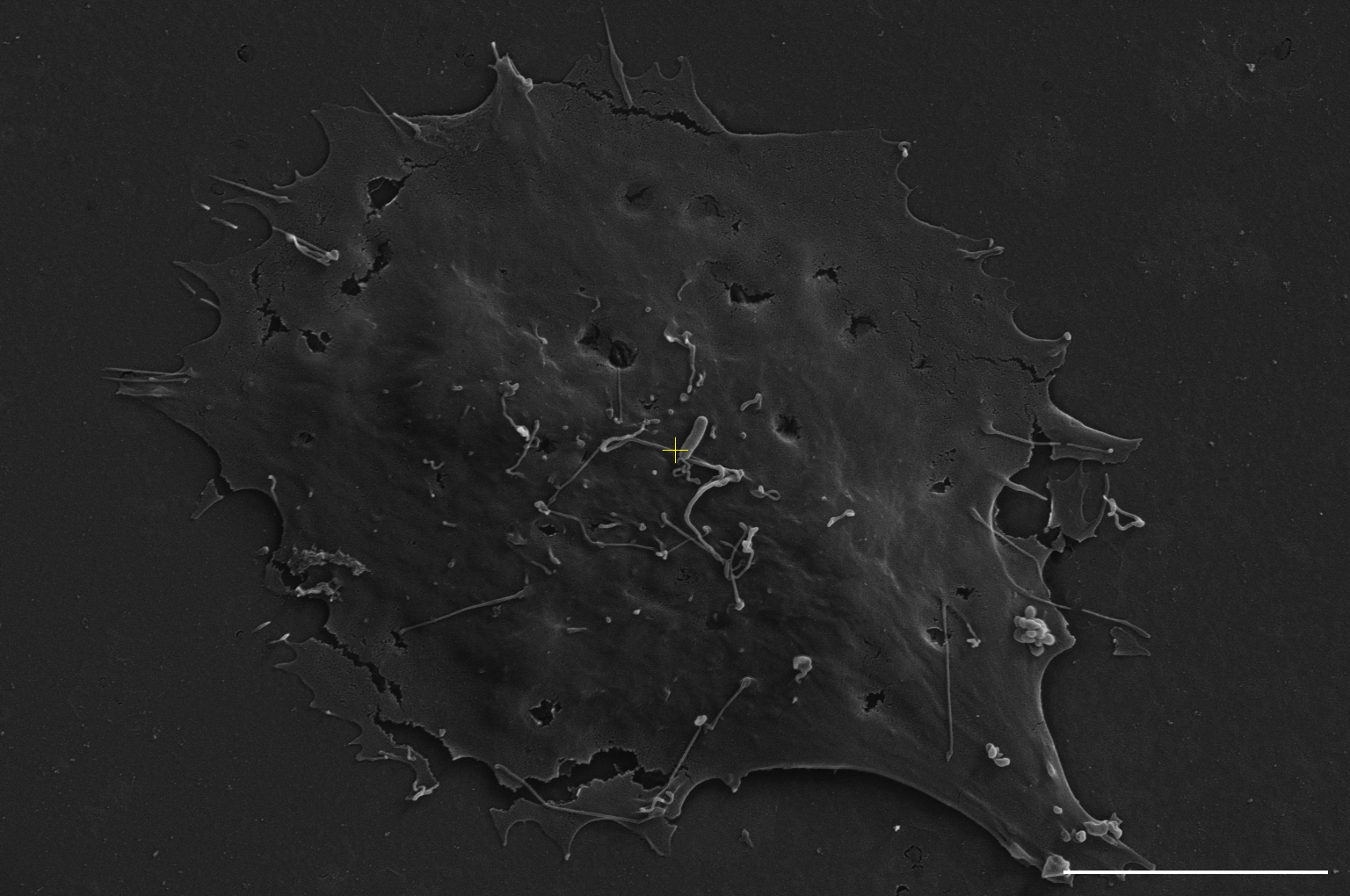 | 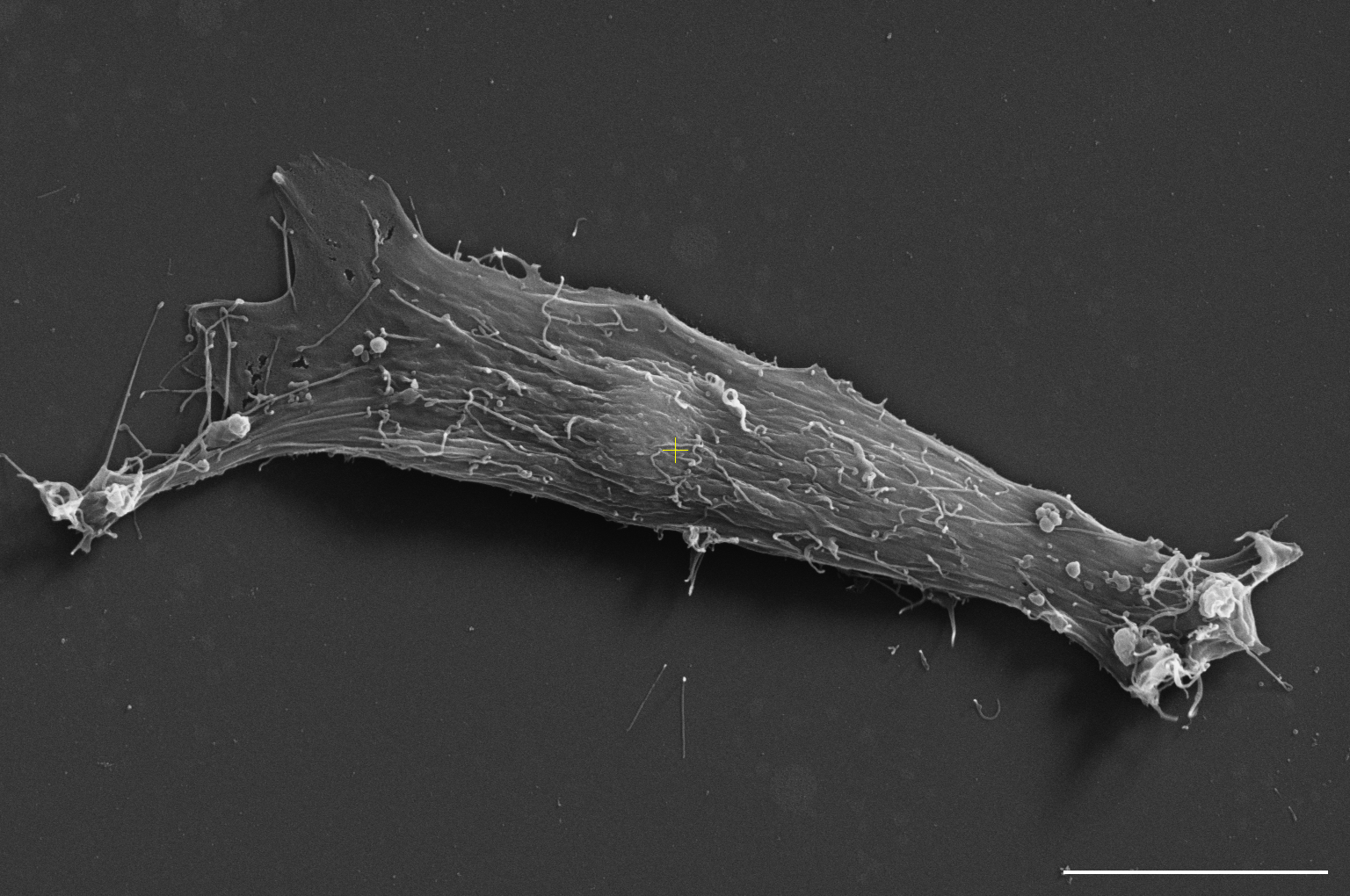 |
| C) | D) |
| 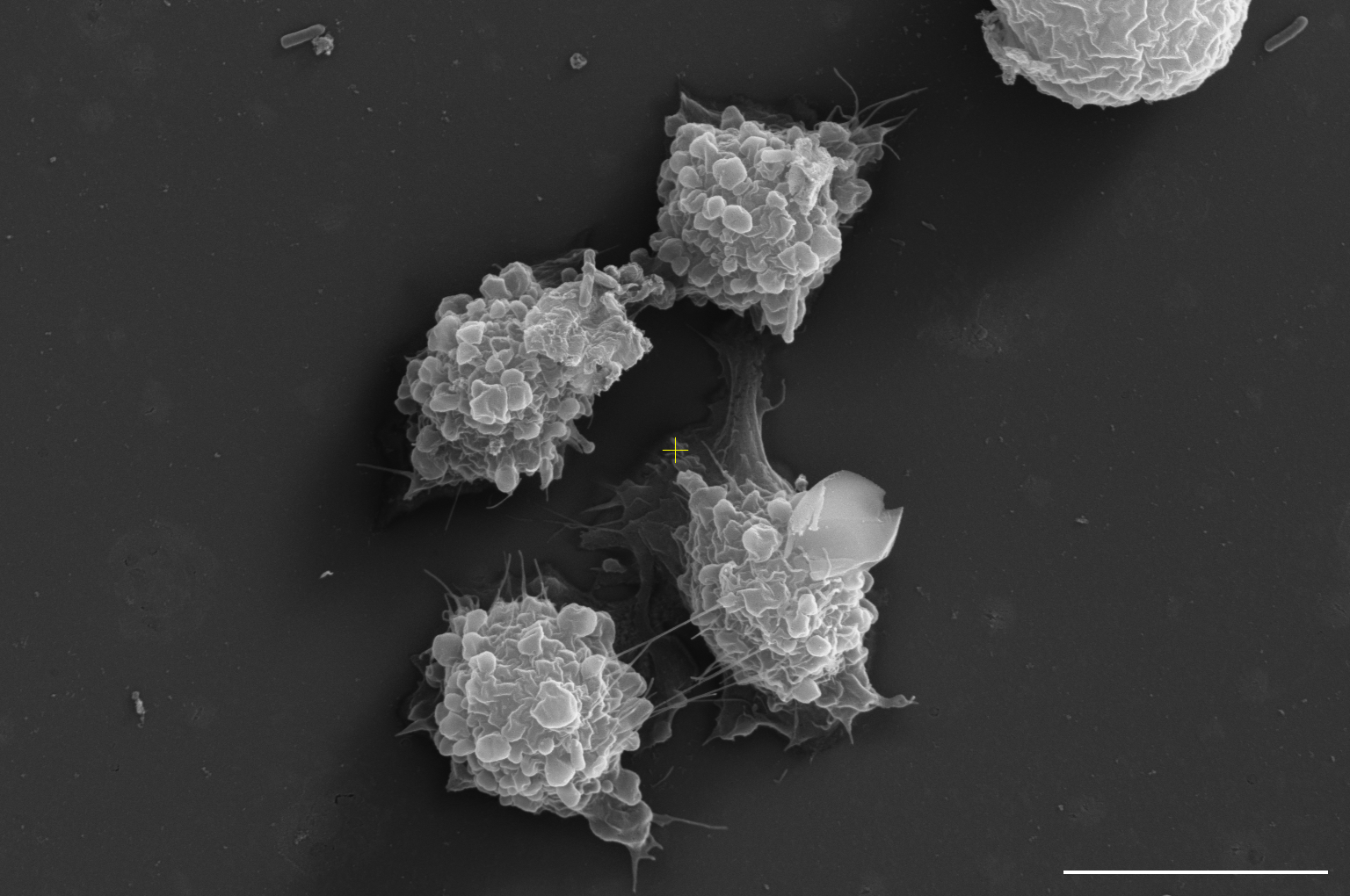 | 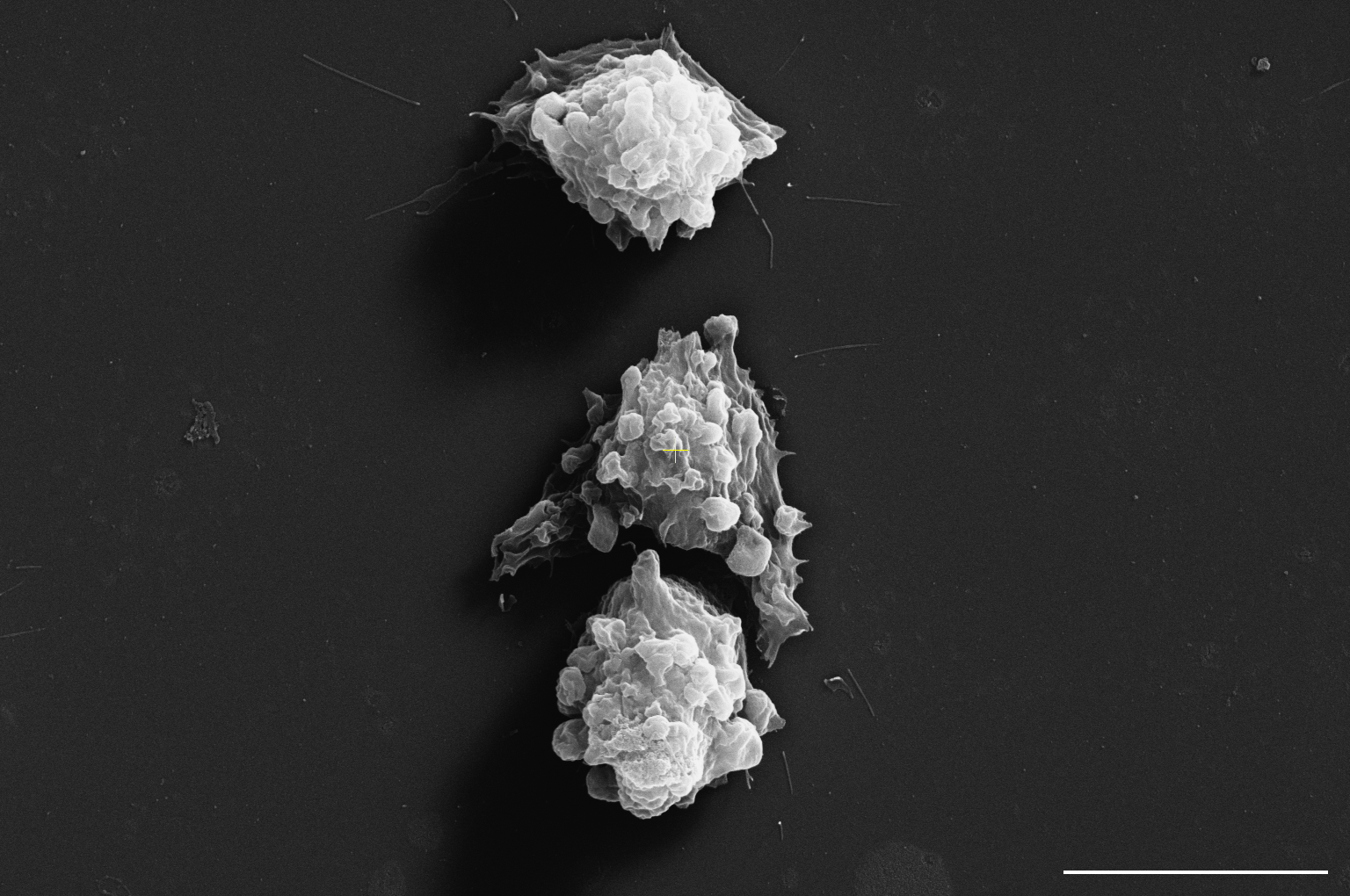 |
| E) | F) |
| 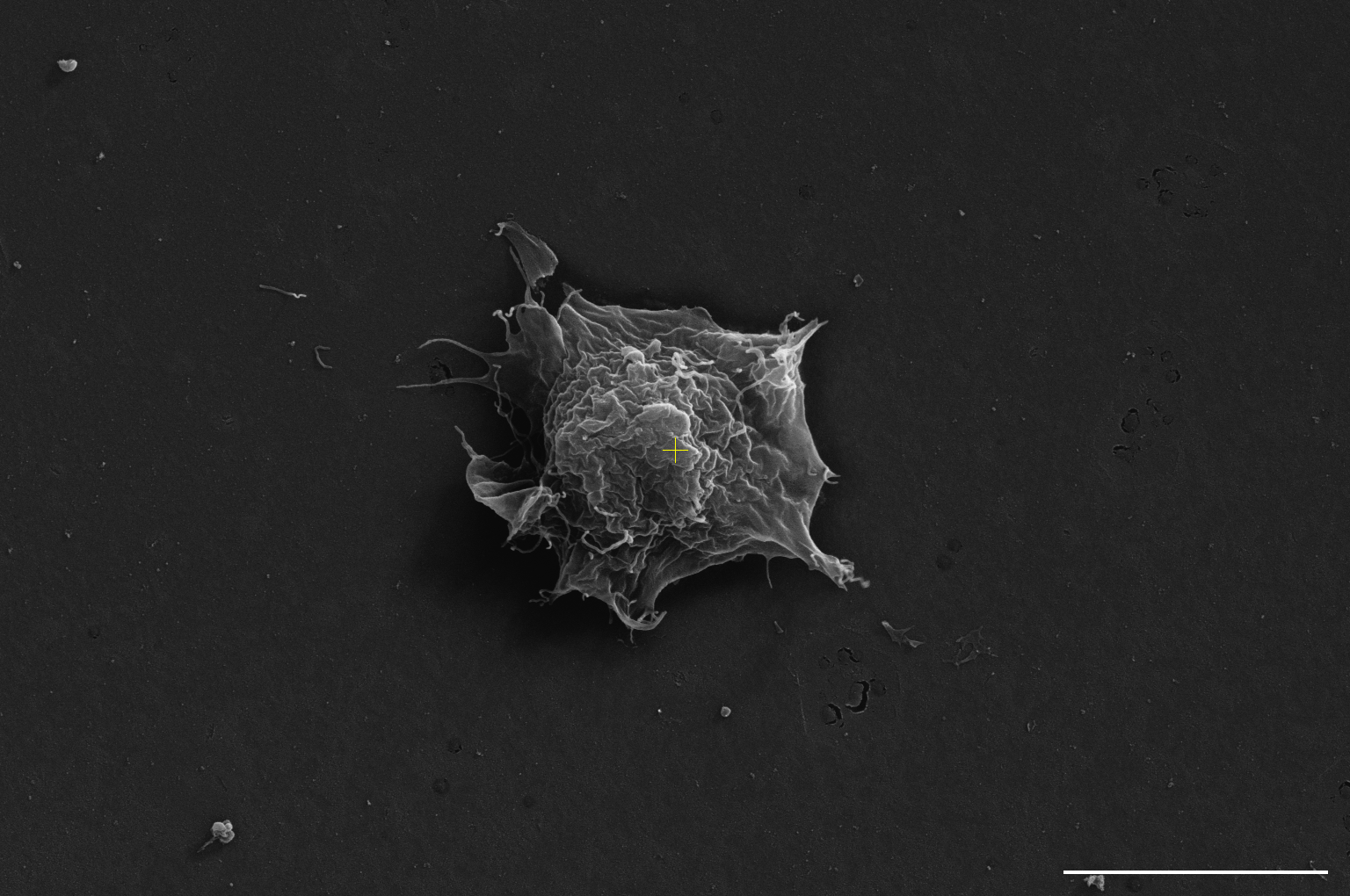 | 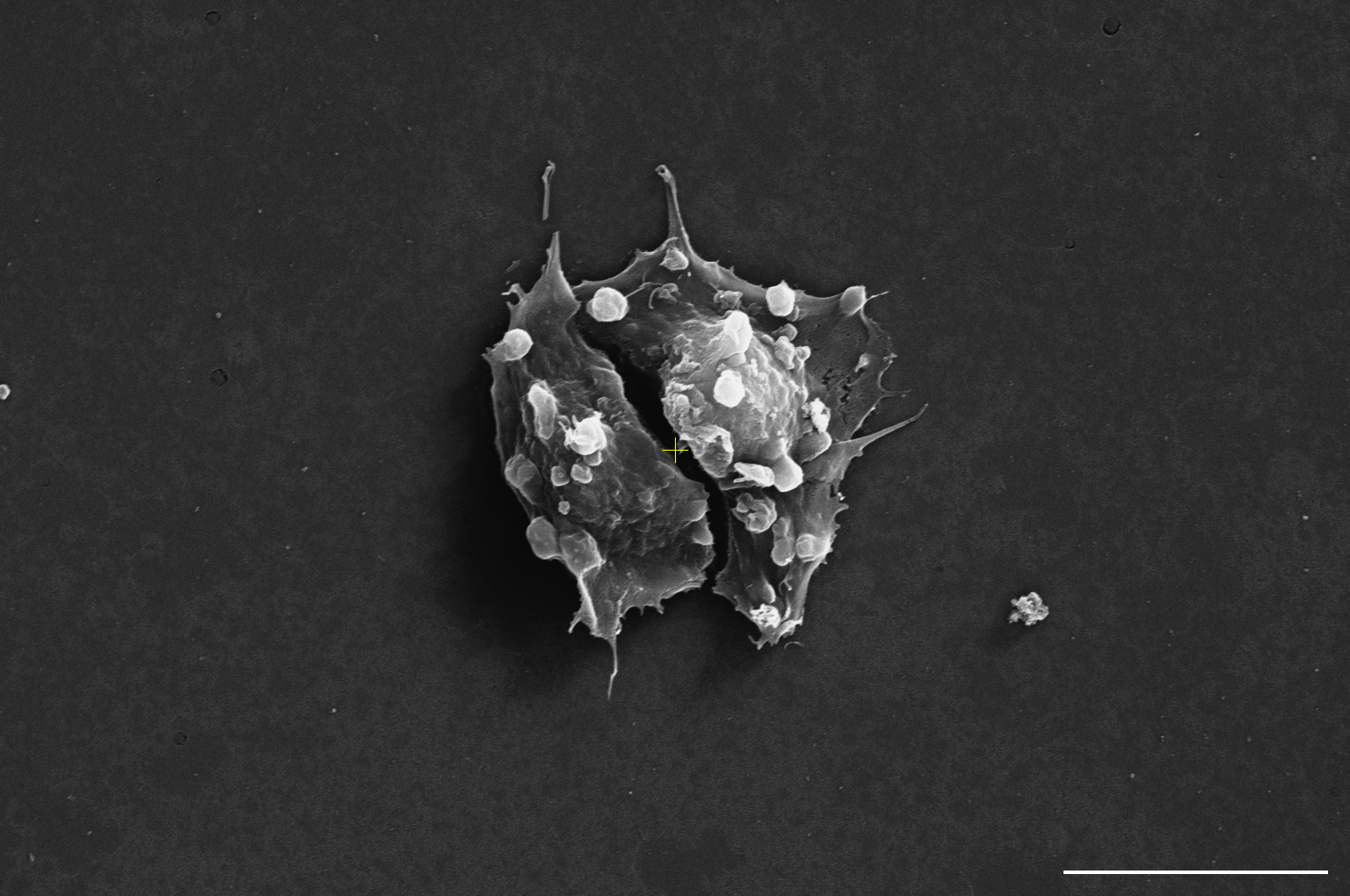 |

**Figure S49.** Scanning electron microscopy of A2780 cells indicating cellular morphological features during apoptosis (cisplatin treatment) and pyroptosis (**Re9** treatment). Cells were either untreated (A-B), exposed to cisplatin (C-D), or treated with **Re9** (E-F)

# 10. Cell death study


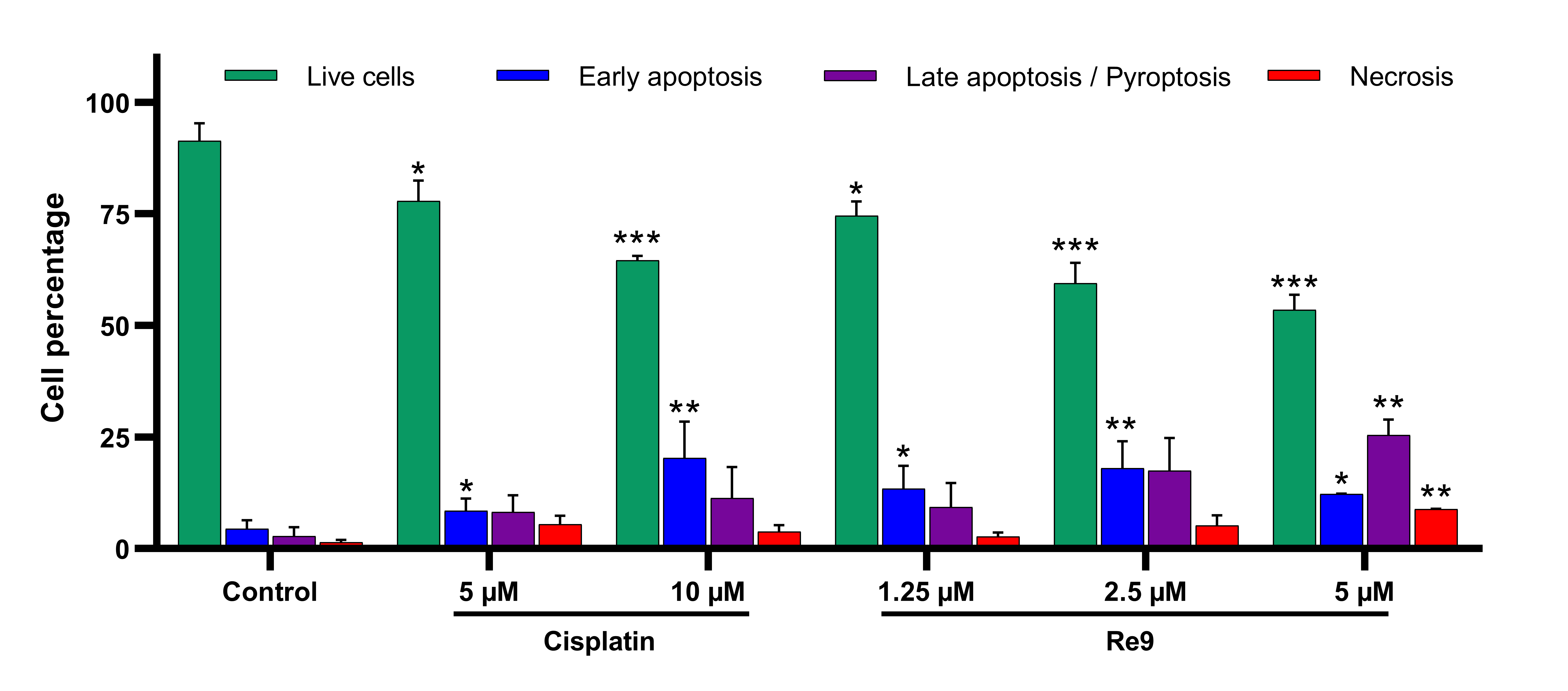


**Figure S50.** Bar graph presented in percentage of the cells. Data expressed as mean ± SD from three replicates. An independent unpaired t-test was used to define statistical differences between the obtained values (*p<0.05, **p<0. 01, ***p<0.001). Each data set has been statistically compared to its control value.

# 11. References

(1) Yellol, J.; Pérez, S. A.; Yellol, G.; Zajac, J.; Donaire, A.; Vigueras, G.; Novohradsky, V.; Janiak, C.; Brabec, V.; Ruiz, J. Highly Potent Extranuclear-Targeted Luminescent Iridium( iii ) Antitumor Agents Containing Benzimidazole-Based Ligands with a Handle for Functionalization. *Chem. Commun.* **2016**, *52* (98), 14165–14168. https://doi.org/10.1039/C6CC07909A.

(2) Ortega-Forte, E.; Hernández-García, S.; Vigueras, G.; Henarejos-Escudero, P.; Cutillas, N.; Ruiz, J.; Gandía-Herrero, F. Potent Anticancer Activity of a Novel Iridium Metallodrug via Oncosis. *Cell. Mol. Life Sci.* **2022**, *79* (10), 510. https://doi.org/10.1007/s00018-022-04526-5.

(3) Spek, A. L. Structure Validation in Chemical Crystallography. *Acta Cryst D* **2009**, *65* (2), 148–155. https://doi.org/10.1107/S090744490804362X.

(4) Spek, A. L. A Multipurpose Crystallographic Tool. *Utrecht, The Netherlands* **2005**.

(5) Yang, X.-J.; Drepper, F.; Wu, B.; Sun, W.-H.; Haehnel, W.; Janiak, C. From Model Compounds to Protein Binding: Syntheses, Characterizations and Fluorescence Studies of [Ru II (Bipy)(Terpy)L] 2+ Complexes (Bipy = 2,2′-Bipyridine; Terpy = 2,2′:6′,2″-Terpyridine; L = Imidazole, Pyrazole and Derivatives, Cytochrome c). *Dalton Trans.* **2005**, No. 2, 256–267. https://doi.org/10.1039/B414999H.

(6) Janiak, C. A Critical Account on π–π Stacking in Metal Complexes with Aromatic Nitrogen-Containing Ligands †. *J. Chem. Soc., Dalton Trans.* **2000**, No. 21, 3885–3896. https://doi.org/10.1039/b003010o.

(7) Nishio, M. The CH/π Hydrogen Bond in Chemistry. Conformation, Supramolecules, Optical Resolution and Interactions Involving Carbohydrates. *Phys. Chem. Chem. Phys.* **2011**, *13* (31), 13873. https://doi.org/10.1039/c1cp20404a.

(8) Nishio, M.; Umezawa, Y.; Honda, K.; Tsuboyama, S.; Suezawa, H. CH/π Hydrogen Bonds in Organic and Organometallic Chemistry. *CrystEngComm* **2009**, *11* (9), 1757. https://doi.org/10.1039/b902318f.

(9) Nishio, M. CH/? Hydrogen Bonds in Crystals. *CrystEngComm* **2004**, *6* (27), 130. https://doi.org/10.1039/b313104a.

(10) Janiak, C.; Temizdemir, S.; Dechert, S.; Deck, W.; Girgsdies, F.; Heinze, J.; Kolm, M. J.; Scharmann, T. G.; Zipffel, O. M. Binary [Hydrotris(indazol-1-yl)borato]metal Complexes, M(Tp4Bo)2[1] with M = Fe, Co, Ni, Cu, and Zn: Electronic Properties and Solvent-Dependent Framework Structures through C−H···π Interactions. *European Journal of Inorganic Chemistry* **2000**, *2000* (6), 1229–1241. https://doi.org/10.1002/(SICI)1099-0682(200006)2000:6<1229::AID-EJIC1229>3.0.CO;2-P.

(11) Umezawa, Y.; Tsuboyama, S.; Honda, K.; Uzawa, J.; Nishio, M. CH/π Interaction in the Crystal Structure of Organic Compounds. A Database Study. *BCSJ* **1998**, *71* (5), 1207–1213. https://doi.org/10.1246/bcsj.71.1207.

(12) *The CH/&pi; Interaction: Evidence, Nature, and Consequences | Wiley*. Wiley.com. https://www.wiley.com/en-us/The+CH+%26pi%3B+Interaction%3A+Evidence%2C+Nature%2C+and+Consequences-p-9780471252900 (accessed 2023-07-19).
